# Zwitterionic Dipeptide Surface Functionalization of Detonation Nanodiamond for Enhanced Control in Biological Environments

**DOI:** 10.1002/anie.202501202

**Published:** 2025-05-19

**Authors:** Elisabeth Mayerhoefer, Himalaya Parajuli, Mihaela‐Roxana Cimpan, Daniela Elena Costea, Harsh Nitin Dongre, Anke Krueger

**Affiliations:** ^1^ Institute of Organic Chemistry University of Stuttgart Pfaffenwaldring 55 70569 Stuttgart Germany; ^2^ The Gade Laboratory for Pathology and Centre for Cancer Biomarkers (CCBIO) Department of Clinical Medicine University of Bergen Jonas Lies vei 87 Bergen 5021 Norway; ^3^ Department of Pathology Haukeland University Hospital Jonas Lies vei 65 Bergen 5021 Norway; ^4^ Department of Clinical Dentistry University of Bergen Årstadveien 19 Bergen 5009 Norway; ^5^ Center for Integrated Quantum Science and Technology University of Stuttgart Allmandring 3 70569 Stuttgart Germany

**Keywords:** Biocompatibility, Nanoparticles, Peptides, Surface chemistry, Zwitterions

## Abstract

Utilizing nanoparticles as innovative theranostic agents for biomedical applications requires full control over the material's properties to dictate their interactions within a biological environment. Owing to its versatile surface chemistry and high biocompatibility, nanodiamond (ND) represents a promising platform for novel healthcare treatments. To ensure the performance and safety of NDs, their properties and behavior must remain unchanged upon administration, a key challenge in nanomedicine. Recently, zwitterionic surface modifications have emerged as new strategies to substantially improve protein‐repelling properties and biocompatibility of nanomaterials. Using for the first time covalently conjugated zwitterionic dipeptides as surface modulators for ND particles, we were able to provide a readily accessible, reproducible, and tunable functionalization. The obtained particles demonstrate enhanced colloidal stability and conservation of particle size over a broad pH range and in different protein‐containing media compared to the starting material. By simple selection of different dipeptides, we can carefully tailor the biocompatibility and cellular uptake of functionalized NDs. We reveal the functionalized NDs’ behavior in biologically relevant 3D organotypic models and how different dipeptide‐functionalized NDs interact with squamous epithelium ex vivo. The results pave the way for various applications, e.g., biosensing, tissue engineering or targeted drug delivery of these highly biologically suitable nanoparticles.

## Graphical abstract

Here, we report a systematic investigation of novel zwitterionic dipeptide functionalized nanodiamonds to ensure colloidal stability over a broad pH range and in biological media. In vitro experiments in 2D and 3D cell cultures revealed a dipeptide‐dependent biocompatibility and cellular uptake.

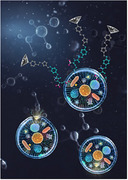

## Introduction

ND particles designed for biomedical applications require defined physicochemical properties to exhibit a favorable and predictable behavior in biological systems.^[^ ^1^, ^2^, ^3^, ^4^ ^]^ Several essential properties, such as particle size, dispersibility, or non‐toxicity, can be influenced by functionalizing the surface of the particle in a controlled manner. Consequently, the ultimate objective of surface modification is to extend its purpose beyond solely offering a suitable platform for the covalent or non‐covalent binding of functional elements toward full control of interactions and the fate of the particles.

Upon exposure to physiological environments, nanoparticles encounter a great number of different biomolecules such as proteins, lipids and, other cellular components.^[^ ^5^, ^6^ ^]^ Typically, these biomolecules adhere to the surface of the nanoparticles, creating a dynamic layer commonly referred to as “protein corona”.^[^ ^5^, ^6^, ^7^ ^]^ Although protein corona formation improves the biocompatibility of nanomaterials, this process also conceals functional elements attached to the particle surface making them inaccessible to their biological target.^[^ ^8^, ^9^ ^]^ Moreover, it affects agglomeration, resulting in different mechanism of cellular internalization.^[^ ^10^, ^11^ ^]^ Nanoparticles with protein corona are recognized by the reticuloendothelial system (RES), which initiates their opsonization and scavenging.^[^ ^12^ ^]^ Prevention of protein corona formation by surface functionalization therefore enables nanoparticles to escape RES and results in prolonged blood circulation times and reduced “off‐target” effects.^[^ ^13^, ^14^ ^]^

In recent times, surface coatings with zwitterionic low‐molecular weight compounds have become an important method for the production of safe and reliable theranostic agents.^[^ ^5^, ^11^, ^15^, ^16^ ^]^ For this type of modification, the interaction between the nanomaterial and the surrounding environment originates from strong electrostatic interactions providing improved colloidal stability and protein repulsion. Furthermore, zwitterion decorated nanoparticles offer the possibility to be eliminated from the body via renal excretion assuming the original particle size is small enough (5–6 nm) for glomerular filtration in the kidneys.^[^ ^5^, ^17^, ^18^ ^]^ In the case of ^18^F‐radiolabeled ND particles, it could be demonstrated that clearance of primary particles with sizes ∼7 nm takes place via the urinary tract, while larger particles or agglomerates are trapped and/or phagocytized.^[^ ^19^ ^]^ To ensure the retention of small particle sizes required for renal excretion, zwitterionic surface modifications can be employed to enhance colloidal stability and hydrophilicity as well as prevent nonspecific protein adsorption and agglomeration.

Among the class of small zwitterionic molecules, some amino acids have already been used as surface functionalization of inorganic or modified chitosan nanoparticles for different applications.^[^ ^20^, ^21^, ^22^, ^23^ ^]^ The charged surface groups of amino acid decorated nanoparticles play a key role in dispersibility and particle size, essential parameters that directly affect cellular uptake, biodistribution, and elimination.^[^ ^20^ ^]^ Nanoparticles showing a high colloidal stability and small particle sizes are less likely to form agglomerates when exposed to biologically relevant media.^[^ ^24^, ^25^ ^]^ The particle size not only determines the amount of internalized particles but also the uptake mechanism.^[^ ^26^, ^27^ ^]^

In addition to the ability of amino acids to carry charges and be biocompatible, they further show a pH‐responsive behavior. This is particularly important as variations of the pH occur across different cellular compartments as well as in cancerous tissue.^[^ ^28^, ^29^ ^]^

In the present study, we report the surface functionalization of detonation nanodiamond (ND) with three different zwitterionic dipeptides to identify correlations between the conjugated incorporated amino acid and its cellular effect in terms of cytotoxicity and intracellular localization. In contrast to our already established protocol toward deagglomerated ND particles exhibiting remarkable protein repulsion in biofluids by hierarchically combining structural elements with terminating zwitterionic moieties,^[^ ^11^, ^30^ ^]^ we now present a completely novel and efficient approach. Here, different zwitterionic dipeptides based on L‐tyrosine conjugated with another natural L‐amino acid (glutamic acid, leucine, and lysine) were covalently immobilized on ND to maintain colloidal stability and repel nonspecific protein adsorption without increasing the hydrodynamic diameter of the particles by attachment of spatially extended molecules.

Since usual conjugation strategies imply losing either the free amino/carboxyl group or the amino acid‐specific side chain, dipeptides were formed to specifically preserve these functional groups. The resulting dipeptide functionalized NDs were systematically investigated in solutions of varying pH values and in vitro. With these findings in hand, we could identify particular dipeptides yielding stable, protein corona‐free ND particles exhibiting low toxicity and efficient uptake. This method opens the way to more complex functionalization patterns using a multimodal approach combining protein repelling properties with additional functionalities such as targeting moieties or drugs for enhanced control in biological environments.

## Results and Discussion

### Synthesis and Characterization

Apart from widely used zwitterionic moieties like carboxybetaines and sulfobetaines, amino acids represent another prominent group of low‐weight molecules with positive and negative charges under physiological conditions. Several nanoparticles including cysteine‐conjugated silica nanoparticles,^[^ ^31^ ^]^ arginine‐modified chitosan nanoparticles^[^ ^32^ ^]^ as well as nanodiamonds functionalized with glycine or phenyl alanine have been reported.^[^ ^33^ ^]^ In some of these examples, the amino acids were directly attached at their *N*‐terminus, thus leading to an anionic surface coating under physiological conditions. Otherwise, the amino acid side chain was used to form the connection, thereby losing its specific properties toward potential biological targets.

**Scheme 1:**
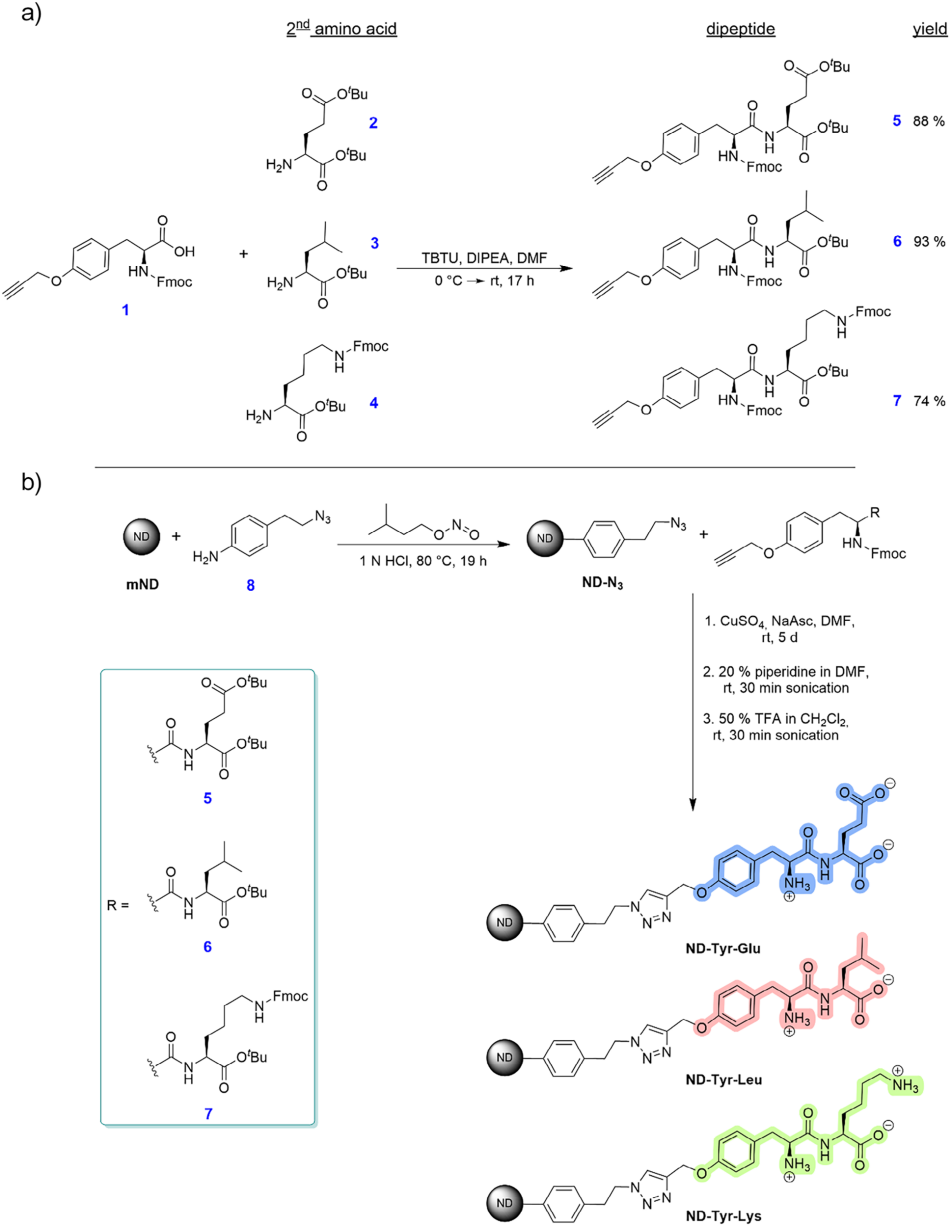
a) Synthesis of three different “clickable” dipeptides **5**, **6,** and **7** from tyrosine derivative **1** and the respective second amino acid (**2**, **3,** or **4**) with the help of a peptide coupling reagent (TBTU, 2‐(1*H*‐benzotriazole‐1‐yl)‐1,1,3,3‐tetramethylaminium tetrafluoroborate) and a base (DIPEA, *N*,*N*‐diisopropylethylamine). b) Summarized synthetic strategy and reaction conditions toward zwitterionic dipeptide functionalized ND particles **ND‐Tyr‐Glu**, **ND‐Tyr‐Leu,** and **ND‐Tyr‐Lys** starting from azide modified **ND‐N_3_** by applying “click” chemistry.

To preserve the zwitterionic groups of the amino acid backbone as well as the specific side chain, fluorenylmethyloxycarbonyl (fmoc)‐protected alkyne‐modified tyrosine **1** (Scheme 1a) was chosen as the connecting unit between the terminal amino acid and azide functionalized detonation nanodiamond **ND‐N_3_**. The coupling of alkyne‐modified tyrosine and a second amino acid afforded “clickable” dipeptides via the side chain of tyrosine. Amino acids incorporating ionizable groups within their specific side chains are of particular interest since they exhibit different types of ionization under physiological conditions. Therefore, glutamic acid and lysine were chosen as second amino acid, each of them representing a different subgroup of amino acids while showing structural similarities (alkyl side chain). As uncharged alternative, leucine was selected.

The peptide bond was selectively formed between the carboxyl group of **1** and the α‐amino group of the second amino acid, using the protected amino acids glutamic acid **2**, leucine **3**, and lysine **4**. The dipeptide synthesis was performed under inert conditions using the peptide coupling reagent TBTU (Scheme 1a). The resulting dipeptides ≡─O‐Tyr‐Glu **5**, ≡─O‐Tyr‐Leu **6** and ≡─O‐Tyr‐Lys **7** were obtained in yields of 88%, 93%, and 74%, respectively.

Before the covalent immobilization of functional moieties on the nanodiamond surface, pretreatment of pristine ND is essential. Depending on the intended application, it comprises several processing steps, in our case mechanical deagglomeration and subsequent functionalization with a short organic linker molecule.^[^ ^34^, ^35^ ^]^ Deagglomerated particles form stable colloidal dispersions and present a higher surface accessibility enabling a homogeneous initial functionalization.^[^ ^34^, ^35^, ^36^ ^]^ We achieved mechanical disintegration of detonation ND powder into primary particles of <10 nm in size by stirred‐media milling according to previously reported procedures.^[^ ^11^, ^36^ ^]^

In the following step, the grafting of a short aryl linker **8** (Scheme 1b) with a terminal azide group onto milled detonation nanodiamond (**mND**) via a radical arylation reaction was accomplished, which afforded azide functionalized nanodiamond **ND‐N_3_**.^[^ ^34^, ^35^ ^]^ To confirm the successful attachment, diffuse reflectance infrared Fourier transform spectroscopy (DRIFTS) and thermogravimetric analysis (TGA) was applied. The asymmetric azide stretch vibration at approximately 2100 cm^−1^ in the spectrum is clearly visible (see Figure S30).

The particles demonstrate good dispersibility in water, a zeta potential of *ζ *= +38 mV and a surface loading of 0.22 mmol g^−1^ measured by TGA. With **ND‐N_3_** in hand, we were able to covalently graft the synthesized dipeptides ≡─O‐Tyr‐Glu **5**, ≡─O‐Tyr‐Leu **6,** and ≡─O‐Tyr‐Lys **7** to ND through a Cu(I)‐catalyzed “click” reaction (Scheme 1b). “Click” chemistry not only provides a highly versatile conjugation technique but it also ensures reproducibility,^[^ ^37^ ^]^ which is a fundamental prerequisite when engineering nanomaterials for biomedical applications. During the reaction, the conjugation progress could be easily monitored using DRIFTS by the disappearance of the band at *ν* = 2100 cm^−1^ upon completion of the “click” reaction, while new dipeptide related vibrations appear in the respective spectra (see Figure S34). To establish the zwitterionic surface architecture, the obtained dipeptide functionalized ND particles were deprotected using a stepwise protocol for the removal of the fmoc and *tert*‐butyl ester protecting groups to obtain free amino and carboxyl groups that are able to carry charges.^[^ ^38^, ^39^ ^]^

Even though all of the attached dipeptides now present both, cationic and anionic groups, the overall number of positive and negative charges differs depending on the involved dipeptide‐forming amino acids. Thus, at physiological pH of 7.4, **ND‐Tyr‐Glu** carries a higher number of negatively charged carboxylate groups compared to **ND‐Tyr‐Lys**, where a higher number of positively charged ammonium groups is present on the surface. Zeta potential measurements of the respective ND dispersions (Table 1) showed highly positive values of around +35 mV at an intrinsic pH of 7.1. The starting material **mND** displays a zeta potential ranging from +45 to +33 mV between pH 5–8 that is assumed to originate from fragments of graphene layers and/or hydroxyl surface groups.^[^ ^40^, ^41^, ^42^ ^]^ Compared to **ND‐N_3_**, dipeptide functionalization seems to slightly decrease the zeta potential due to the presence of carboxylate groups. The effect of the differently charged surface moieties is also clearly evidenced in their interaction with cells (see below).

**Table 1:** Results of zeta potential (ζ) measurements, thermogravimetric analysis, and particle size distribution.

ND Material	Surface Loading (mmol g^−1^)	Zeta Potential ζ (mV) (pH)	*D*_v_ (10) (nm)^a)^	D_v_ (50) (nm)^a)^	D_v_ (90) (nm)^a)^	D_v_ (10) (nm)^b)^	D_v_ (50) (nm)^b)^	D_v_ (90) (nm)^b)^	Median Particle Size (nm)^c)^
**mND**	–	+33.3 (7.1)	4.49	6.67	12.8	61.0	286	5640	
**ND‐N_3_**	0.22	+38.2 (7.1)	39.7	58.2	99.6	88.4	324	5820	
**ND‐Tyr‐Glu**	0.08	+32.5 (7.1)	29.6	43.9	97.0	48.2	77.0	249	26.7
**ND‐Tyr‐Leu**	0.07	+35.8 (7.1)	45.5	67.8	122	62.0	96.4	170	29.3
**ND‐Tyr‐Lys**	0.07	+33.2 (7.1)	52.3	76.4	128	58.0	94.5	171	56.4

^a)^ Particle size distributions are given as volume distribution (D_v_ (10), D_v_ (50), D_v_ (90)) measured by DLS in doubly distilled water (dd‐H_2_O).

^b)^ Particle size distribution measured by DLS in dd‐H_2_O/Dulbecco's Modified Eagle Medium (DMEM) 9:1 + 10% bovine serum albumin (BSA).

^c)^ The median particle size was determined by AFM.

Detailed analysis of the recorded DRIFT spectra of **ND‐Tyr‐Glu**, **ND‐Tyr‐Leu,** and **ND‐Tyr‐Lys** can be found in the Supporting Information. To further prove the successful cleavage of the fmoc protection group, a colorimetric assay^[^ ^43^ ^]^ based on the Kaiser test was performed with all dipeptide‐functionalized NDs before and after fmoc deprotection. The results (see Figure S38) confirm the existence of the primary amino groups of the dipeptides.

The surface loadings calculated from TGA (Table 1) revealed a comparable coverage for **ND‐Tyr‐Glu**, **ND‐Tyr‐Leu,** and **ND‐Tyr‐Lys** but are still lower than for the previous material **ND‐N_3_**. This finding can be explained by cleavage of azide linker molecules by trifluoroacetic acid, which were not directly attached to the ND surface by C─C bond formation but to oxygen‐containing surface groups.

All three dipeptide functionalized ND particles demonstrate particle size distributions below 150 nm measured as hydrodynamic diameter by dynamic light scattering (DLS). As the hydrodynamic diameter is typically larger than the actual particle diameter due to the formation of a shell of water molecules, particle sizes were additionally determined by atomic force microscopy (AFM) (see Figure S36). The largest particle sizes were found for **ND‐Tyr‐Lys**, while **ND‐Tyr‐Glu** presents a significantly smaller average diameter. Generally, cellular uptake should be achievable with each of the three dipeptide ND conjugates, given that their particle sizes do not surpass 200 nm, which is described as upper size limit for energy‐dependent endocytosis.^[^ ^44^ ^]^

### Investigating Colloidal Stability of Functionalized NDs at Varying pH and in Biologically Relevant Media

Carrying out a pH dependent titration provides important insights into the particular protonation states of the immobilized dipeptides and allows predictions of the particle behavior in biological environments. The negative and positive charges of the surface functionalization simultaneously stabilize the particles in solution and prevent agglomeration. This can be seen from particle size versus pH titration measurements (see Figure S37), where all dipeptide‐functionalized NDs exhibit hydrodynamic diameters (Z average) of 100–200 nm and therefore colloidal stability from pH 3–9. For higher or lower pH values, unfunctionalized **mND** as well as the precursor **ND‐N_3_** formed large agglomerates in comparison to **ND‐Tyr‐Glu**, **ND‐Tyr‐Leu,** and **ND‐Tyr‐Lys**, which clearly show a reduced agglomeration tendency.

**Figure 1:**
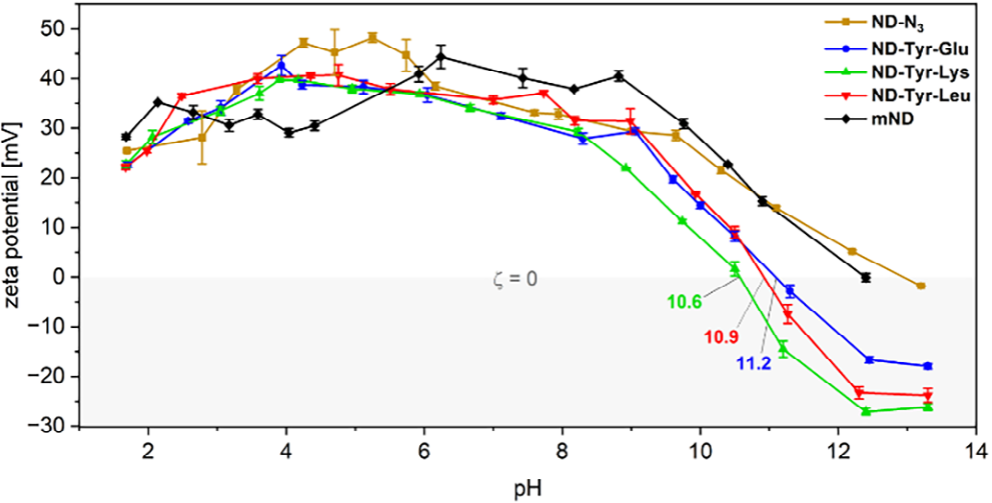
Zeta potential versus pH titration studies of dipeptide‐functionalized NDs, **ND‐N_3,_** and **mND**. Error bars show the deviation of triplicate measurements.

From zeta potential versus pH titration (Figure 1), it could be deduced that anionic and cationic groups of the dipeptide functionalization exist between pH values from 3 to 9 and effectively reinforce colloidal stability of ND particles through electrostatic interactions with molecules of the surrounding media. Furthermore, dipeptide‐functionalized NDs display a positive zeta potential in an acidic environment, which peaks between pH of 4 and 5.

Moving toward basic pH values, the zeta potential decreases continuously until it reaches zero around pH 11, marking the inversion of surface polarity of each individual ND conjugate. At strongly basic conditions, the zeta potential changes to highly negative values, which was not observed in case of the unfunctionalized **mND** or **ND‐N_3_**. At low pH, the dipeptide surface functionalization carries protonated carboxyl groups and positively charged amino groups that further contribute to the intrinsic positive zeta potential of the starting material **mND**. Increasing pH values cause deprotonation of the carboxyl groups and previously positively charged amino groups resulting in an overall negative zeta potential for all dipeptide‐functionalized NDs. Similar observations were found for cysteine‐functionalized silica nanoparticles.^[^ ^31^ ^]^

In order to further explore the colloidal stability of the synthesized particles, the evolution of the particle size in different serum protein‐containing media was monitored over a period of four days (Figure S40A–D). While the hydrodynamic diameters of all dipeptide‐functionalized NDs as well as the precursors **mND** and **ND‐N_3_** show comparable size ranges between 60 and 110 nm in water (Figure S40A), the size of **mND** and **ND‐N_3_** is considerably increasing when exposed to Dulbecco's modified Eagle medium (DMEM) or in the presence of serum protein‐containing media (newborn calf serum (NBCS), bovine serum albumin (BSA)), assumingly as a result of protein adsorption. In contrast, **ND‐Tyr‐Glu**, **ND‐Tyr‐Leu**, and **ND‐Tyr‐Lys** formed very stable colloidal dispersions in all tested media with particle sizes smaller than 200 nm. Out of all dipeptide‐functionalized NDs, **ND‐Tyr‐Glu** exhibited by far the smallest and most constant particle sizes. This can be attributed to the negative charges of the particle surface.

### Cytotoxicity Assay of Functionalized NDs Against Human Oral and Lung Cells

**Figure 2:**
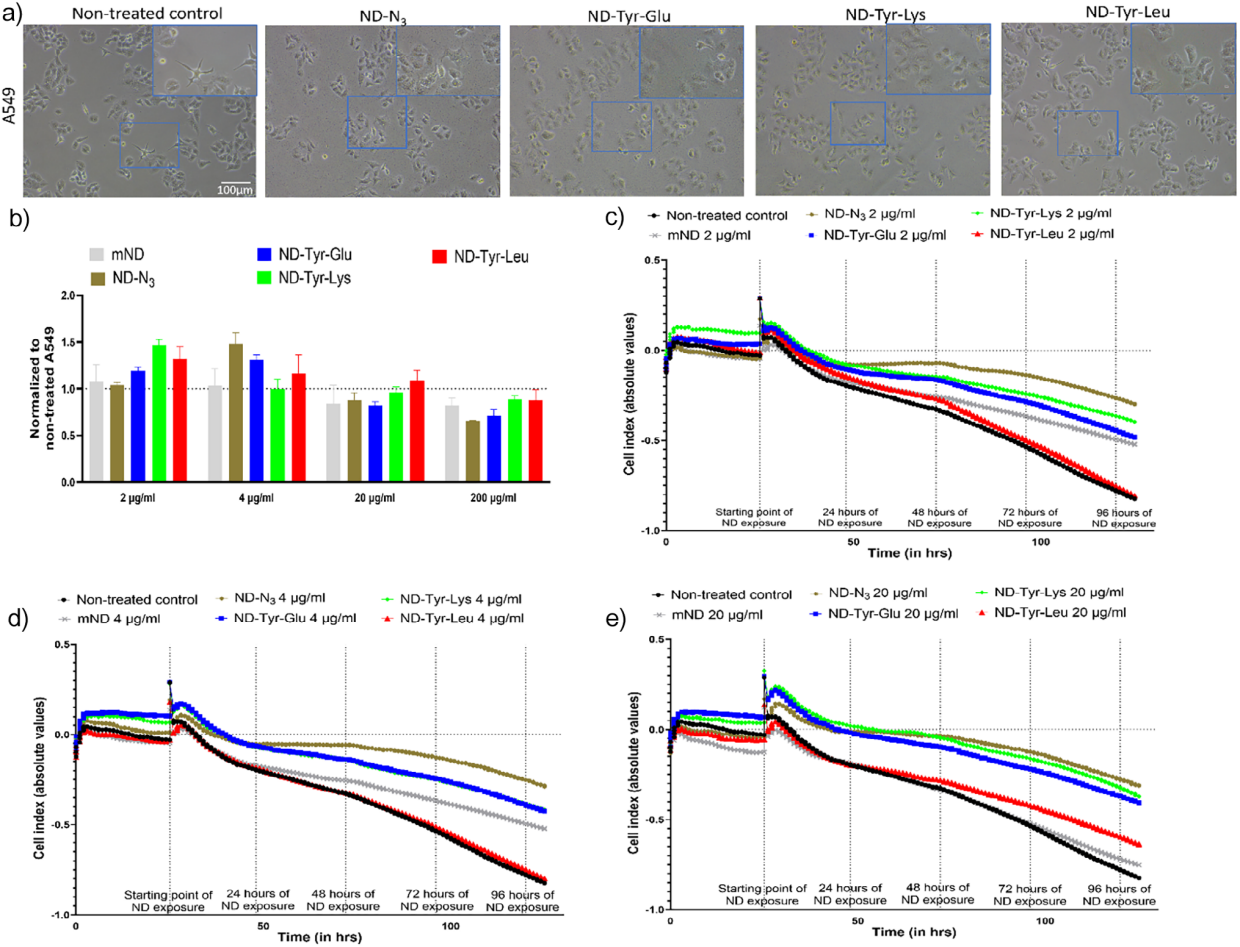
a) Phase contrast images showing cell morphology of A549 following exposure to different ND conjugates at 4 µg mL^−1^ concentration. Scale bar = 100 µm. b) Cellular viability assay investigating the effect of biologically relevant concentrations of different ND conjugates on A549 cell proliferation by manual cell counting. The data representing the ratio of living cells 48 h after ND treatment is normalized to untreated A549 and depicted as mean ± standard error over mean (SEM) of two biological repeats with each biological repeat consisting of three technical replicates. c)–e): Cytotoxicity of different ND conjugates at concentrations of c) 2 µg mL^−1^, d) 4 µg mL^−1^ and e) 20 µg mL^−1^ toward A549 was measured by assessing cell impedance using xCelligence system. The data presented is of two biological repeats with each repeat consisting of three technical replicates.

The cytotoxic effect and cellular uptake of functionalized nanoparticles was evaluated on oral epithelial cells (CaLH3), oral fibroblasts (NOF), and lung epithelial cells (A549). This was done in order to understand their effects on the major cell types in the upper aerodigestive tract for a potential oral delivery mode of administration. As depicted in the phase contrast images, agglomerates of the functionalized NDs could be seen inside treated cells (Figures 2a and S46A,B). It was found that the cellular morphology was similar to non‐treated controls when exposed to different concentrations of all functionalized NDs suggesting biocompatibility. Cellular toxicity assays based on manual cell count for all three cell lines were performed over a range of concentrations (2 to 200 µg mL^−1^) for all NDs 48 h after treatment (Figure 2b). Manual counting of cell viability was required because conventional colorimetric assays (MTT, WST‐1, Resazurin) were compromised by absorption/fluorescence originating from physisorbed dye molecules on the ND surface (addition of dye to NDs without cells led to false positive results).

Analysis of the obtained data generally presented a dose‐dependent decrease in cell viability for all tested NDs while going from the lowest to the highest ND concentration. **mND** did not induce any obvious cytotoxic effects on A549 when compared to its non‐treated controls, corroborating previous studies.^[^ ^45^ ^]^ In fact, at low concentrations, all functionalized NDs increased proliferation of A549. These observations were also confirmed on A549 cells with real‐time cell analysis using the impedance‐based xCELLigence system, which showed similar trends at the same concentrations (Figure 2c–e).

Further, toxicity analysis of azide functionalized **ND‐N_3_** revealed no significant differences in cell viability for all tested concentrations when compared to **mND**. Of note, at highest tested concentration of 200 µg mL^−1^, **ND‐N_3_** was more toxic than **mND**. When compared to **ND‐N_3_** at biologically relevant doses for all the cell lines, none of the dipeptide functionalized NDs were cytotoxic suggesting excellent biocompatibility. However, comparing the cytotoxic effects of dipeptide functionalized NDs among themselves on A549 cells at the same concentration of 200 µg mL^−1^, **ND‐Tyr‐Glu** (proportion of live cells to non‐treated controls: 70.9% ± 7.0%) had the highest toxicity of 29.1% (proportion of dead cells after 48 h treatment with respective ND conjugate) followed by **ND‐Tyr‐Leu** (11.5%, proportion of live cells: 88.5% ± 11%) and then **ND‐Tyr‐Lys** (10.9%, proportion of live cells: 89.1% ± 4.5%) (Figure 2b). Although increased toxicity to CaLH3 cells was observed with increased ND concentration, at the highest concentration of 200 µg mL^−1^, **ND‐Tyr‐Glu** (75.5% ± 6.3%) demonstrated again the highest toxicity (24.5%) (see Figure S46C). Similarly, **ND‐Tyr‐Glu** (58.0% ± 2.8%) were most toxic (42.0%) to oral fibroblast cells (NOF) followed by **ND‐Tyr‐Leu** (36.0%, proportion of live cells: 64.0% ± 2.8%) and **ND‐Tyr‐Lys** (30.0%, proportion of live cells: 70.0% ± 10.0%) at 200 µg mL^−1^ (Figure S46D).

Comparison only among the dipeptide functionalized NDs at high concentrations indicates, that **ND‐Tyr‐Glu** showed reduced biocompatibility across all tested cell lines. This observation is intriguing as the glutamic acid residue of **ND‐Tyr‐Glu** appears to have a different biological impact compared to leucine in **ND‐Tyr‐Leu** or lysine in **ND‐Tyr‐Lys**.

As the glutamic acid moiety is known to bind to several intracellular proteins and is involved in various metabolic pathways, it is necessary to further investigate the role of glutamic acid surface functionalizations in vitro in more detail.

At biologically relevant doses, **ND‐Tyr‐Leu** and **ND‐Tyr‐Lys** show high cytocompatibility. Consequently, the choice of amino acid as surface functionalization to manipulate the bioactivity of nanoparticles is crucial. This finding was corroborated in a study on selenium nanoparticles modified by adsorption of different amino acids, which were employed to enhance the anticancer potency of the nanoparticles.^[^ ^46^ ^]^ However, the biological effect caused by an amino acid or peptide surface functionalization is always influenced not only by the immobilized molecules themselves but also by their binding mode to the nanoparticle.

### Desquamation and Penetration of Different NDs in 3D Organotypic Oral Mucosa

The oral mucosa is a stratified epithelial tissue thus having different permeability and barrier function to cells grown in 2D monoculture.^[^ ^47^ ^]^ Homoeostasis of stratified epithelial tissue including oral mucosa depends on the balance of basal cell proliferation and cell loss at the surface, a process called desquamation. This desquamation is also responsible for elimination of potentially toxic agents.^[^ ^48^ ^]^ In that regard, a 3D organotypic model (OT) consisting of oral epithelial cells (CaLH3) and normal oral fibroblasts (NOF) was constructed to study desquamation and penetration of functionalized NDs aiming to understand the behavior of functionalized NDs in a 3D, tissue‐like environment. Compared to non‐treated control, **ND‐N_3_**, **ND‐Tyr‐Lys,** and **ND‐Tyr‐Leu** showed almost no desquamation, however, desquamation was significant when 3D OTs were treated with **ND‐Tyr‐Glu** (blue arrows, Figures S47A and 3a). This was also reflected in the total epithelial thickness of 3D OTs (*p* = 0.007, Figure S47B) and complements the data obtained from the cell viability assays in 2D cell cultures. Taken together our data suggest that **ND‐Tyr‐Glu** exhibits higher toxicity which may lead to prominent desquamation in 3D OTs.

**Figure 3:**
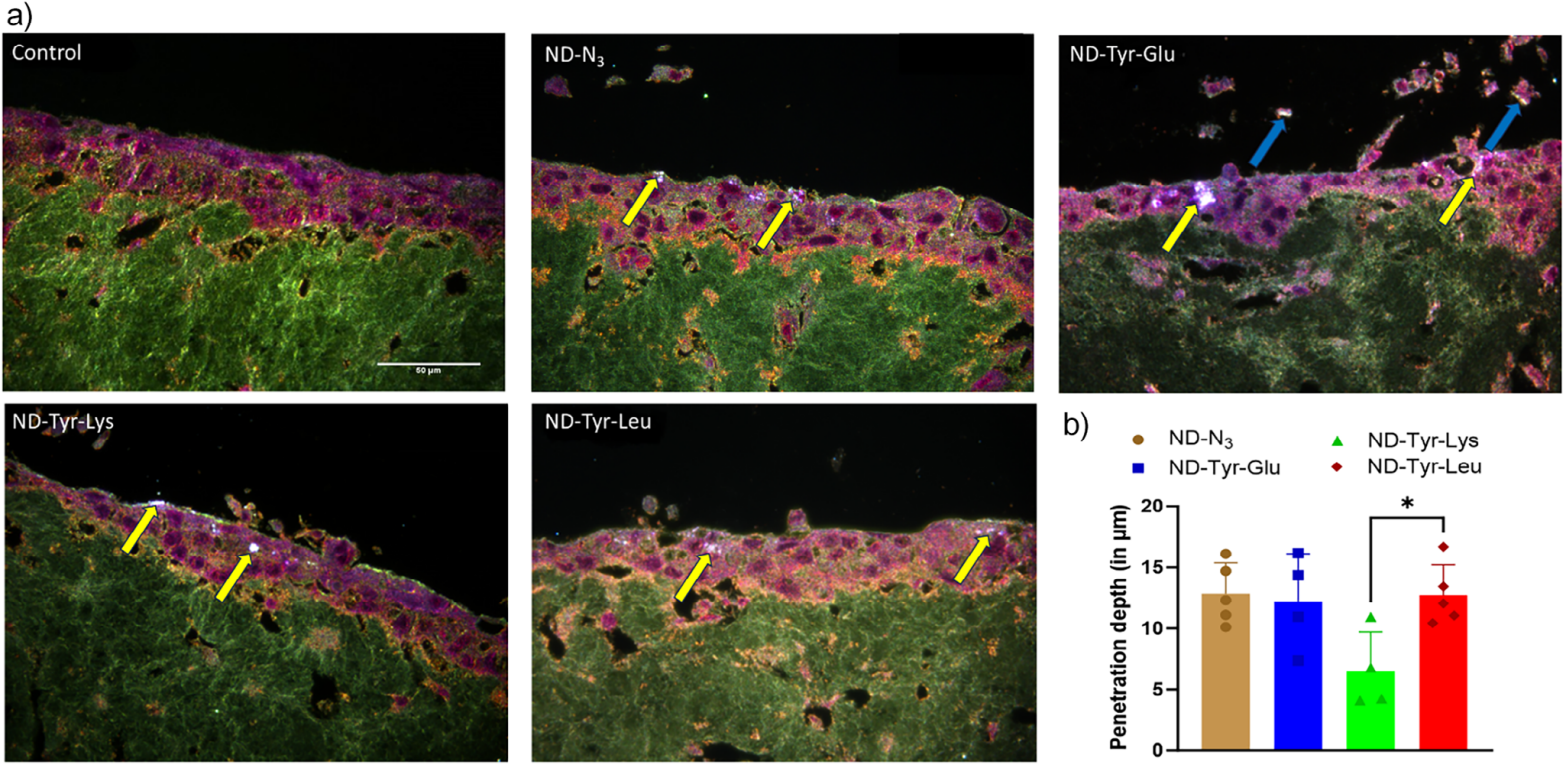
a) Representative ultrahigh resolution images of 3D OTs nonexposed controls and exposed to **ND‐N_3_**, **ND‐Tyr‐Glu**, **ND‐Tyr‐Lys,** and **ND‐Tyr‐Leu** taken on Cytoviva. Scale bar = 50 µm. b): Penetration depth of different NDs in the exposed 3D OTs. The data presented is mean ± SEM of measurements made on five images from each 3D OT. *p = 0.045.

To investigate the penetration depth of dipeptide functionalized NDs, ultrahigh‐resolution microscopy imaging (URI) was employed. All functionalized NDs penetrated the stratified oral mucosa with **ND‐Tyr‐Leu** and **ND‐Tyr‐Glu** exhibiting similar penetration depth as compared to **ND‐N_3_** (yellow arrows, Figure 3a). On the other hand, the penetration depth of **ND‐Tyr‐Lys** was significantly lower than **ND‐Tyr‐Leu** (*p* = 0.045).

As it can be seen in the case of **ND‐Tyr‐Glu** treated OT in Figure 3a, some of the cells lost from the surface contained ND particles (blue arrows) confirming that desquamation is one of the modalities with which stratified epithelial cells remove potentially harmful agents. However, since desquamation and thinning of the epithelial layer was detected neither for **ND‐Tyr‐Lys** nor **ND‐Tyr‐Leu**, the respective dipeptide functionalization of both ND conjugates can be considered as nontoxic in the employed 3D organotypic models.

### Uptake of Functionalized NDs in Human Lung Epithelial and Oral Fibroblasts

**Figure 4:**
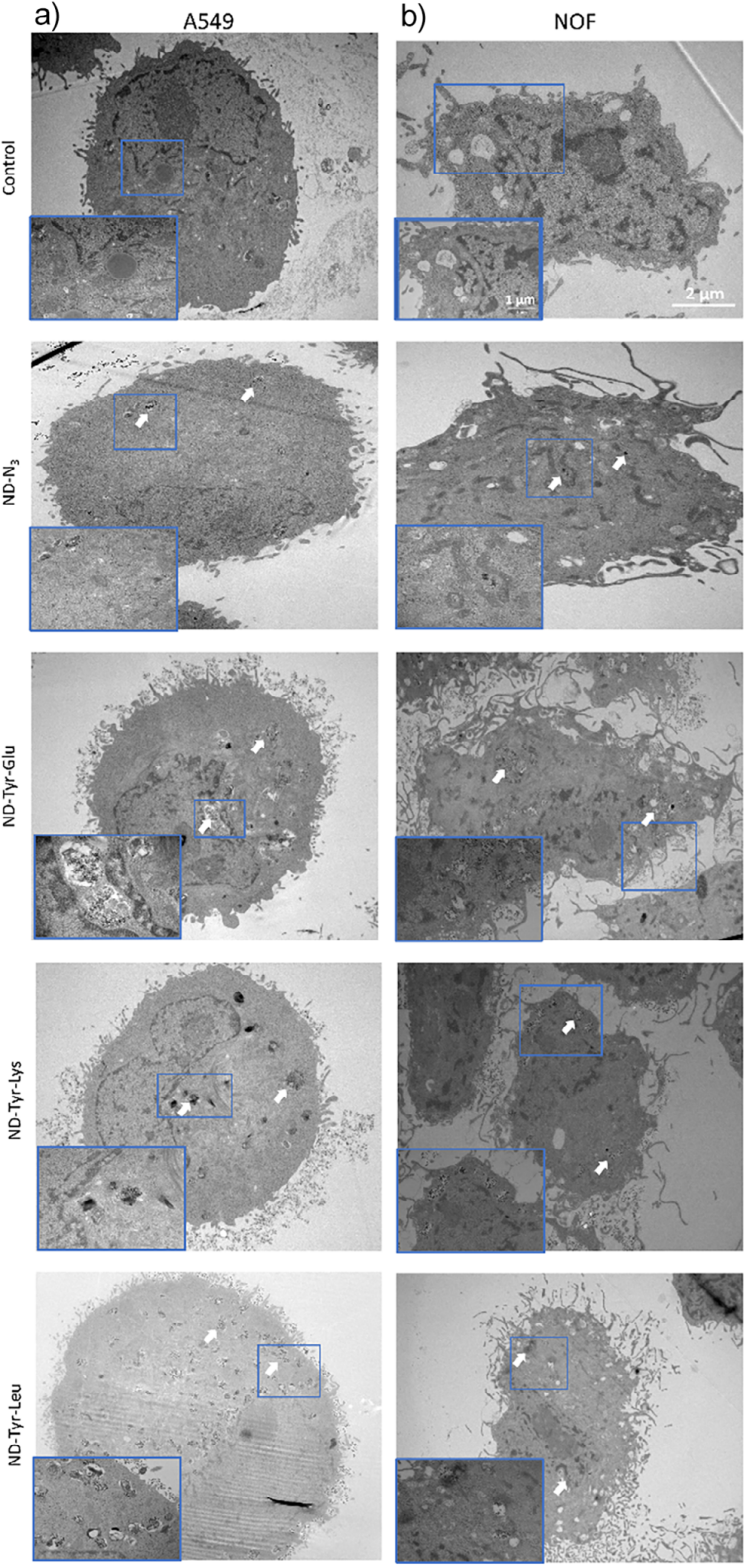
Uptake of different ND conjugates by a) A549 and b) NOF cells. Scale bar = 2 µm. Inset in each image showing internalized NDs, scale bar = 1 µm.

**Figure 5:**
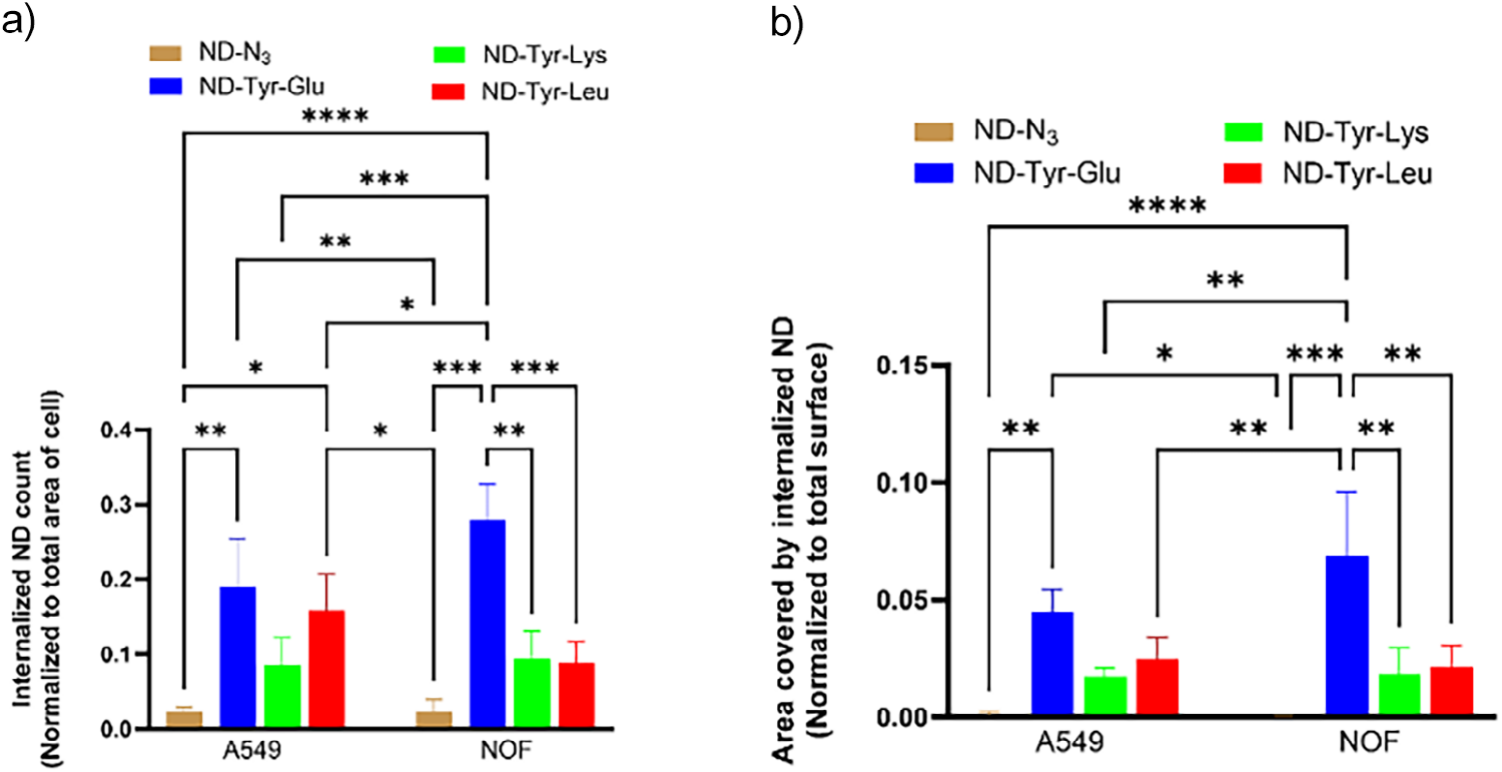
a) Graph showing count of internalized NDs per total area of A549 and NOF cells. b) Area covered by each internalized ND conjugate normalized to area of A549 and NOF cells.

Previous studies using transmission electron microscopy (TEM) have shown that uptake of NDs in A549 cells takes place via macropinocytosis and clathrin‐mediated endocytosis.^[^ ^49^ ^]^ In our study, TEM was used to confirm subcellular localization of the dipeptide functionalized NDs after an incubation time of 48 h. As seen in TEM images, all the functionalized NDs were localized in the cytoplasm but not in the nucleus of both A549 (Figure 4a) and NOF cells (Figure 4b). Functionalized NDs were also found on the surface of cells. In the cytoplasm, NDs could be observed scattered around as well as in the endocytic vesicles in clusters (Figure 4, white arrows). The area covered by the ND clusters in the cell was considered to be indicative of higher uptake. In general, all dipeptide functionalized NDs exhibited a significantly higher number of internalized particles compared to **ND‐N_3_**, demonstrating enhanced uptake for this type of particles (Figure 5). The area was highest for **ND‐Tyr‐Glu** both in A549 and NOF cells followed by **ND‐Tyr‐Leu** and **ND‐Tyr‐Lys**. Moreover, in NOF cells the area covered by **ND‐Tyr‐Glu** was significantly higher than by both **ND‐Tyr‐Leu** (*p* = 0.04) and **ND‐Tyr‐Lys** (*p* = 0.03) (Figure 5b). Similarly, the **ND‐Tyr‐Glu** count was significantly higher in A549 cells than both **ND‐Tyr‐Leu** (*p* = 0.002) and **ND‐Tyr‐Lys** (*p* = 0.002) (Figure 5a). This is most likely related to the comparably small particle size and high colloidal stability of **ND‐Tyr‐Glu** observed in the earlier described stability test. Small, stable particles are internalized by the cells more efficiently. For **ND‐Tyr‐Leu** and **ND‐Tyr‐Lys**, larger particle sizes as measured by DLS and AFM evidentially resulted in a reduced uptake. It is important to note that the endocytic NDs were not degraded within the observed timeframe and existed without inducing cellular damage. However, owing to the different particle sizes of dipeptide functionalized NDs and the consequent differences in cellular uptake, the observed toxicity of **ND‐Tyr‐Glu** might be correlated with the higher number of internalized particles for particle sizes below 100 nm as it is shown in literature.^[^ ^19^ ^]^ On the other hand, due to the different dipeptide functionalization, there could be fundamental differences in biological activity and/or degradation products, which could also influence internalization and toxicity. To determine the exact cause of the occurring toxicity of **ND‐Tyr‐Glu**, further investigations on a molecular level would be required.

In addition to the evaluated extent of ND internalization, conclusions on the uptake pathway can be drawn from the captured TEM images as well. For NDs, it has been reported that shape, size, and surface charge of the particles dictate the pathway through which NDs enter mammalian cells as well as their subsequent cytosolic availability.^[^ ^50^ ^]^ In the case of dipeptide functionalized NDs, filopodia extensions surrounded by particles can be observed possibly assisting uptake (see Figure S48). This process is known as macropinocytosis, where cell membrane extensions (membrane ruffles) are generated upon stimulation to engulf external fluid including particles.^[^ ^51^ ^]^ Macropinocytosis was also identified previously as uptake mechanism for carboxylated NDs with a particle size of 100 nm and lysine functionalized NDs.^[^ ^23^, ^49^ ^]^

## Conclusion

In summary, we report here on a general method to synthetize highly biologically suitable ND particles, which are nontoxic at biologically relevant doses, easily internalized in cells, exhibit no tendency of agglomeration in biofluids and show sustained dispersibility even in physiological media. This is achieved by a zwitterionic surface functionalization based on differently charged dipeptides using procedures from “click” chemistry and solid phase peptide synthesis. Our results show that zwitterionic amino acid functionalizations enable control over behavior and fate of nanoparticles in biological systems, which is a crucial prerequisite for applications like targeted drug delivery, biosensing, or bioimaging. We successfully immobilized three different dipeptides covalently on fully deagglomerated detonation ND and characterized the physicochemical properties of the respective ND conjugates. Compared to unfunctionalized **mND**, all three attached zwitterionic dipeptides promote colloidal stability and retention of particle size over a broad pH range and in different protein‐containing media. Among them, **ND‐Tyr‐Glu** exhibits the smallest particle size distribution and highest colloidal stability. When exposed to normal and malignant cells from the upper aerodigestive tract in 2D and 3D cell cultures, all dipeptide functionalized NDs present a nontoxic behavior in biologically relevant doses and were internalized predominantly by macropinocytosis. For higher concentrations, cell viability decreased in a concentration‐dependent manner, with **ND‐Tyr‐Glu** showing a higher extent of toxicity. Furthermore, desquamation in 3D organotypic models was observed exclusively for **ND‐Tyr‐Glu**, while **ND‐Tyr‐Leu** and **ND‐Tyr‐Lys** did not induce any toxicity or morphological changes within the conducted biological tests. Similarly, a dipeptide‐dependent uptake into A549 and NOF cells was discovered. As the functionalization of the three investigated ND conjugates only differs in the incorporated second amino acid, the glutamic acid residue of **ND‐Tyr‐Glu** could be responsible for the detected effects. To conclude, comparison of three different dipeptide ND functionalizations lead to the identification of two systems (**ND‐Tyr‐Leu** and **ND‐Tyr‐Lys**) that meet several biologically relevant criteria underlining the importance of a thorough and precise selection of surface modification while providing a promising surface architecture for the incorporation of additional functionalities. In the future, we will study the use of these ND conjugates for biomedical applications and investigate the specific role of glutamic acid as a surface moiety.

## Conflict of Interests

The authors declare no conflict of interest.

## Supporting information



Supporting Information

## Data Availability

The data that support the findings of this study are available from the corresponding author upon reasonable request.
