# Supplementary material for: Zwitterionic Dipeptide Surface Functionalization of Detonation Nanodiamond for Enhanced Control in Biological Environments
Source: Angew Chem Int Ed Engl. 2025 May 19;64(25):e202501202. doi: 10.1002/anie.202501202 (PMC12171329; doi:10.1002/anie.202501202)
Supplement: Supplementary file 1 — Supporting Information [file ANIE-64-e202501202-s001.pdf]

Supporting Information

Zwitterionic Dipeptide Surface Functionalization of Detonation Nanodiamond for Enhanced Control in Biological Environments

by Elisabeth Mayerhoefer[1], Himalaya Parajuli[2], Mihaela-Roxana Cimpan[3], Daniela Elena Costea[2,4], Harsh Nitin Dongre[2,4], and Anke Krueger[*,1,5]

[1]*Institute of Organic Chemistry, University of Stuttgart, Pfaffenwaldring 55, 70569 Stuttgart, Germany*

[2]*The Gade Laboratory for Pathology and Centre for Cancer Biomarkers (CCBIO), Department of Clinical Medicine, University of Bergen, Jonas Lies vei 87, 5021 Bergen, Norway*

[3]*Department of Clinical Dentistry, University of Bergen, Årstadveien 19, 5009 Bergen, Norway*

[4]*Department of Pathology, Haukeland University Hospital, 5009 Bergen, Norway*

[5]*Center for Integrated Quantum Science and Technology, University Stuttgart, 70569 Stuttgart, Germany*

Table of Contents

1. **Experimental Procedures**
   1.1 Materials and methods for organic synthesis
   1.2 Characterization data of organic compounds
   1.3 Materials and methods for ND characterization
   1.4 Particle syntheses and characterization
   1.5 Methods for *in vitro* experiments
2. **Results and Discussion**
   2.1 Analysis of DRIFT and FTIR spectra of dipeptide functionalized NDs
   2.2 Particle size distribution of **ND-N$_3$**, **ND-Tyr-Glu**, **ND-Tyr-Leu** and **ND-Tyr-Lys** as aqueous dispersion measured by dynamic light scattering
   2.3 Particle size of **ND-Tyr-Glu**, **ND-Tyr-Leu** and **ND-Tyr-Lys** measured by AFM
   2.4 Particle size vs. pH titration of **mND**, **ND-N$_3$**, **ND-Tyr-Glu**, **ND-Tyr-Leu** and **ND-Tyr-Lys**
   2.5 Colorimetric assay (Kaiser test)
   2.6 Thermogravimetric analysis
   2.7 Colloidal stability of **ND-N$_3$** and dipeptide functionalized NDs in different media
   2.8 Cell morphology and proliferation of CaLH3 and NOF cells after ND treatment
   2.9 Hematoxylin-eosin stained 3D OT after exposure to different ND conjugates
   2.10 Ultrastructural localization of **ND-Tyr-Glu**
3. **References**
4. **Author Contributions**

1. Experimental Procedures

1.1 Materials and methods for organic synthesis

Unless otherwise stated, all chemicals used for synthesis were reagent grade purchased from commercial suppliers and used without further purification. The amino acid *N*-[(9*H*-fluoren-9-ylmethoxy)carbonyl]-*O*-2-propyn-1-yl-L-tyrosine was purchased from *Iris Biotech*, di-*tert*-butyl-L-glutamate hydrochloride, *tert*-butyl-L-leucinate hydrochloride and *tert*-butyl-N^6-(((9*H*-fluoren-9-yl)methoxy)carbonyl)-L-lysinate hydrochloride from *BLD Pharm*. All solvents were purified by distillation prior to use and dried according to standard procedures when necessary. Reactions involving air- and/or moisture-sensitive reagents were conducted in dry glassware under nitrogen atmosphere. If not particularly mentioned, all synthetic procedures were performed under ambient conditions.

Nuclear magnetic resonance spectra (^{1}H- and ^{13}C-NMR) were recorded with a *Bruker Avance* 400 FT-NMR spectrometer at 400 MHz (^{1}H) or 100 MHz (^{13}C). The chemical shift δ is reported in ppm using the residual proton signal of the deuterated solvent for internal calibration. For literature-unknown compounds, two-dimensional NMR spectra (COSY, HMBC, HSQC) were measured to support signal assignment.

FT-IR spectra were obtained with a *Jasco* FT-IR-410 spectrometer equipped with an attenuated total reflection (ATR) unit for organic compounds.

UV/Vis spectra of all dipeptides were measured in HPLC grade acetonitrile (c(dipeptide) = $1 \cdot 10^{-3}$ mol·L^{-1}) with a *Jasco* V-630 UV-Vis spectrometer using 1.0 cm quartz cuvettes.

Electrospray ionization (ESI) mass spectrometry was performed with a *Bruker Daltonics* microTOF focus in positive mode.

Elemental analysis (CHNS analysis) was performed using a *HEKAtech* Euro EA CHNS Elemental Analyser. The samples were prepared with vanadium pentoxide as combustion aid.

1.2 Characterization data of organic compounds

4-(2-Azidoethyl)phenylamine (**8**)[1]

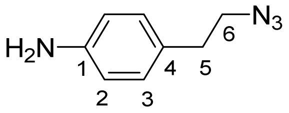

8

1.50 g (10.9 mmol, 1.00 eq) of 4-aminophenethyl alcohol, 2.86 g of triphenylphosphine (10.9 mmol, 1.00 eq), 840 mg of sodium azide (12.9 mmol, 1.18 eq) and 3.63 g of tetrabromomethane (10.9 mmol, 1.00 eq) were dissolved in 40 mL of DMF and the mixture was stirred at 90 °C for 4 h. After cooling to room temperature, 30 mL of water were added and the mixture was stirred for another 10 min. Afterwards, the mixture was extracted with diethyl ether (5x, each 40 mL). The combined organic phases were washed with water (3x, each 30 mL), dried over Na_2SO_4 and the solvent was removed *in vacuo*. The crude product was purified by column chromatography (silica gel, cyclohexane/ethyl acetate, 1:2, v/v). After removing the solvent, the product was obtained as a yellow oil.

Yield: 1.04 g (6.41 mmol, 59 %). **R$_f$** 0.23 (cyclohexane/ethyl acetate, 2:1). **^{1}H-NMR** (400 MHz, $CDCl_3$): δ = 7.02 – 6.99 (m, 2H, H-3), 6.66 – 6.64 (m, 2H, H-2), 3.61 (s, 2H, NH_2) 3.43 (t, $^3J_{6,5}$ = 7.32 Hz, 2H, H-6), 2.79 (t, $^3J_{5,6}$ = 7.28 Hz, 2H, H-5) ppm. **^{13}C-NMR** (100 MHz, $CDCl_3$): δ = 145.2 (C_q, C-1), 129.7 (C_t, C-3), 128.0 (C_q, C-4), 115.5 (C_t, C-2), 52.9 (C_s, C-6), 34.6 (C_s, C-5) ppm. **FT-IR** (ATR): $\tilde{\nu}$ = 3442 (br, w, ν(N-H)), 3361 (br, w, ν(N-H)), 3217 (br, w, ν(N-H)), 3018 (w, ν(C-H)), 2927 (w, ν(C-H)), 2868 (w), 2092 (vs, $\nu(N_3)$), 1622 (m, ν(C=C$_{arom.}$)), 1516 (s, ν(C=C$_{arom.}$)), 1439 (w), 1346 (w), 1271 (m), 1180 (w), 1126 (w), 1036 (w), 899 (w), 819 (s, δ(C-H$_{arom.}$)) cm^{-1}. **HRMS** (ESI,+): found: 163.0976 for $C_8H_{11}N_4$ [M+H]$^+$; calc.: 163.0978 for [M+H]$^+$.

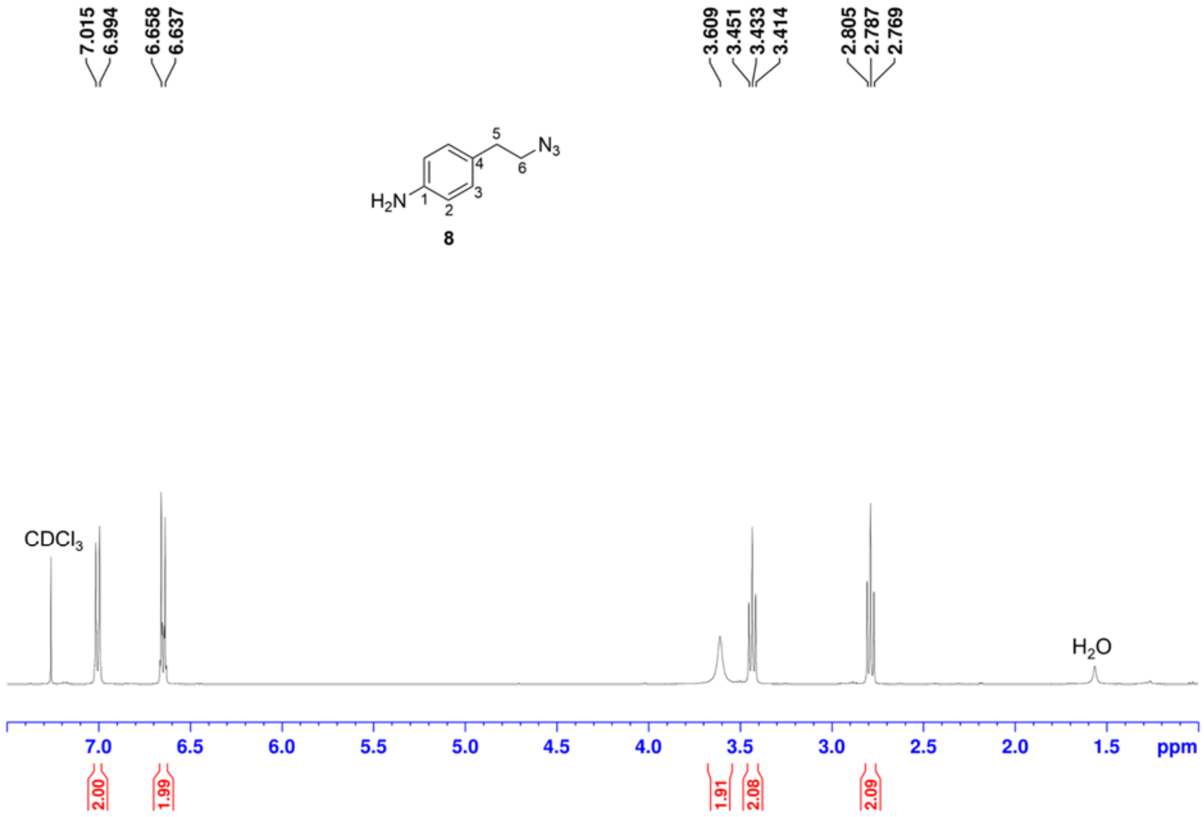

Figure S1: ^{1}H-NMR spectrum (400 MHz, $CDCl_3$) of 4-(2-azidoethyl)phenylamine (**8**).

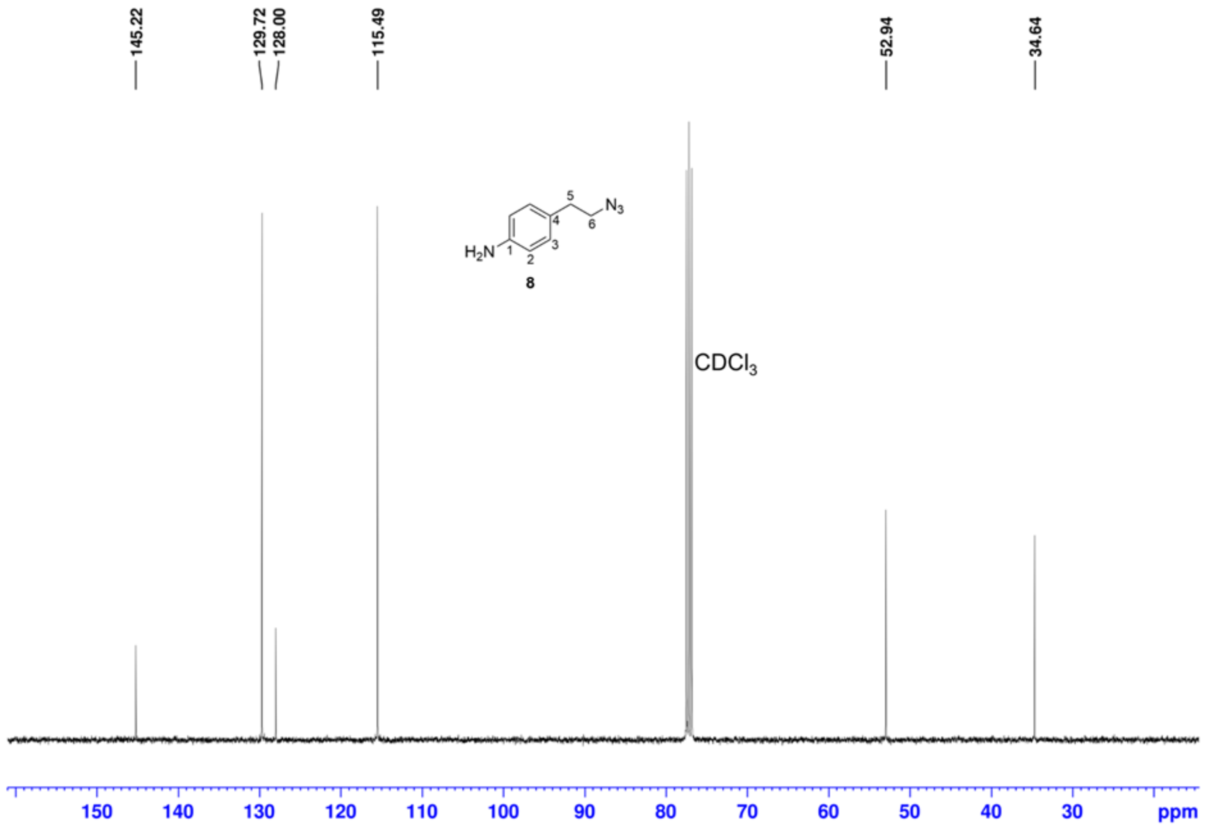

Figure S2: ^{13}C-NMR spectrum (101 MHz, $CDCl_3$) of 4-(2-azidoethyl)phenylamine (**8**).

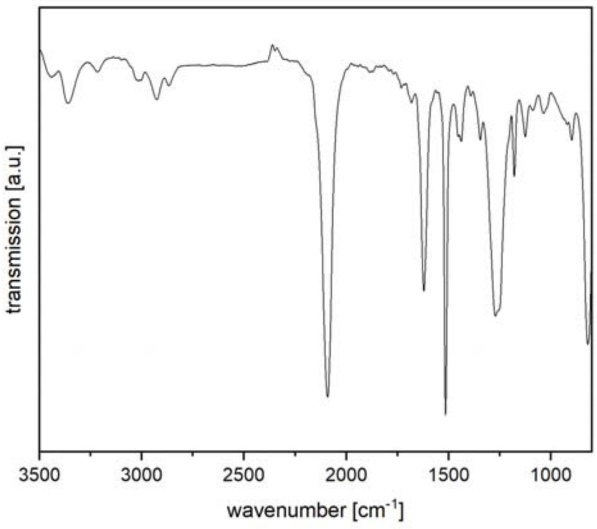

Figure S3: FT-IR (ATR) spectrum of 4-(2-azidoethyl)phenylamine (**8**).

General procedure for dipeptide synthesis

Under N_2 atmosphere, 100 mg of *N*-[(9*H*-fluoren-9-ylmethoxy)carbonyl]-*O*-2-propyn-1-yl-L-tyrosine **1** (0.23 mmol, 1.00 eq) were dissolved in 10 mL of dry DMF at 0 °C. Subsequently, 80.0 mg of 2-(1*H*-benzotriazole-1-yl)-1,1,3,3-tetramethylamonnium tetrafluoroborate (TBTU, 0.25 mmol, 1.10 eq) and 0.12 mL of *N*,*N*-diisopropylethylamine (DIPEA, 87.8 mg, 0.68 mmol, 3.00 eq) were added to the mixture. After stirring for 30 min at 0 °C, 1.00 eq of the respective second amino acid was added in 2 mL of dry DMF. The mixture was stirred for another 60 min at 0 °C, followed by 17 h stirring at room temperature. After completion of the reaction (TLC), the mixture was diluted with 10 mL of water and 2 mL of saturated NH_4Cl solution and then extracted with ethyl acetate (3x, each 20 mL). The combined organic phases were dried over $MgSO_4$ followed by removal of the solvent *in vacuo*. Purification of the crude product by column chromatography (silica gel, cyclohexane/ethyl acetate, 1:4, v/v) afforded the respective dipeptide.

Di-*tert*-butyl-((*S*)-2-((((9*H*-fluoren-9-yl)methoxy)carbonyl)amino)-3-(4-(prop-2-yn-1 yloxy)phenyl)propanoyl)-L-glutamate (**5**)

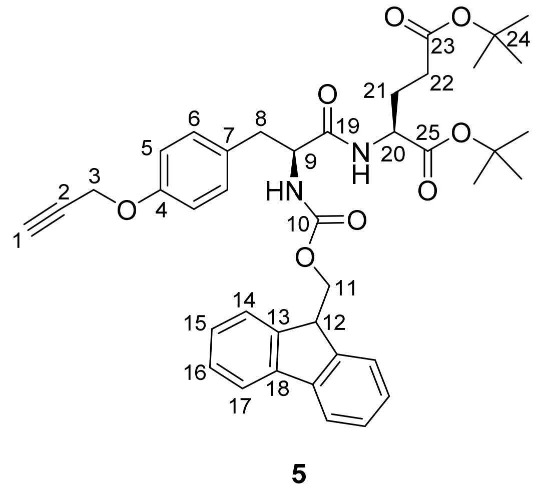

5

Dipeptide ≡—O-Tyr-Glu **5** was synthesized from 100 mg *N*-[(9*H*-fluoren-9-ylmethoxy)carbonyl]-*O*-2-propyn-1-yl-L-tyrosine **1** (0.23 mmol, 1.00 eq) and 58.7 mg di-*tert*-butyl-L-glutamate hydrochloride **2** (0.23 mmol, 1.00 eq) according to the reported general procedure and obtained as a colorless solid.

Yield: 136 mg (0.20 mmol, 88 %). **Mp.** 100 – 101 °C **R** R_f 0.69 (cyclohexane/ethyl acetate, 1:1). **^{1}H-NMR** (400 MHz, $CDCl_3$): δ = 7.77 (d, $^3J_{17,16}$ = 7.56 Hz, 2H, H-17), 7.58 – 7.54 (m, 2H, H-14), 7.42 – 7.39 (m, 2H, H-16), 7.34 – 7.30 (m, 2H, H-15), 7.10 – 7.08 (m, 2H, H-5), 6.89 – 6.87 (m, 2H, H-6), 6.53 – 6.51 (m, 1H, amide-*H*), 5.29 – 5.27 (m, 1H, NH_{Tyr}), 4.63 (d, $^4J_{3,1}$ =

2.16 Hz, 2H, H-3), 4.48 – 4.38 (m, 3H, H-9, H-20, H-11), 4.34 – 4.30 (m, 1H, H-11), 4.19 (t, $^{3}J_{12,11}$ = 6.78 Hz, 1H, H-12), 3.12 – 2.96 (m, 2H, H-8), 2.49 (t, $^{4}J_{1,3}$ = 2.36 Hz, 1H, H-1), 2.28 – 2.11 (m, 2H, H-22), 2.09 – 2.04 (m, 1H, H-21), 1.90 – 1.84 (m, 1H, H-21), 1.45 – 1.42 (m, 18H, H-26) ppm. **^{13}C-NMR** (100 MHz, $CDCl_3$): δ = 172.2 (C_q, C-23), 170.6 (C_q, C-19/25), 156.8 (C_q, C-4), 155.9 (C_q, C-10), 143.9 (C_q, C-13), 141.4 (C_q, C-18), 130.6 (C_t, C-5), 129.1 (C_q, C-7), 127.9 (C_t, C-16), 127.2 (C_t, C-15), 125.2 (C_t, C-14), 120.1 (C_t, C-17), 115.2 (C_t, C-6), 82.6 (C_q, C-24), 81.0 (C_q, C-24), 78.7 (C_q, C-2), 75.6 (C_t, C-1), 67.2 (C_s, C-11), 56.1 (C_t, C-9), 55.9 (C_s, C-3), 52.5 (C_t, C-20), 47.3 (C_t, C-12), 37.8 (C_s, C-8), 31.4 (C_s, C-21), 28.1 (C_p, C-26), 27.6 (C_s, C-21) ppm. **FT-IR** (ATR): $\tilde{\nu}$ = 3296 (w, ν(C≡C-H)), 2978 (w, ν(C-H)), 1728 (m, ν(C=O_{ester})), 1660 (m, ν(C=O), amide I), 1610 (w, ν(C-N), amide II), 1510 (m, ν(C=C_{arom})), 1448 (w), 1369 (m), 1238 (s, ν(C-O-C_{ester})), 1151 (s, δ(C-O_{ester})), 1117 (w), 1036 (m, ν(C-O_{ether})), 926 (w), 845 (m, δ(C-H_{arom})) cm^{-1}. **HRMS** (ESI,+): found: 705.31940 for $C_{40}H_{46}N_2NaO_8$ $[M+Na]^+$; calc.: 705.3152 for $[M+Na]^+$. **CHNS analysis**: found: C: 70.06 %, H: 6.90 %, N: 3.99 %; calc.: C: 70.36 %, H: 6.79 %, N: 4.10 %. **UV/Vis** (MeOH): λ_{max} = 206.5 nm.

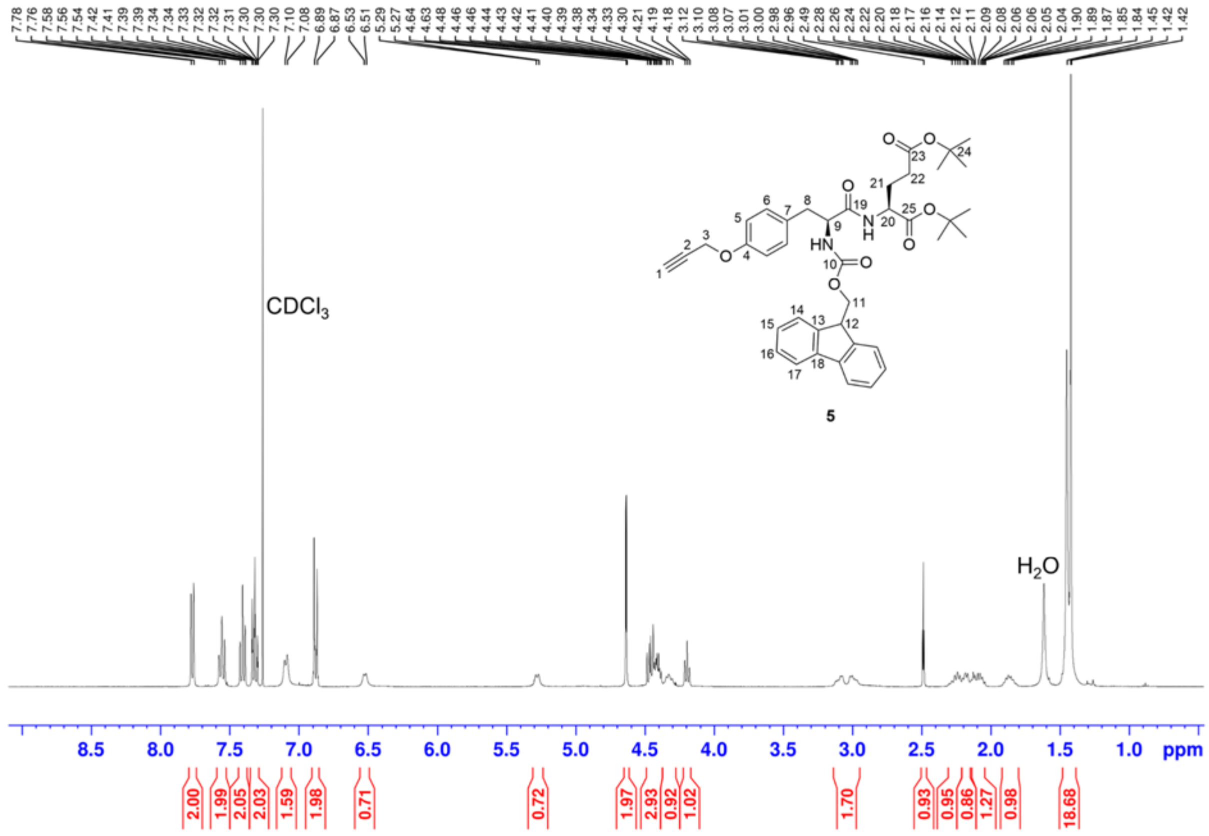

Figure S4: ^{1}H-NMR spectrum (400 MHz, $CDCl_3$) of dipeptide ≡—O-Tyr-Glu (**5**).

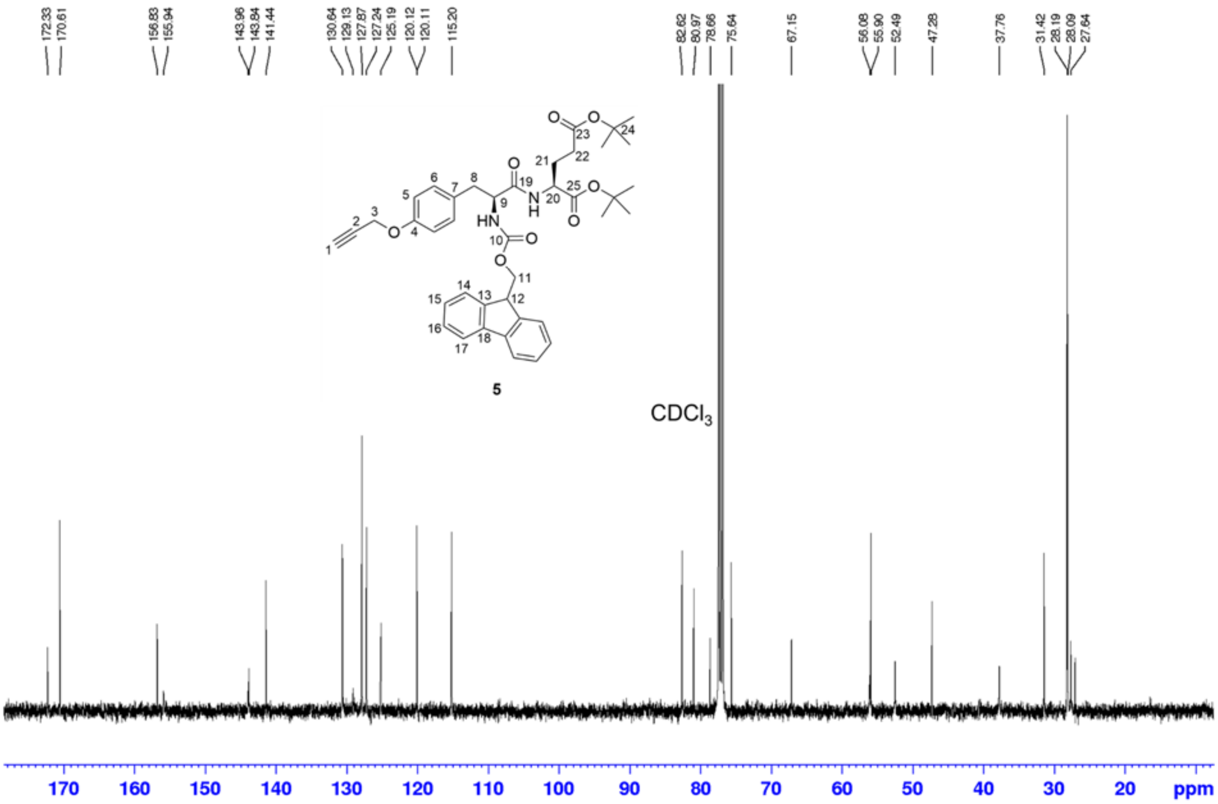

Figure S5: ^{13}C-NMR spectrum (101 MHz, $CDCl_3$) of dipeptide ≡—O-Tyr-Glu (**5**).

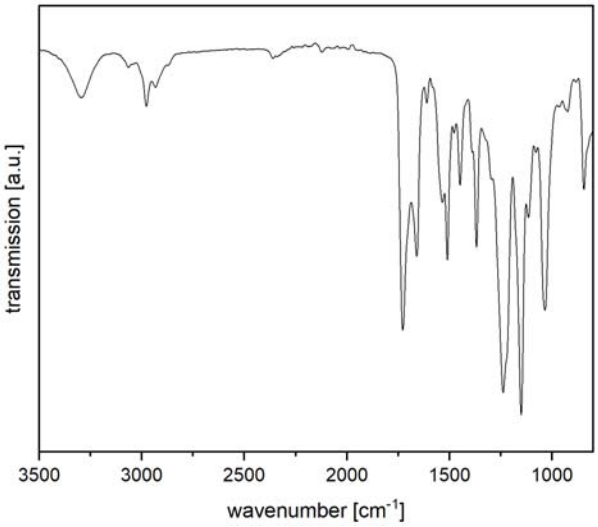

Figure S6: FT-IR (ATR) spectrum of dipeptide ≡—O-Tyr-Glu (**5**).

tert-Butyl-((*S*)-2-((((9*H*-fluoren-9-yl)methoxy)carbonyl)amino)-3-(4-(prop-2-yn-1-yloxy)phenyl)propanoyl)-L-leucinate (**6**)

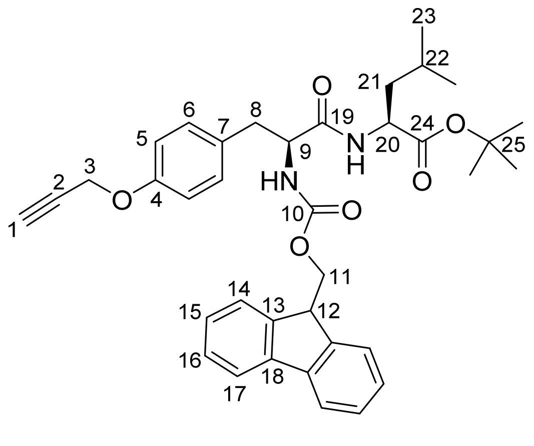

6

Dipeptide ≡─O-Tyr-Leu **6** was synthesized from 100 mg *N*-[(9*H*-fluoren-9-ylmethoxy)carbonyl]-*O*-2-propyn-1-yl-L-tyrosine **1** (0.23 mmol, 1.00 eq) and 50.7 mg *tert*-butyl-L-leucinate hydrochloride **3** (0.23 mmol, 1.00 eq) according to the reported general procedure and obtained as a colorless solid.

Yield: 129 mg (0.21 mmol, 93 %). **Mp.** 113 – 114 °C. **R$_f$** 0.72 (cyclohexane/ethyl acetate, 1:1). **^{1}H-NMR** (400 MHz, $CDCl_3$): δ = 7.77 (d, $^3J_{17,16}$ = 7.52 Hz, 2H, H-17), 7.57 – 7.54 (m, 2H, H-14), 7.43 – 7.39 (m, 2H, H-16), 7.34 – 7.30 (m, 2H, H-15), 7.11 – 7.10 (m, 2H, H-5), 6.89 – 6.87 (m, 2H, H-6), 6.13 – 6.11 (m, 1H, amide-*H*), 5.29 – 5.27 (m, 1H, NH_{Tyr}), 4.64 (d, $^4J_{3,1}$ = 2.16 Hz, 2H, H-3), 4.48 – 4.40 (m, 3H, H-9, H-20, H-11), 4.35 – 4.31 (m, 1H, H-11), 4.19 (t, $^3J_{12,11}$ = 6.78 Hz, 1H, H-12), 3.10 – 2.97 (m, 2H, H-8), 2.49 (t, $^4J_{1,3}$ = 2.36 Hz, 1H, H-1), 1.55 (m, 3H, H-22/21, (superposition with water traces)), 1.44 (s, 9H, H-26), 0.91 – 0.88 (m, 6H, H-23) ppm. **^{13}C-NMR** (100 MHz, $CDCl_3$): δ = 171.7 (C_q, C-19), 170.3 (C_q, C-24), 156.8 (C_q, C-4), 143.9 (C_q, C-13), 141.4 (C_q, C-18), 130.7 (C_t, C-5), 129.3 (C_q, C-7), 127.9 (C_t, C-16), 127.2 (C_t, C-15), 125.2 (C_t, C-14), 120.2 (C_t, C-17), 115.2 (C_t, C-6), 82.2 (C_q, C-25), 78.7 (C_t, C-2), 75.6 (C_t, C-1), 67.2 (C_s, C-11), 55.9 (C_t, C-9), 55.8 (C_s, C-3), 51.6 (C_t, C-20), 47.3 (C_t, C-12), 42.0 (C_s, C-21), 37.6 (C_s, C-8), 28.1 (C_p, C-26), 25.0 (C_t, C-22), 22.9 (C_p, C-23), 22.3 (C_p, C-23) ppm. **FT-IR (ATR)**: $\tilde{\nu}$ = 3292 (w, ν(C≡C-H)), 3066 (w, ν(C-H)), 2958 (w, ν(C-H)), 1732 (m, ν(C=O_{ester})), 1695 (m, ν(C=O), amide I), 1654 (s, ν(C-N), amide II), 1610 (w), 1541 (m), 1510 (m, ν(C=C_{arom})), 1448 (w), 1392 (w), 1369 (m), 1242 (s, ν(C-O-C_{ester})), 1147 (s, δ(C-O-C_{ester})), 1032 (s, ν(C-O-C_{ether})), 941 (w), 827 (m, δ(C-H_{arom})) cm^{-1}. **HRMS** (ESI,+): found: 633.29588 for $C_{37}H_{42}N_2NaO_6$ $[M+Na]^+$; calc.: 633.2941 for $[M+Na]^+$. **CHNS analysis**: found: C: 72.91 %, H: 6.88 %, N: 4.66 %; calc.: C: 72.76 %, H: 6.93 %, N: 4.59 %. **UV/Vis** (MeOH): λ_{max} = 205.5 nm.

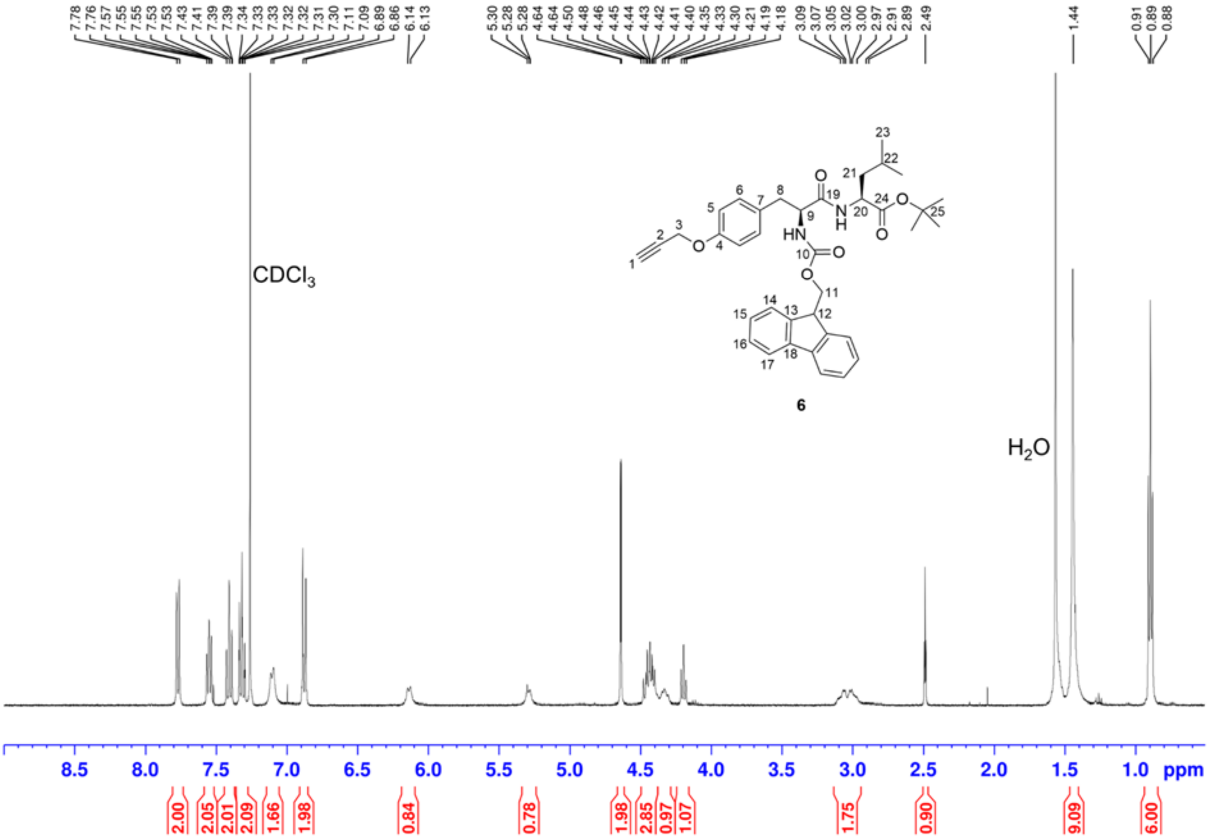

Figure S7: ^{1}H-NMR spectrum (400 MHz, $CDCl_3$) of dipeptide ≡─O-Tyr-Leu (**6**).

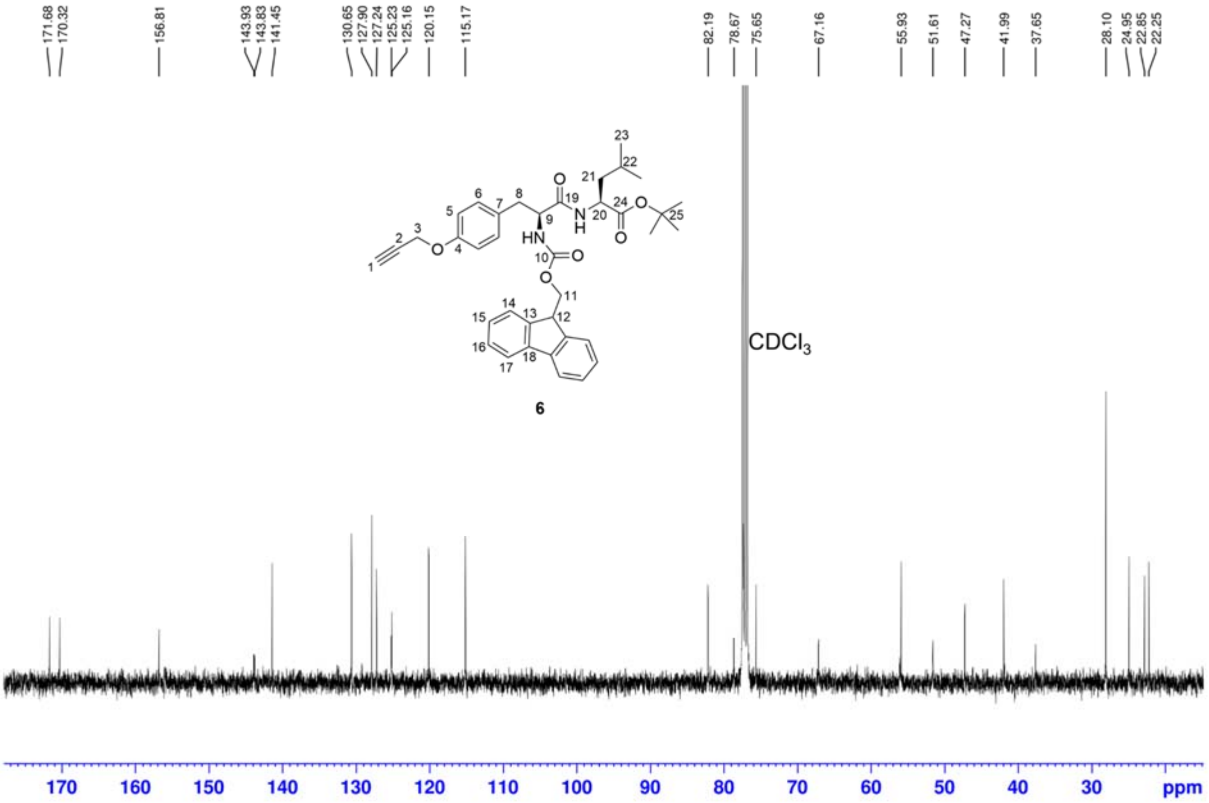

Figure S8: ^{13}C-NMR spectrum (101 MHz, $CDCl_3$) of dipeptide ≡─O-Tyr-Leu (**6**).

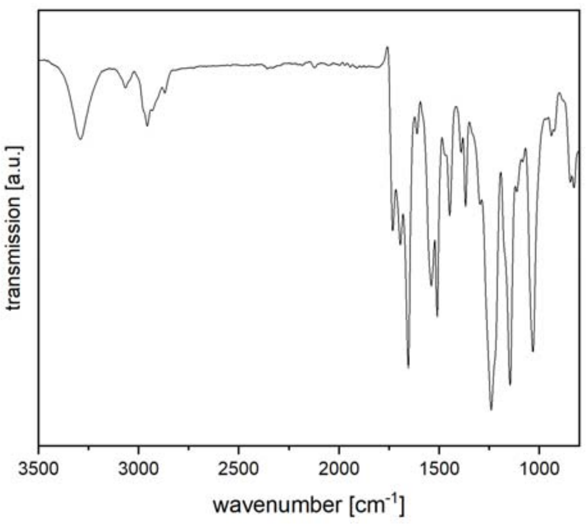

Figure S9: FT-IR (ATR) spectrum of dipeptide ≡—O-Tyr-Leu (**6**).

tert-Butyl-N^6-(((9*H*-fluoren-9-yl)methoxy)carbonyl)-N^2-((S)-2-((((9*H*-fluoren-9-yl)methoxy) carbonyl)amino)-3-(4-(prop-2-yn-1-yloxy)phenyl)propanoyl)-L-lysinate (**7**)

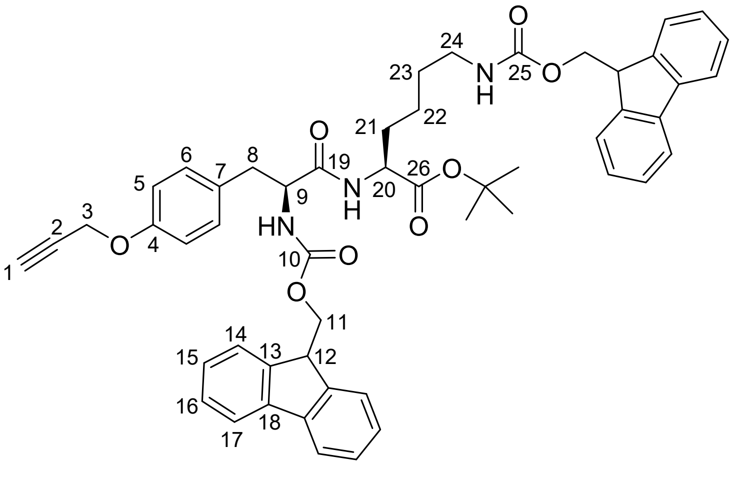

7

Dipeptide ≡—O-Tyr-Lys **7** was synthesized from 100 mg *N*-[(9*H*-fluoren-9-ylmethoxy)carbonyl]-*O*-2-propyn-1-yl-L-tyrosine **1** (0.23 mmol, 1.00 eq) and 104 mg *tert*-butyl-N^6-(((9*H*-fluoren-9-yl)methoxy)carbonyl)-L-lysinate hydrochloride **4** (0.23 mmol, 1.00 eq) according to the reported general procedure and obtained as a colorless solid.

Yield: 130 mg (0.15 mmol, 74 %). **Mp.** 144 – 147 °C. $\mathbf{R_f}$ 0.50 (cyclohexane/ethyl acetate, 1:1), **^{1}H-NMR** (400 MHz, $CDCl_3$): δ = 7.74 (d, $^3J_{17,16}$ = 7.64 Hz, 4H, H-17), 7.57 – 7.50 (m, 4H, H-14), 7.40 – 7.36 (m, 4H, H-16), 7.30 – 7.28 (m, 4H, H-15), 7.11 – 7.09 (m, 2H, H-5), 6.87 – 8.85 (m, 2H, H-6), 6.43 – 6.41 (m, 1H, amide-*H*), 5.39 – 5.37 (m, 1H, NH_{Tyr}), 4.96 – 4.93 (m, 1H, NH_{Lys}), 4.61 (d, $^4J_{3,1}$ = 2.36 Hz, 2H, H-3), 4.41 – 4.35 (m, 5H, H-9, H-20, H-11), 4.31 – 4.27

(m, 1H, H-11), 4.17 – 4.14 (m, 2H, H-12), 3.13 – 3.11 (m, 2H, H-24), 3.05 – 3.03 (m, 2H, H-8), 2.47 (t, $^{4}J_{1,3}$ = 2.36 Hz, 1H, H-1), 1.83 – 1.61 (m, 2H, H-21), 1.50 – 1.45 (m, 2H, H-23), 1.44 (s, 9H, H-26), 1.25 (m, 2H, H-22) ppm. **^{13}C-NMR** (100 MHz, $CDCl_3$): δ = 171.1 (C_q, C-28), 170.8 (C_q, C-19), 156.8 (C_q, C-10/25), 156.7 (C_q, C-4), 144.1 (C_q, C-13), 141.4 (C_q, C-18), 130.5 (C_t, C-5), 129.1 (C_q, C-7), 127.8 (C_t, C-16), 127.2 (C_t, C-15), 125.2 (C_t, C-14), 120.1 (C_t, C-17), 115.2 (C_t, C-6), 82.5 (C_q, C-27), 78.7 (C_t, C-2), 75.7 (C_t, C-1), 67.2 (C_s, C-11), 66.7 (C_s, C-11), 56.2 (C_t, C-20), 55.9 (C_s, C-3), 52.7 (C_t, C-9), 47.3 (C_t, C-12), 40.6 (C_s, C-24), 37.6 (C_s, C-8), 32.0 (C_s, C-21), 29.4 (C_s, C-23), 28.1 (C_p, C-26), 22.0 (C_s, C-22) ppm. **FT-IR (ATR)**: $\tilde{\nu}$ = 3309 (m, ν(C≡C-H)), 1720 (m, ν(C=O$_{ester}$)), 1695 (s, ν(C=O), amide I), 1657 (m, ν(C-N), amide II), 1537 (s, ν(C=C$_{arom}$)), 1450 (w), 1369 (w), 1292 (m, ν(C-O-C$_{ester}$)), 1257 (s, δ(C-O-C$_{ester}$)), 1173 (m), 1136 (m), 1030 (m, ν(C-O-C$_{ether}$)), 806 (m, δ(C-H$_{arom}$)) cm^{-1}. **HRMS** (ESI,+): found: 870.37432 for $C_{52}H_{53}N_3NaO_8$ [M+Na]$^+$; calc. 870.3730 for [M+Na]$^+$. **CHNS analysis**: found: C: 73.48 %, H: 6.23 %, N: 4.91 %; calc.: C: 73.65 %, H: 6.30 %, N: 4.96 %. **UV/Vis** (MeOH): λ_{max} = 207 nm.

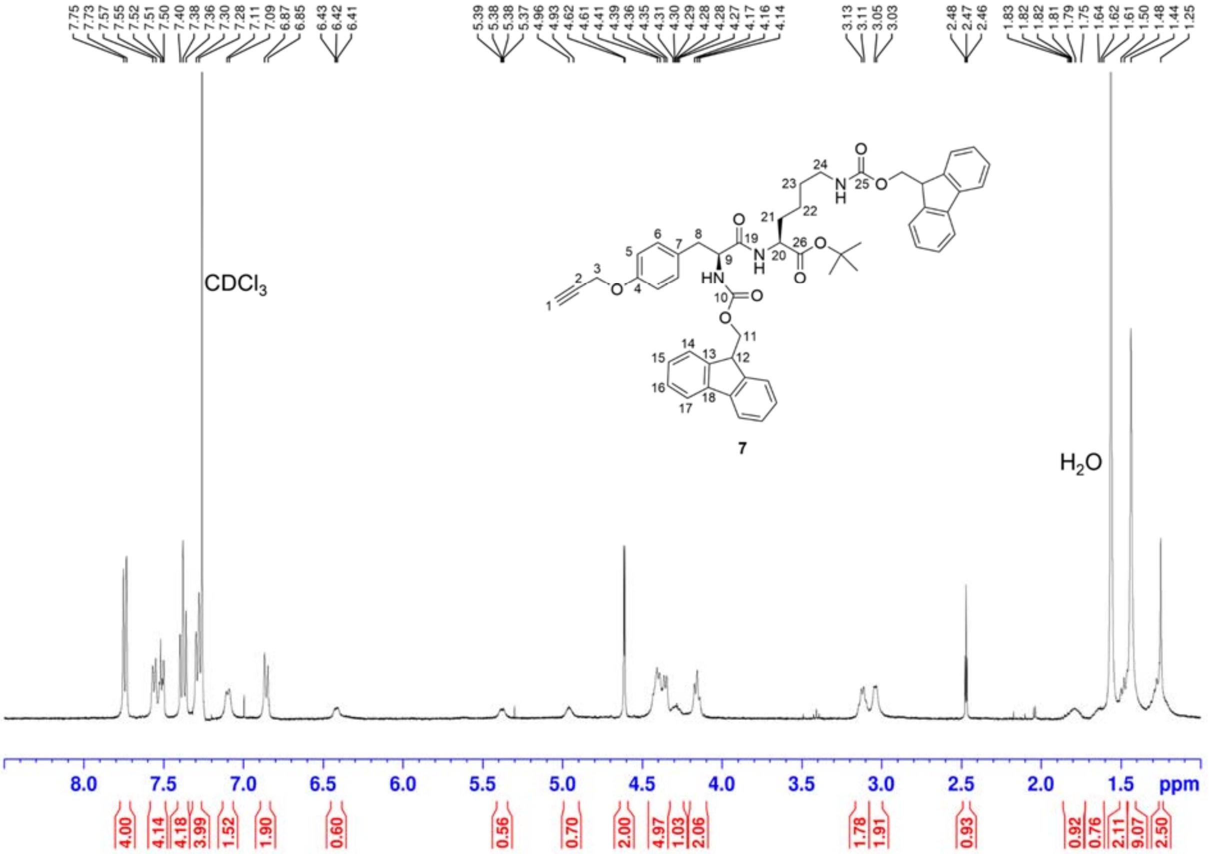

Figure S10: ^{1}H-NMR spectrum (400 MHz, $CDCl_3$) of dipeptide ≡─O-Tyr-Lys (**7**).

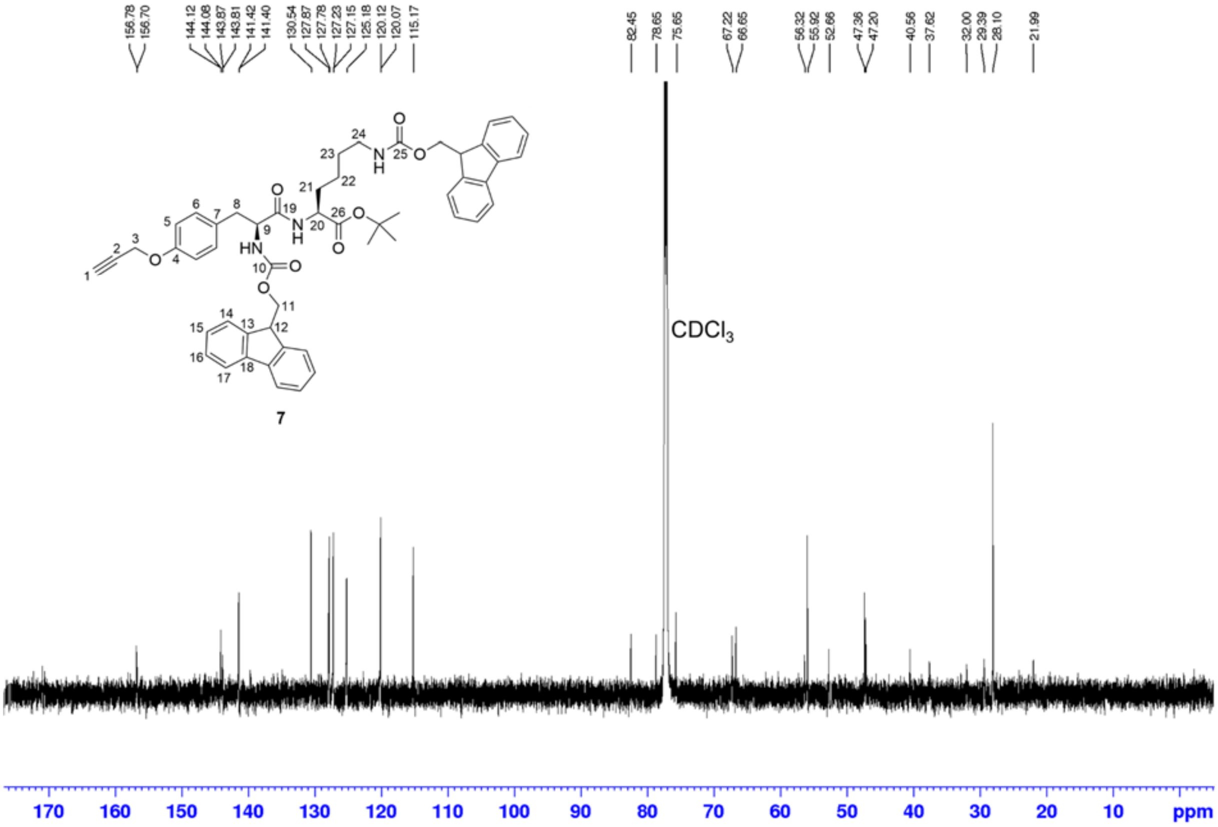

Figure S11: ^{13}C-NMR spectrum (101 MHz, $CDCl_3$) of dipeptide ≡─O-Tyr-Lys (**7**).

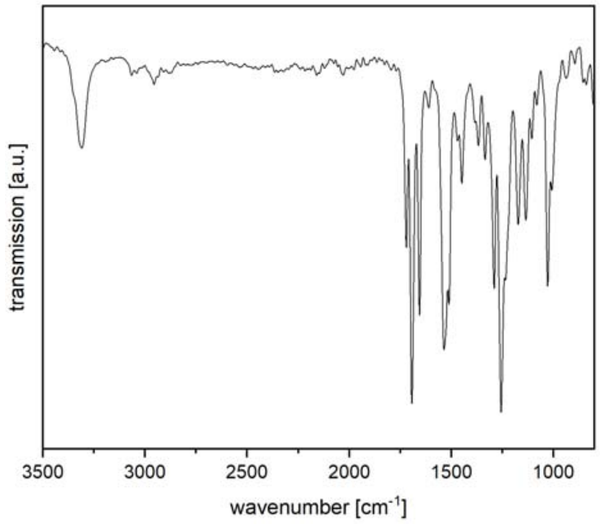

Figure S12: FT-IR (ATR) spectrum of dipeptide ≡─O-Tyr-Lys (**7**).

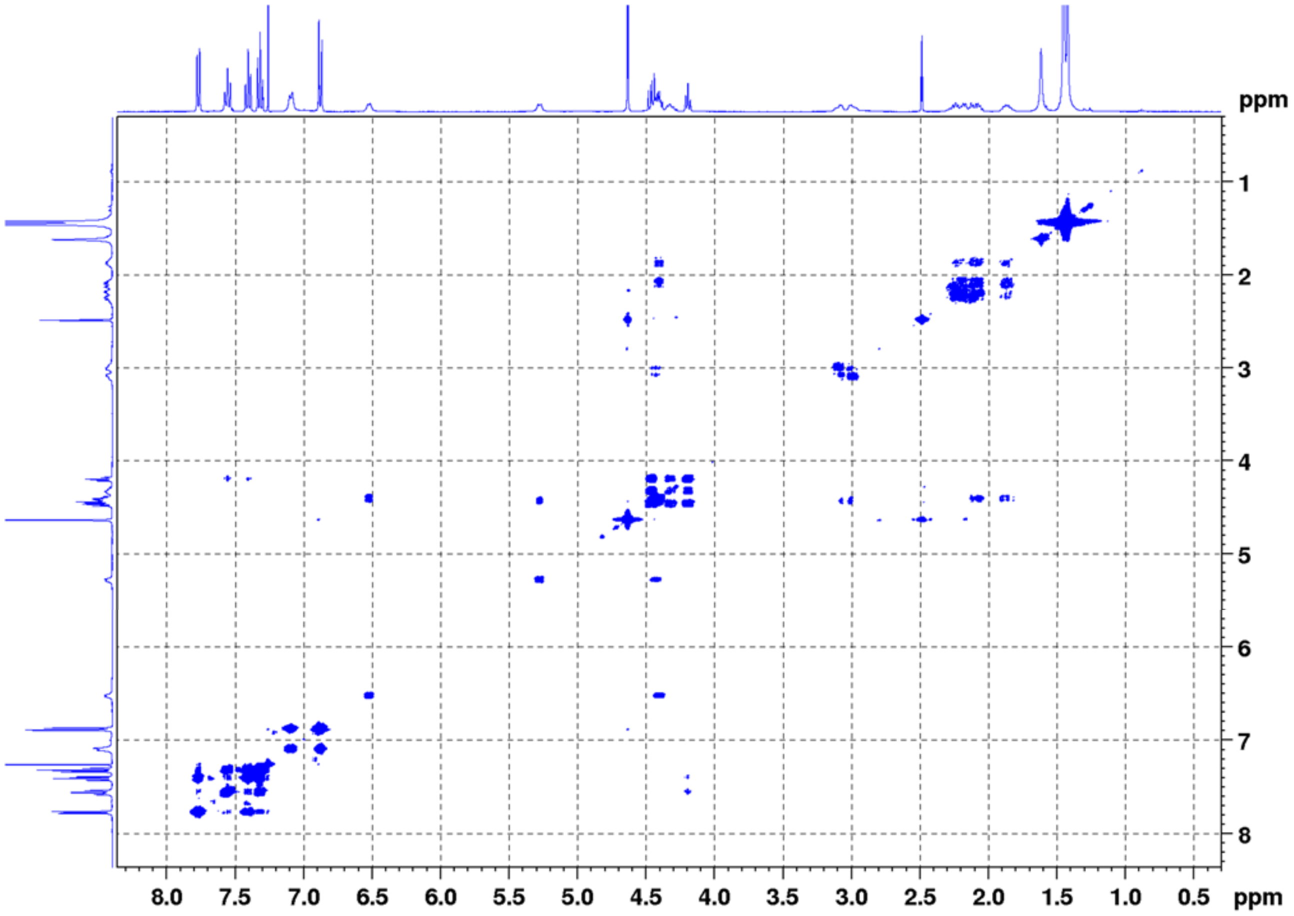

Figure S13: ^{1}H-^{1}H-COSY NMR spectrum (400 MHz, $CDCl_3$) of dipeptide ≡─O-Tyr-Glu (**5**).

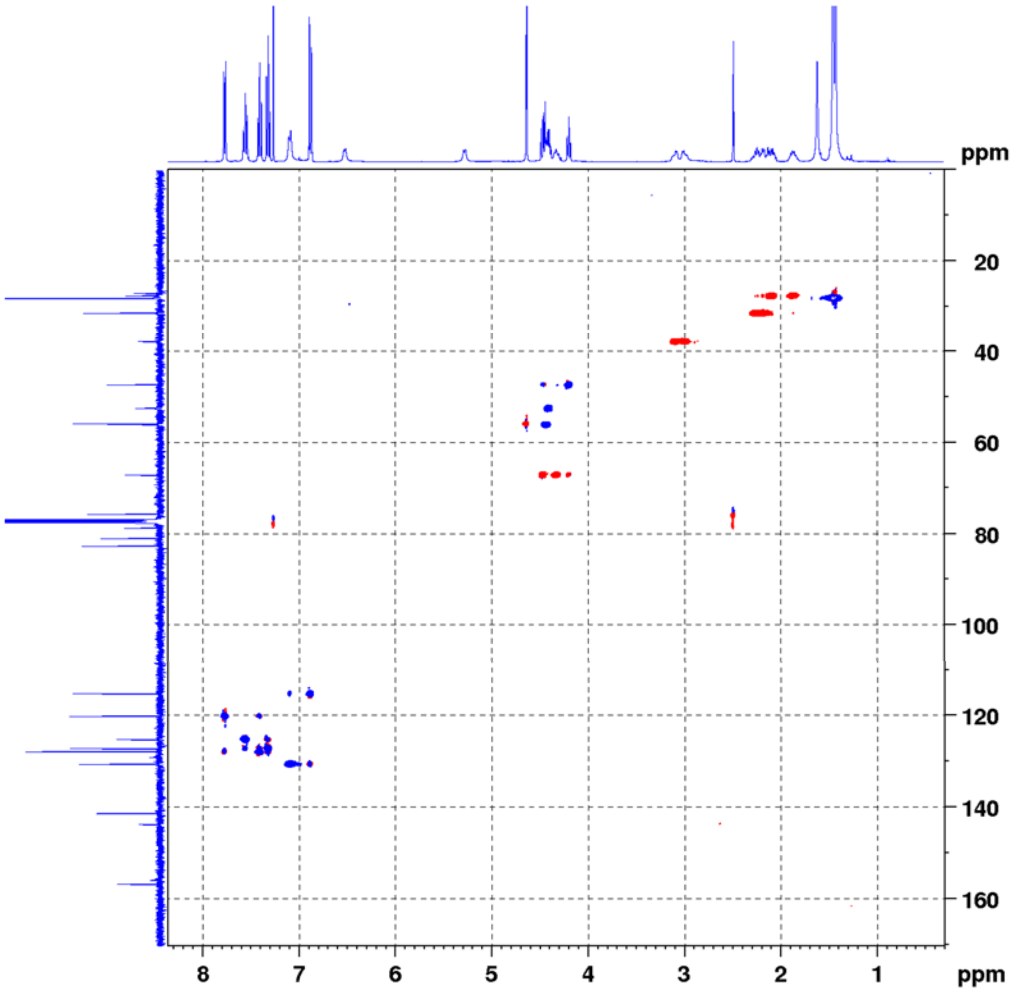

Figure S14: ^{1}H-^{13}C-HSQC NMR spectrum (400 MHz, $CDCl_3$) of dipeptide ≡─O-Tyr-Glu (**5**).

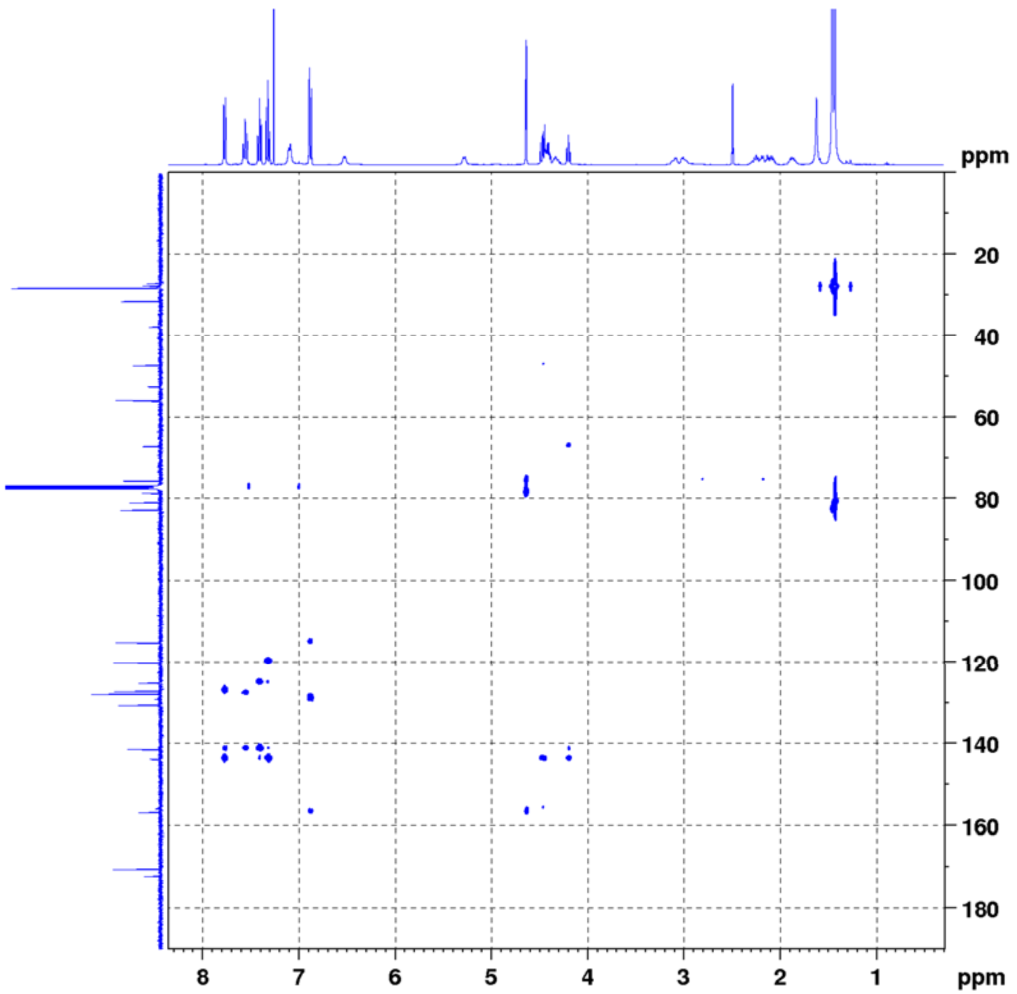

Figure S15: ^{1}H-^{13}C-HMBC NMR spectrum (400 MHz, $CDCl_3$) of dipeptide ≡─O-Tyr-Glu (**5**).

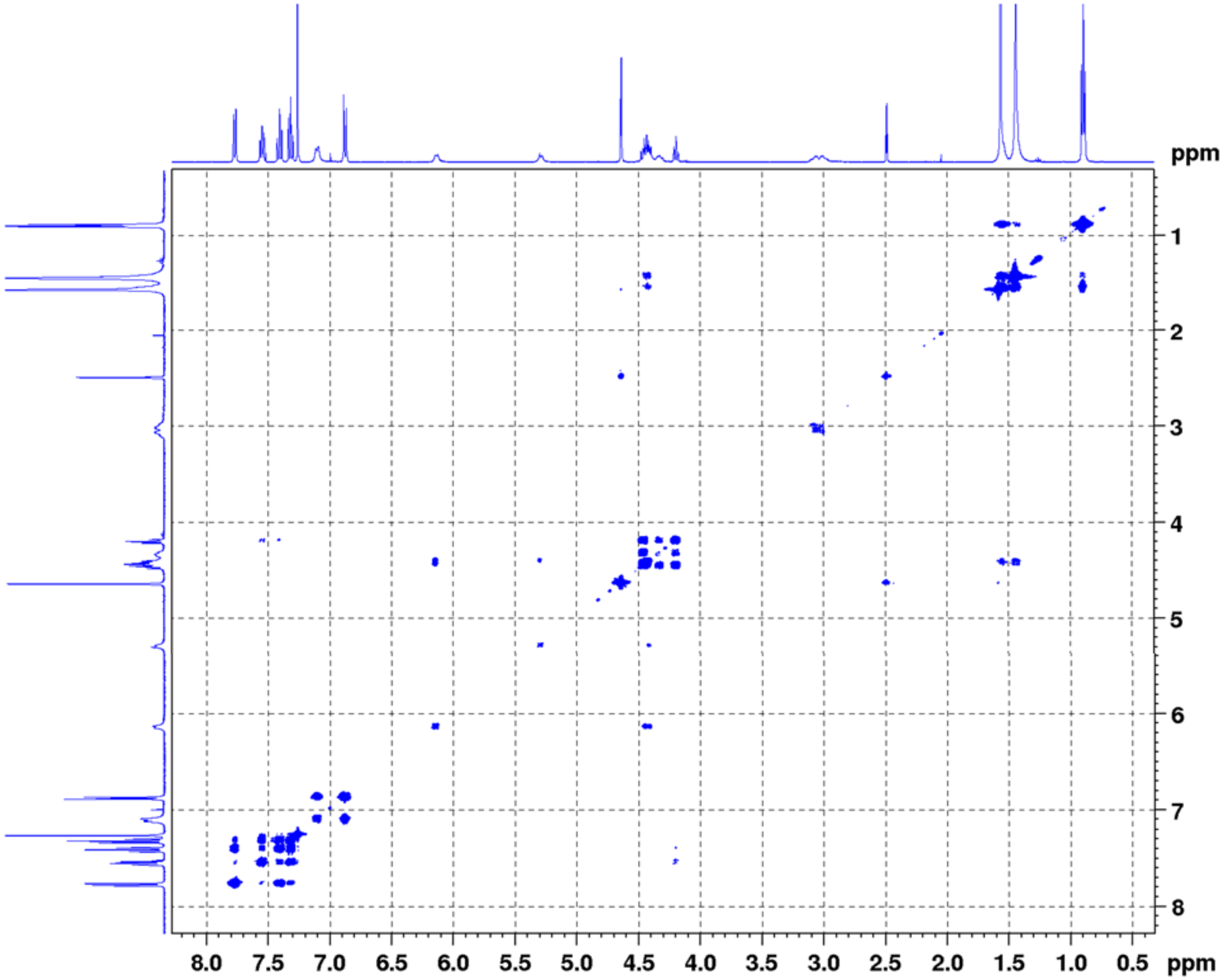

Figure S16: ^{1}H-^{1}H-COSY NMR spectrum (400 MHz, $CDCl_3$) of dipeptide ≡─O-Tyr-Leu (**6**).

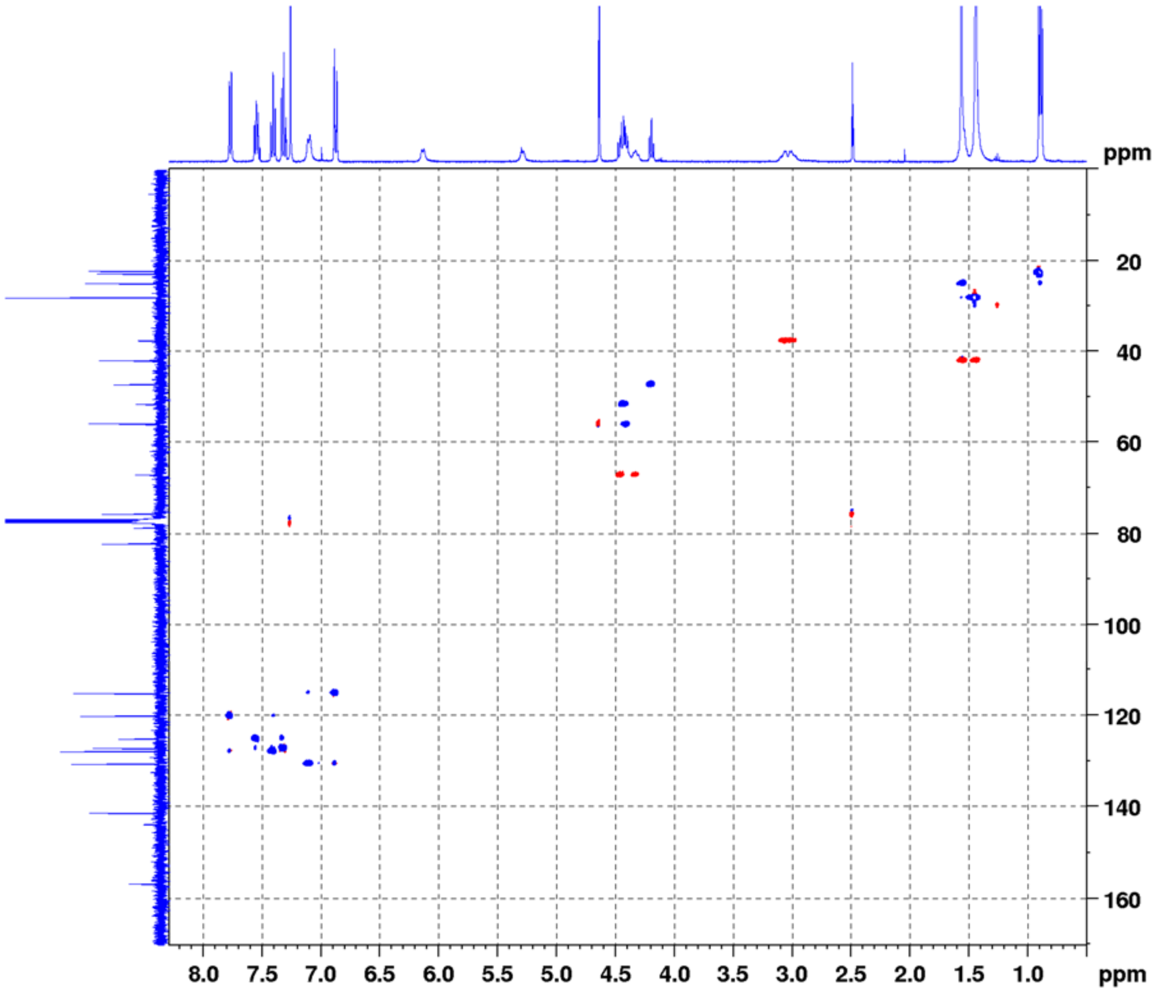

Figure S17: ^{1}H-^{13}C-HSQC NMR spectrum (400 MHz, $CDCl_3$) of dipeptide ≡─O-Tyr-Leu (**6**).

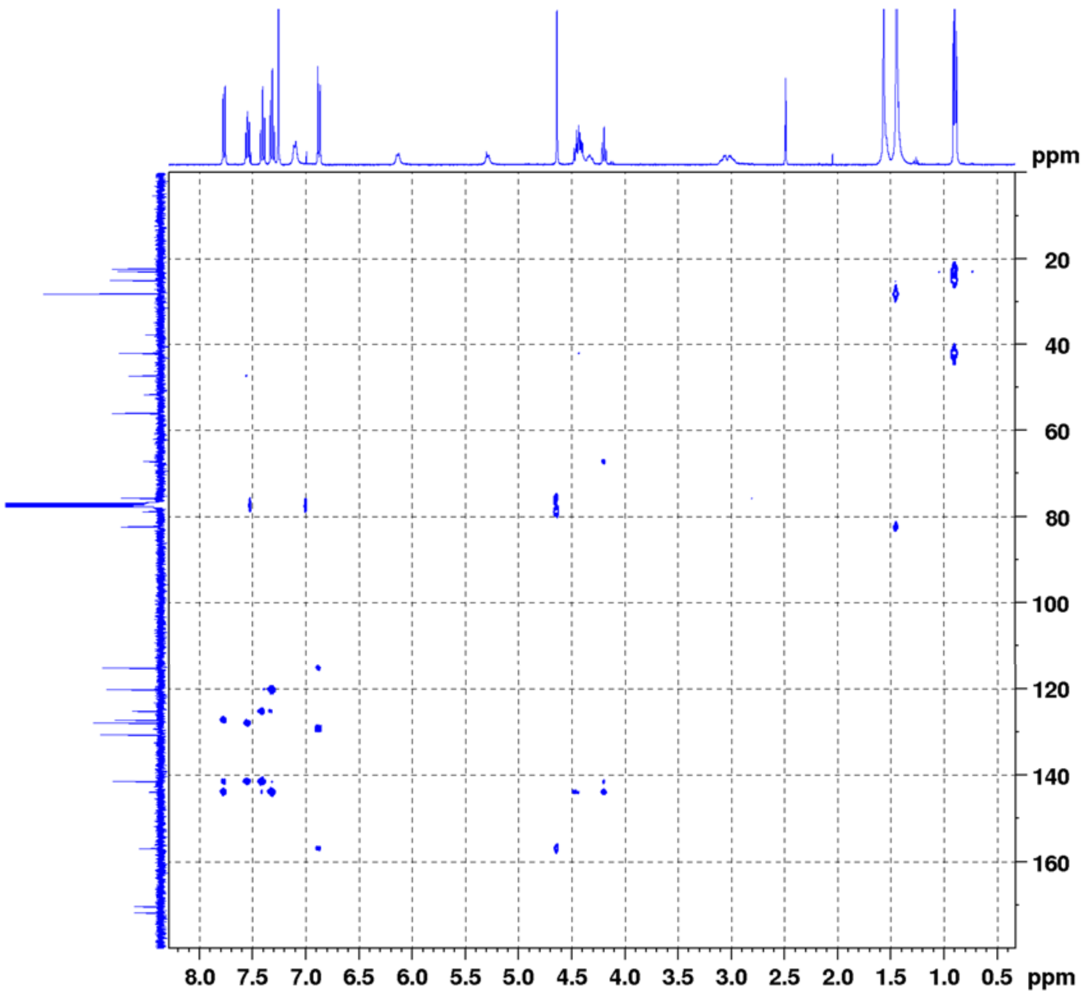

Figure S18: ^1H-^{13}C-HMBC NMR spectrum (400 MHz, $CDCl_3$) of dipeptide ≡–O-Tyr-Leu (**6**).

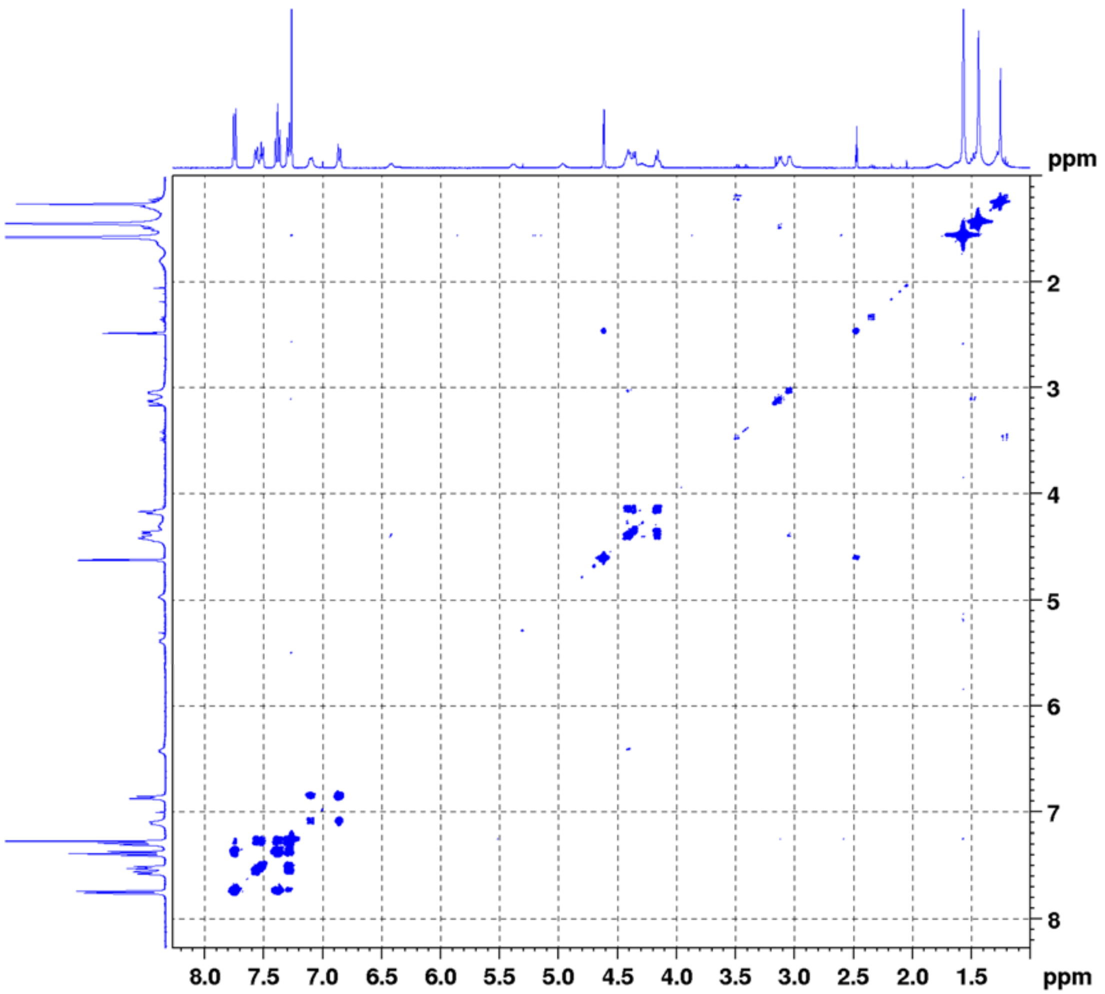

Figure S19: ^{1}H-^{1}H-COSY NMR spectrum (400 MHz, $CDCl_3$) of dipeptide ≡─O-Tyr-Lys (**7**).

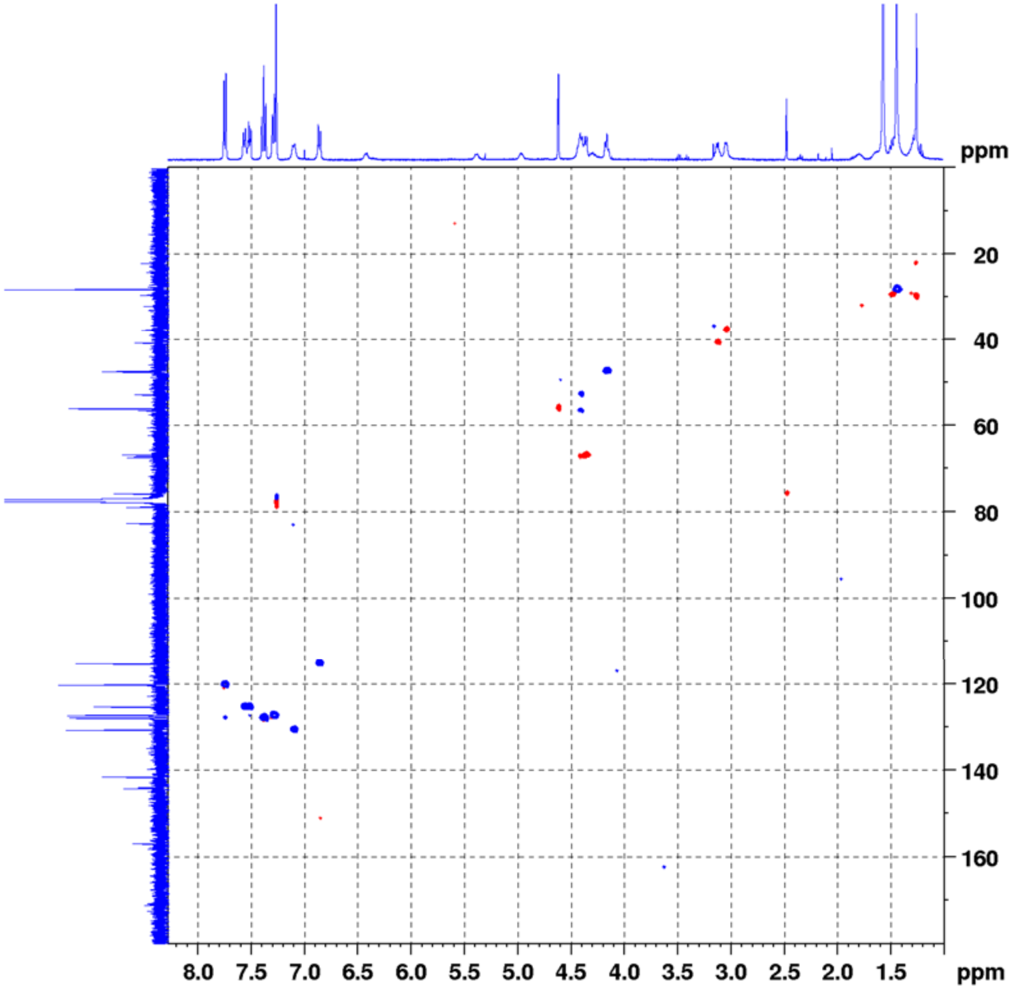

Figure S20: ^{1}H-^{13}C-HSQC NMR spectrum (400 MHz, $CDCl_3$) of dipeptide ≡─O-Tyr-Lys (**7**).

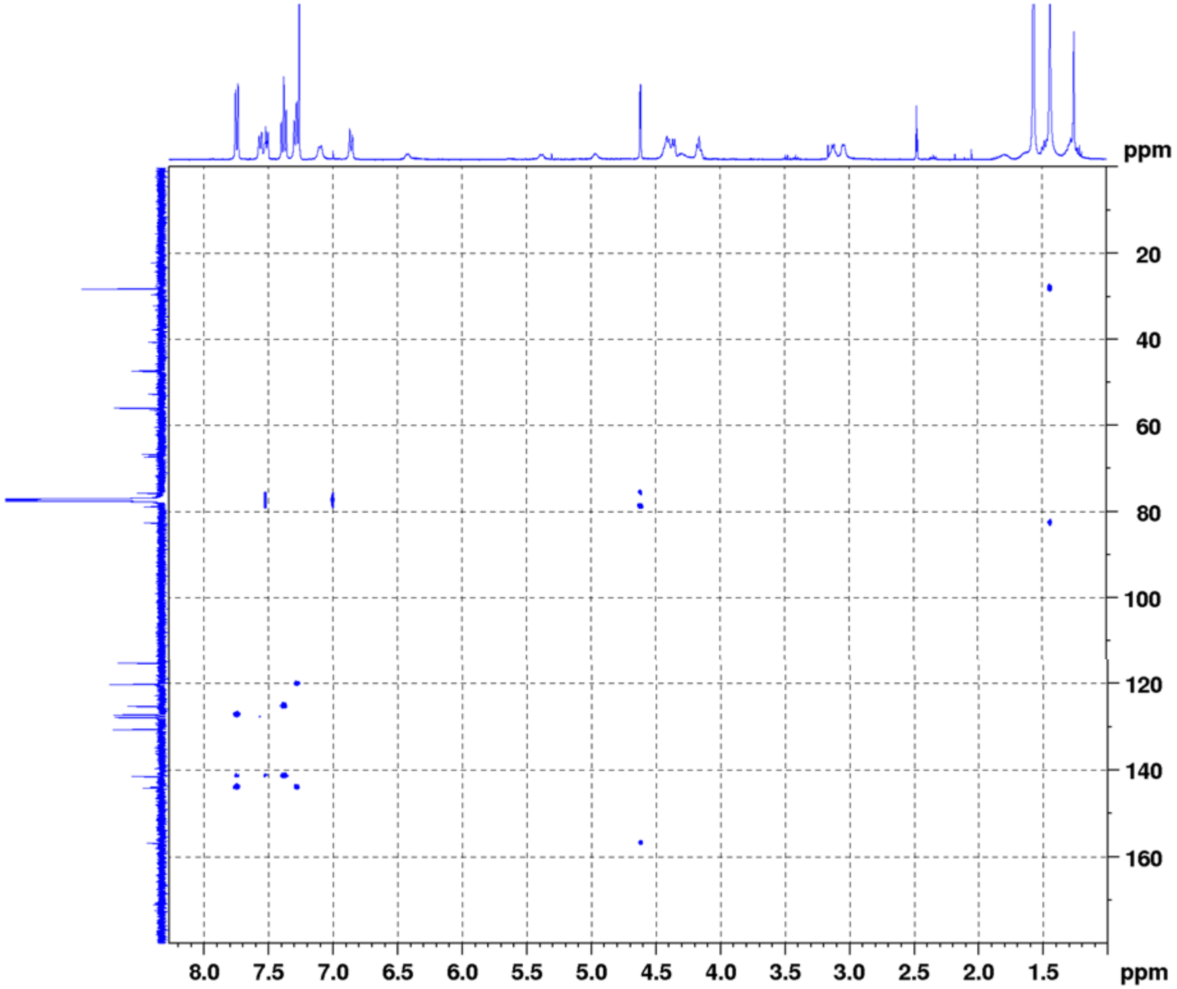

Figure S21: ^{1}H-^{13}C-HMBC NMR spectrum (400 MHz, $CDCl_3$) of dipeptide ≡–O-Tyr-Lys (**7**).

Electrospray ionization (ESI) mass spectrometry of synthesized dipeptides

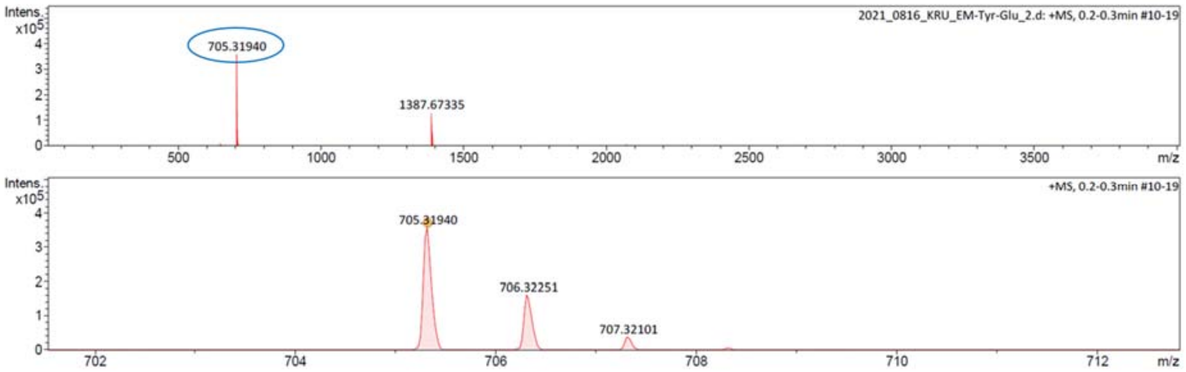

Figure S22: HRMS (ESI+): ≡—O-Tyr-Glu **5**, found: 705.31940 for $C_{40}H_{46}N_2NaO_8$ $[M+Na]^+$, calc. 705.3152.

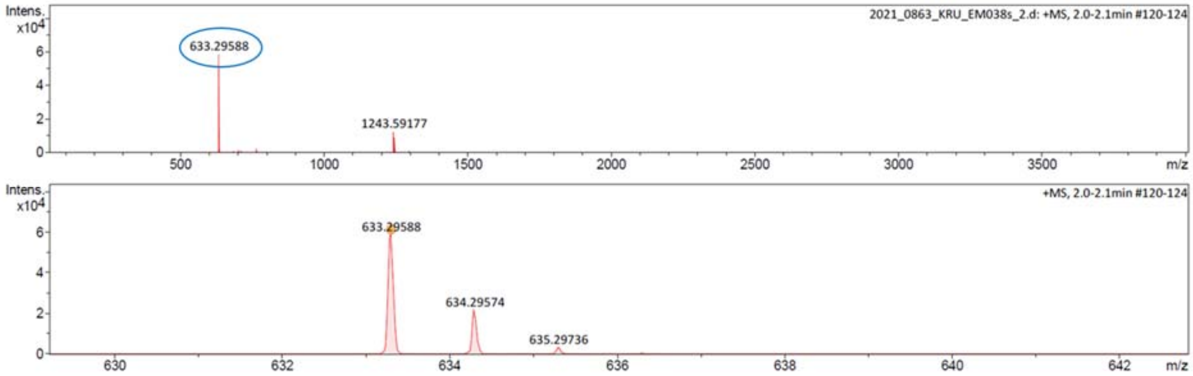

Figure S23: HRMS (ESI+): ≡—O-Tyr-Leu **6**, found: 633.29588 for $C_{37}H_{42}N_2NaO_6$ $[M+Na]^+$, calc. 633.2941.

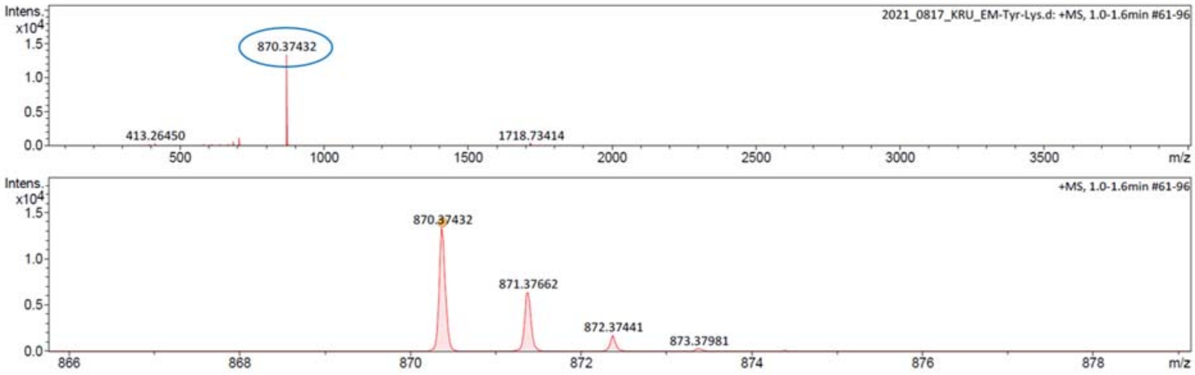

Figure S24: HRMS (ESI+): ≡—O-Tyr-Lys **7**, found: 870.37432 for $C_{52}H_{53}N_3NaO_8$ $[M+Na]^+$, calc. 870.3730.

Tandem mass spectrometry (MS/MS fragmentation) of synthesized dipeptides

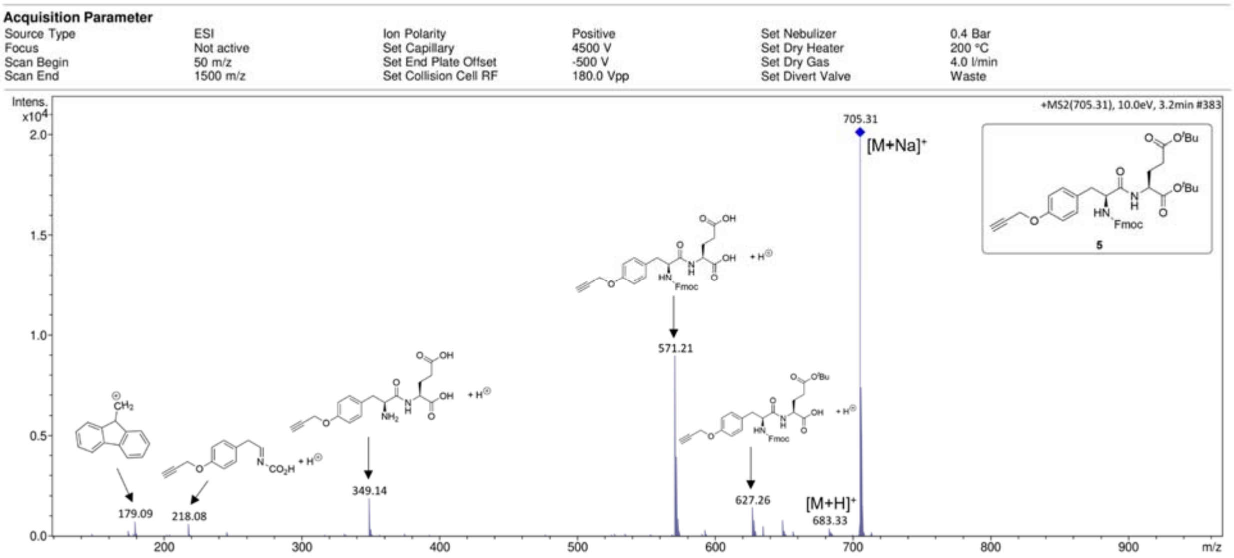

Figure S25: MS/MS (705.31, ESI+) fragmentation mass spectrum of dipeptide ≡—O-Tyr-Glu (**5**).

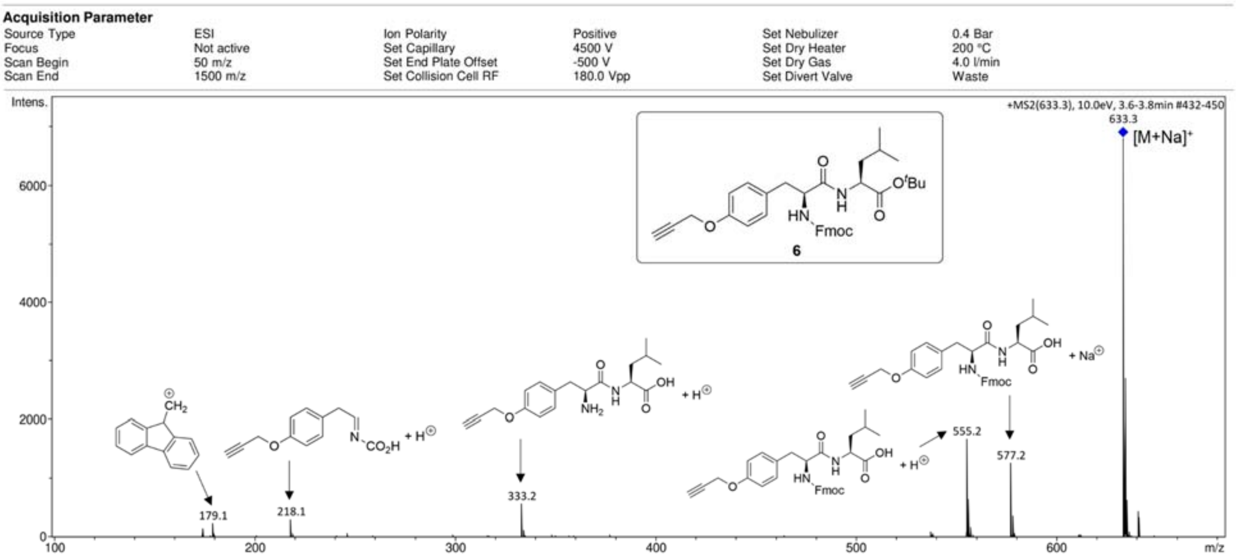

Figure S26: MS/MS (633.3, ESI+) fragmentation mass spectrum of dipeptide ≡—O-Tyr-Leu (**6**).

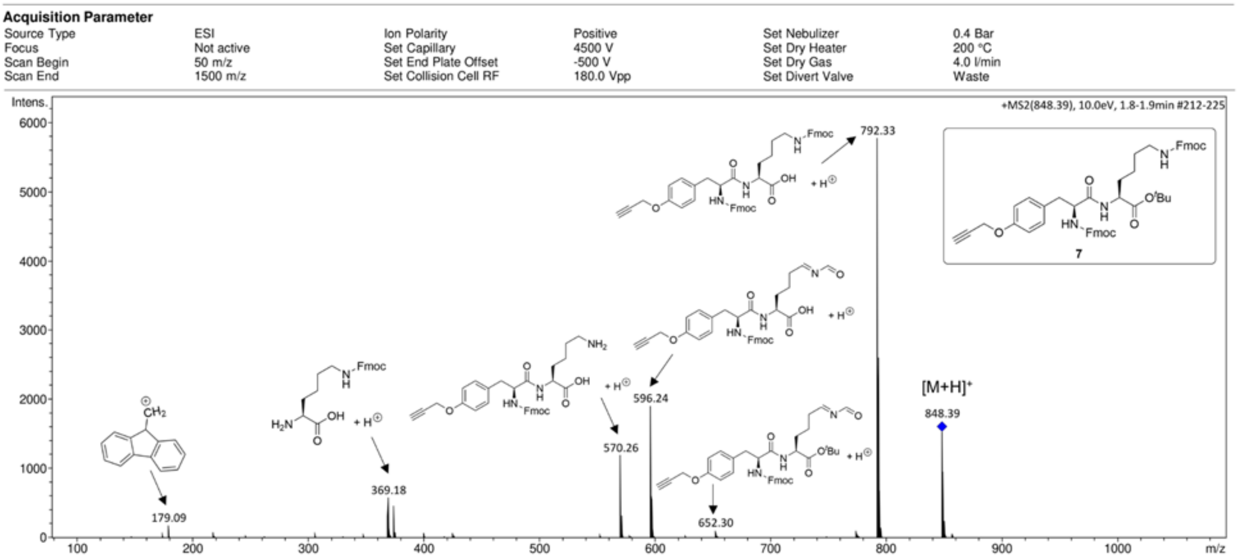

Figure S27: MS/MS (848.34, ESI+) fragmentation mass spectrum of dipeptide ≡─O-Tyr-Lys (**7**).

UV/Vis absorption spectra of synthesized dipeptides

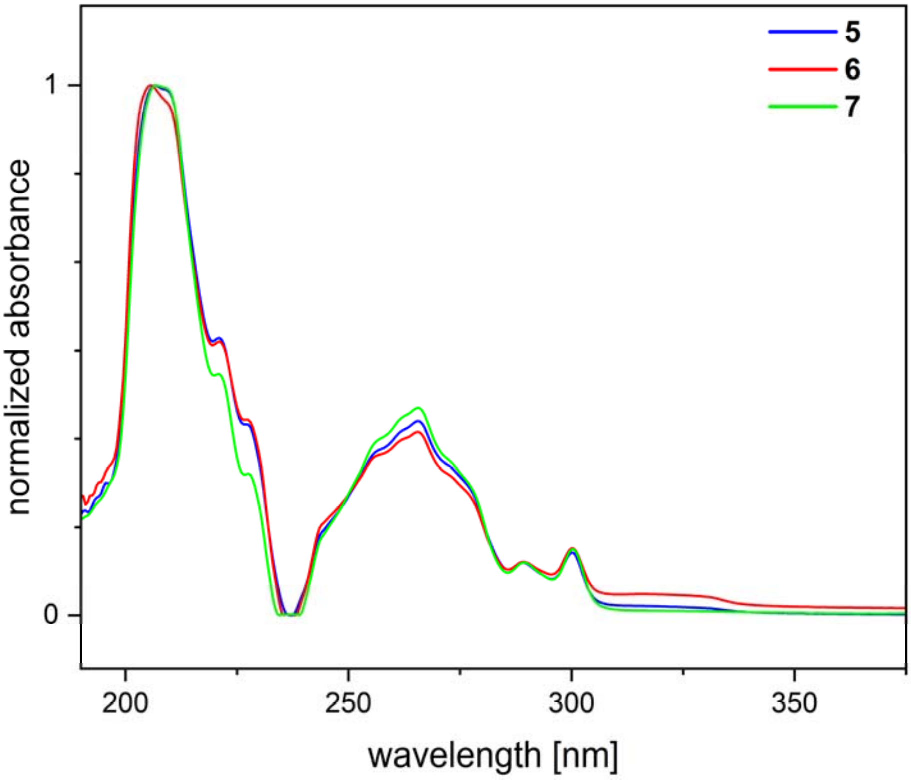

Figure S28: UV/Vis absorption spectra of dipeptides ≡─O-Tyr-Glu **5**, ≡─O-Tyr-Leu **6** and ≡─O-Tyr-Lys **7** (c(dipeptide) = $1\cdot10^{-5}$ mol/L) in methanol.

1.3 Materials and methods for ND characterization

FT-IR spectra were obtained with a *Jasco* FT-IR-410 spectrometer as KBr pellet dried *in vacuo* for ND samples. Diffuse reflectance infrared Fourier transform spectroscopy (DRIFTS) was performed for ND samples using a *Thermo Fischer* Nicolet iS5.

Surface loading of functionalized ND was determined by thermogravimetric analysis using a *Perkin-Elmer* TGA 4000 with a 20 $mL\cdot min^{-1}$ N_2 flowrate and a heating rate of 10 $K\cdot min^{-1}$ from 30 – 130 °C, a plateau time of 60 min at 130 °C and another heating phase from 130 – 900 °C at 5 $K\cdot min^{-1}$.

Particle size determination, zeta potential measurements and titration experiments were performed with a *Malvern* Zetasizer Nano ZS (dynamic light scattering, backscattering mode) equipped with autotitrator MPT-2. ND dispersions were measured in doubly distilled water (dd-H_2O, pH 6 – 7) at the intrinsic pH of the sample. Particle size distributions were obtained using the Marquardt method as volume distribution (Dv(10), Dv(50), Dv(90)) or as intensity distribution for the investigation of the colloidal stability in different media. For titration experiments, volumetric standard solutions of HCl (0.1 M and 0.01 M) and NaOH (0.1 M and 0.25 M) were used.

Atomic force microscopy (AFM) images were recorded with a *Bruker* Dimension Icon instrument operating in tapping mode in air. Dipeptide functionalized ND particles were deposited on a silicon wafer by dropcasting the colloidal dispersions followed by blow-drying with nitrogen. The images were processed and analysed using Gwyddion software.

Acid-purified detonation nanodiamond (diamond content > 95 %) was purchased from *Gansu Lingyun Corp.* (China) and milled according to a previously reported procedure.[2] Briefly, 9.00 g of pristine detonation ND powder was dispersed in 400 mL dd-H_2O using an ultrasonic bath and milled in an attrition mill with 0.05 mm-sized zirconia beads at 4200 rpm. After 2 h of milling, the particle size was small enough and the stable, black dispersion was collected (350 mL) with a final concentration of 32 $mg\cdot mL^{-1}$. For further functionalizations, ND was washed and redispersed in dd-H_2O.

A colorimetric assay (Kaiser test) to proof the existence of primary amino groups after deprotection of the attached dipeptides was performed according to literature.[3] In brief, 1 mg of nanodiamond sample was dispersed in 1 mL of doubly distilled water and 1 mL of acetate buffer solution for 15 min. Subsequently, 1 mL of KCN and 1 mL of pyridine solution were added to the mixture, which was then heated at 120 °C for 10 min followed by addition of 1 mL of ninhydrin solution. The mixture was stirred for another 10 min and then cooled to room temperature over the course of 30 min. After that, 5 mL of ethanol solution was added. In the presence of primary amino groups, the colour of the reaction mixture changes from yellow to

purple. The ND particles were separated by centrifugation and the supernatant was investigated by UV/Vis spectroscopy. A detailed instruction for preparing the above mentioned solutions can be found in literature.[3]

1.4 Particle syntheses and characterization

Milled detonation nanodiamond (**mND**)[1]

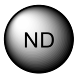

mND

Milling of detonation ND was achieved according to earlier described procedure (see part materials and methods for ND characterization).
FT-IR (KBr): $\tilde{\nu}$ =3388 (br), 2923 (w, ν(C-H)), 1626 (s), 1376 (w), 1325 (w), 1266 (w), 1102 (m), 1039 (w), 884 (w), 833 (w), 691 (w), 653 (w) cm^{-1}. **Zeta potential**: +45.7 mV (dd-H_2O, intrinsic pH = 4.7). **Particle size (DLS)**: 10 % ≤ 4.49 nm, 50 % ≤ 6.67 nm, 90 % ≤ 12.8 nm (dd-H_2O). **Surface loading (TGA)**: no significant surface loading.

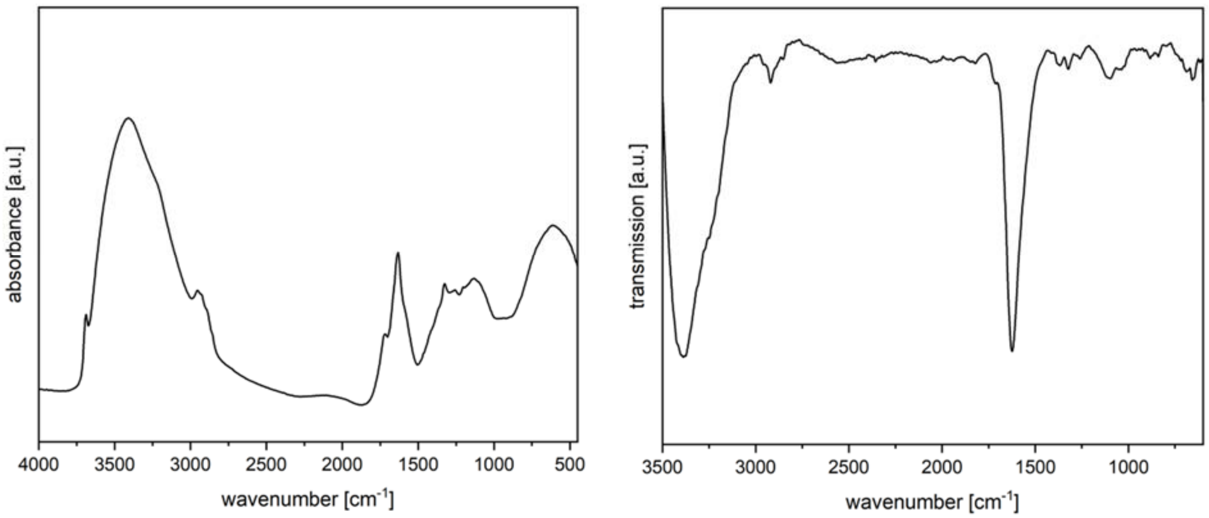

Figure S29: DRIFT spectrum (left) and FT-IR (KBr pellet) spectrum (right) of milled detonation nanodiamond **mND**.

Azide functionalized ND (**ND-N_3**)[1]

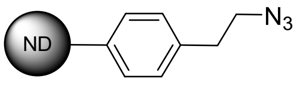

ND-N_3

3 mL of **mND** dispersion (32.0 mg·mL^{-1}, 96 mg nanodiamond) were dispersed in 30 mL 1 N HCl in an ultrasonic bath for 30 min. Subsequently, the dispersion was heated up to 80 °C and 100 mg of 4-(2-Azidoethyl)benzenamine (**8**, 0.62 mmol) and 0.10 mL of amyl nitrite (90.0 mg, 0.77 mmol) were added. After stirring the dispersion overnight at 80 °C, it was cooled to room temperature. The functionalized ND particles were isolated via centrifugation (15 k rpm) and washed repeatedly with acetone (3x), DMF (3x), acetone (3x), DMF (3x) and finally dd-H_2O (3x). For further characterization, a small amount of dispersion was dried at 80 °C.

Yield: 93.5 mg. **FT-IR (KBr)**: $\tilde{\nu}$ = 3466 (br), 2956 (w, ν(C-H)), 2139 (w, $\nu(N_3)$), 1664 (m), 1418 (w), 911 (w) cm^{-1}. **Zeta potential**: +38.2 mV (dd-H_2O, intrinsic pH = 7.1). **Particle size (DLS)**: 10 % ≤ 39.7 nm, 50 % ≤ 58.2 nm, 90 % ≤ 99.6 nm (dd-H_2O). **Surface loading (TGA)**: 0.22 mmol·g^{-1} = Δm (145 – 460 °C) = −3.3 %.

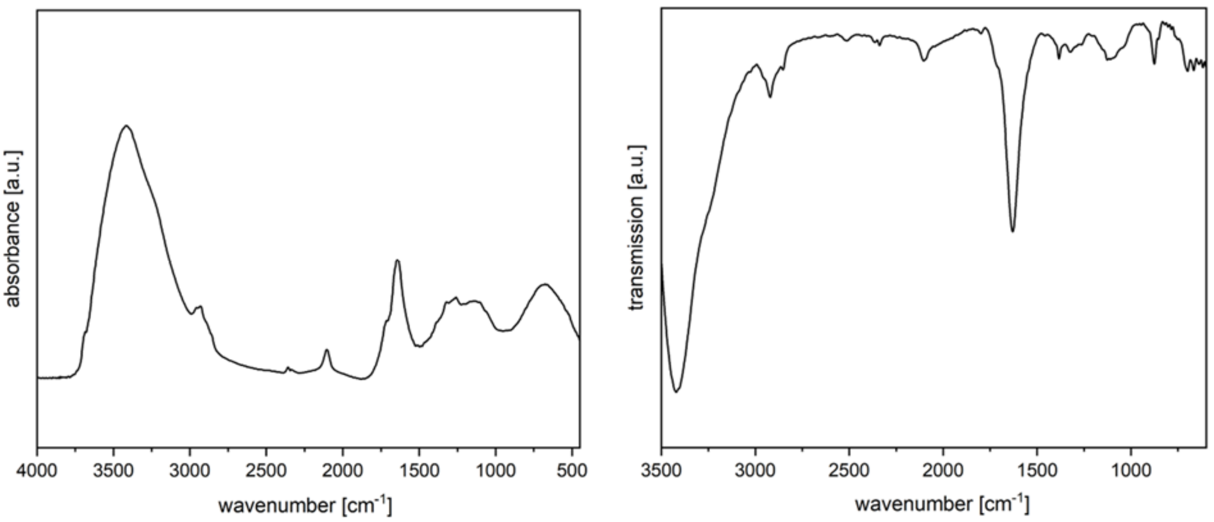

Figure S30: DRIFT spectrum (left) and FT-IR (KBr pellet) spectrum (right) of azide functionalized nanodiamond **ND-N_3**.

General procedure for functionalization of ND with dipeptides by click chemistry

ND-N_3 (40 mg) was washed with DMF and redispersed in 6 mL degassed DMF. Under N_2 atmosphere, the ND dispersion was degassed through N_2 bubbling and simultaneous sonication for 15 min followed by addition of 13.3 mg cupper(II) sulfate (0.08 mmol, 1.00 eq)

and sonication for another 15 min. After 33.3 mg sodium ascorbate (0.16 mmol, 2.00 eq) was added, the reaction mixture was stirred for 30 min before 20 mg of the respective dipeptide was added. The resulting reaction mixture was stirred under N_2 atmosphere at room temperature for 5 d. Completion of the reaction was monitored by DRIFTS (disappearance of the azide band at 2100 cm^{-1}). The particles were isolated by centrifugation (15 k rpm, 15 min) and washed repeatedly with dd-H_2O (3x), DMF (3x), 0.1 M ethylenediaminetetraacidic acid (EDTA) solution and again with dd-H_2O (3x).

For subsequent cleavage of the dipeptide protecting groups, the respective ND conjugate (**ND-Tyr-Glu^{x}**, **ND-Tyr-Leu^{x}** or **ND-Tyr-Lys^{x}**, x in superscript reffers to dipeptide functionalized NDs <u>before</u> deprotection) (40 mg) was washed twice with DMF and added to a solution (20 mL) of 20 % piperidine in DMF. The reaction mixture was sonicated for 30 min. Afterwards, the fmoc deprotected particles were isolated by centrifugation (15 k rpm, 15 min) and washed with dd-H_2O (4x). To remove *tert*-butyl protecting groups in the next step, fmoc deprotected ND was washed with dichloromethane and transferred into a solution of trifluoroacetic acid/dichloromethane (1:1, v/v). The reaction mixture was sonicated for 30 min followed by isolation of the particles by centrifugation (10 k rpm, 10 min). Finally, the resulting ND particles were washed with dd-H_2O (4x) giving zwitterionic dipeptide functionalized ND conjugates (**Tyr-Glu**, **ND-Tyr-Leu** or **ND-Tyr-Lys**). For further characterization, a small amount of ND dispersion was lyophilized.

Tyrosylglutamic acid functionalized ND (**ND-Tyr-Glu**)

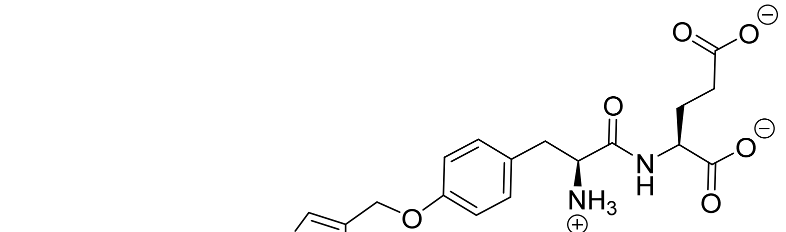

ND-Tyr-Glu

Synthesis of **ND-Tyr-Glu** was conducted following the above-mentioned general procedure for click reaction of azide functionalized **ND-N_3** (40 mg) with 20 mg of dipeptide ≡–O-Tyr-Glu **5** (0.03 mmol).

Yield: 38.2 mg. **FT-IR (KBr)**: $\tilde{\nu}$ = 3400 (br, ν(O-H)), 2920 (w, ν(C-H)), 2850 (w, ν(C-H)), 1676 (s, ν(C=O), amide I), 1633 (s, ν(CO_2^-), 1460 (w), 1431 (w), 1383 (w, δ(C-H)), 1323 (w, ν(CO_2^-)),

1259 (w), 1203 (m, ν(C-O_{ether})), 1138 (m, ν(C-O_{ether})), 879 (w), 839 (w), 804 (w, δ(C-$H_{1,4\text{-}arom}$), 723 (w) cm^{-1}. **Zeta potential**: +39.7 mV (dd-H_2O, intrinsic pH = 4.4). **Particle size (DLS)**: 10 % ≤ 29.6 nm, 50 % ≤ 43.9 nm, 90 % ≤ 97.0 nm (dd-H_2O). **Surface loading (TGA)**: 0.08 mmol·g^{-1} = Δm (145 – 430 °C) = −3.8 %.

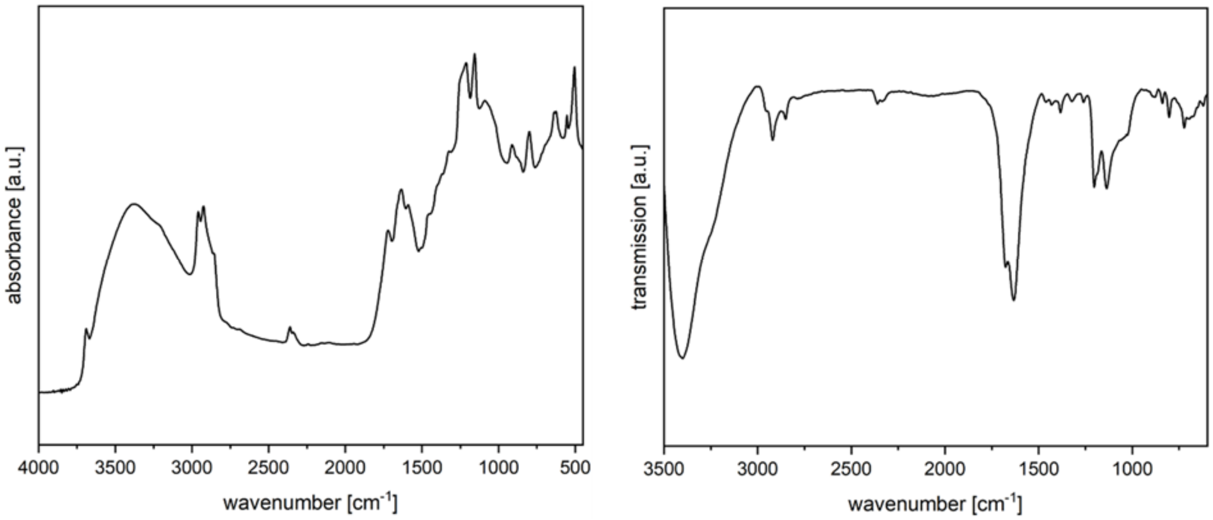

Figure S31: DRIFT spectrum (left) and FT-IR (KBr pellet) spectrum (right) of tyrosylglutamic acid functionalized nanodiamond **ND-Tyr-Glu**.

Tyrosylleucine functionalized ND (**ND-Tyr-Leu**)

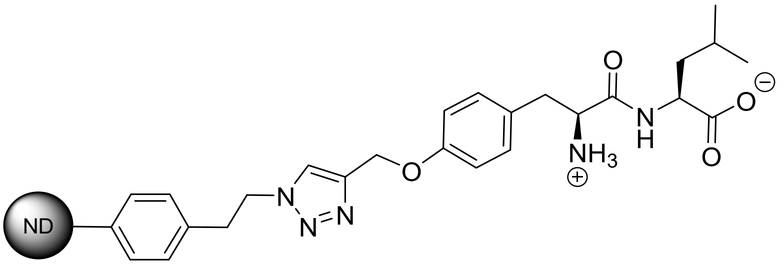

ND-Tyr-Leu

Synthesis of **ND-Tyr-Leu** was conducted following the above-mentioned general procedure for click reaction of azide functionalized **ND-N$_3$** (40 mg) with 20 mg of dipeptide ≡—O-Tyr-Leu **6** (0.03 mmol).

Yield: 36.9 mg. **FT-IR (KBr)**: $\tilde{\nu}$ = 3396 (br, ν(O-H)), 2918 (w, ν(C-H)), 2852 (w, ν(C-H)), 1678 (s, ν(C=O), amide I), 1633 (s, ν(CO_2^-), 1431 (w), 1383 (w, δ(C-H)), 1323 (w, ν(CO_2^-)), 1203 (m, ν(C-O_{ether})), 1138 (m, ν(C-O_{ether})), 874 (w), 839 (w), 804 (w, δ(C-$H_{1,4\text{-}arom}$), 723 (w) cm^{-1}. **Zeta potential**: +40.7 mV (dd-H_2O, intrinsic pH = 4.8). **Particle size (DLS)**: 10 % ≤ 45.5 nm, 50 %

≤ 67.8 nm, 90 % ≤ 122 nm (dd-H_2O). **Surface loading (TGA)**: 0.07 mmol·g^{-1} = Δm (140 – 470 °C) = −3.5 %.

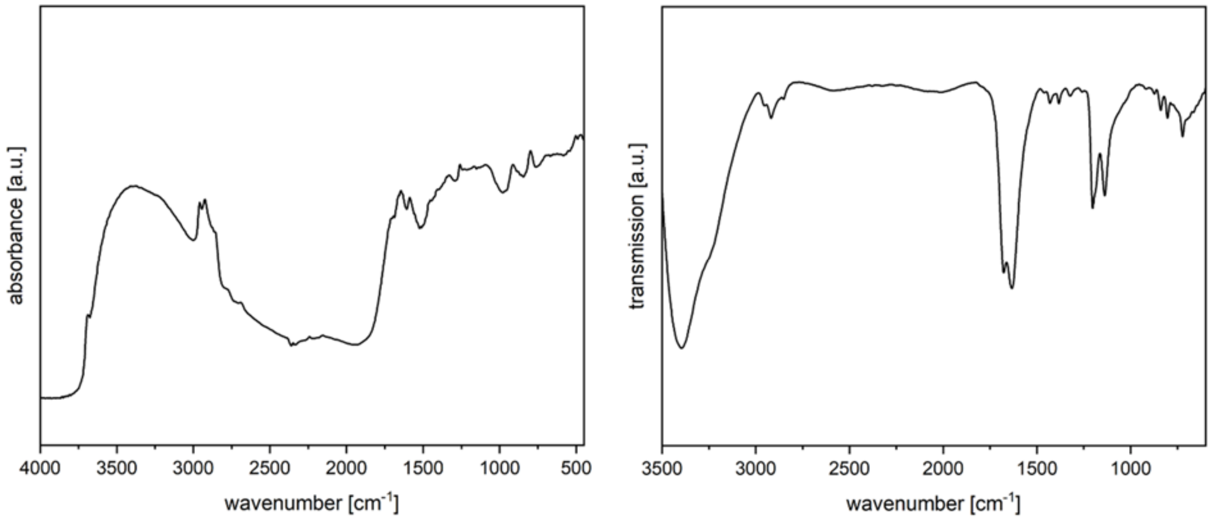

Figure S32: DRIFT spectrum (left) and FT-IR (KBr pellet) spectrum (right) of tyrosylleusine functionalized nanodiamond **ND-Tyr-Leu**.

Tyrosyllysine functionalized ND (**ND-Tyr-Lys**)

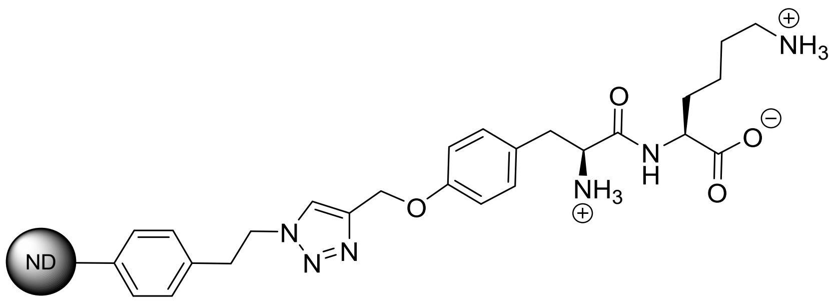

ND-Tyr-Lys

Synthesis of **ND-Tyr-Lys** was conducted following the above-mentioned general procedure for click reaction of azide functionalized **ND-N_3** (40 mg) with 20 mg of dipeptide ≡—O-Tyr-Lys **7** (0.03 mmol).

Yield: 38.7 mg. **FT-IR (KBr)**: $\tilde{\nu}$ = 3400 (br, ν(O-H)), 2922 (w, ν(C-H)), 2852 (w, ν(C-H)), 1680 (s, ν(C=O), amide I), 1635 (s, ν(CO_2^-), 1456 (w), 1431 (w), 1383 (w, δ(C-H)), 1321 (w, ν(CO_2^-)), 1203 (m, ν(C-O_{ether})), 1138 (m, ν(C-O_{ether})), 939 (w), 879 (w), 839 (w), 804 (w, δ(C-$H_{1,4\text{-}arom}$), 723 (w) cm^{-1}. **Zeta potential**: +40.8 mV (dd-H_2O, intrinsic pH = 5.1). **Particle size (DLS)**: 10 % ≤ 52.3 nm, 50 % ≤ 76.4 nm, 90 % ≤ 128 nm (dd-H_2O). **Surface loading (TGA)**: 0.07 mmol·g^{-1} = Δm (130 – 470 °C) = −3.4 %.

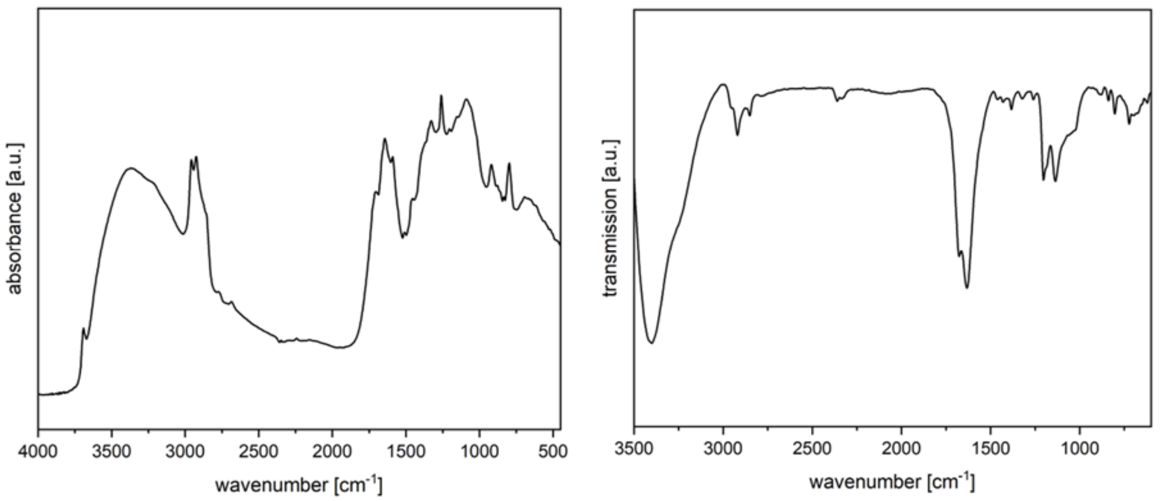

Figure S33: DRIFT spectrum (left) and FT-IR (KBr pellet) spectrum (right) of tyrosyllysine functionalized nanodiamond **ND-Tyr-Lys**.

1.5 Methods for *in vitro* experiments

The human lung cancer epithelial cell line A549 (ATCC-CCL-185) was obtained from the American Type Culture Collection (*ATCC*, Manassas, VA, USA) and grown in Dulbecco's Modified Eagle Medium (DMEM) supplemented with 10 % FBS (product no. 26140079, *Thermo Fisher Scientific*) and 1 % penicillin/streptomycin (Pen-Strep, product no. 15070063, *Thermo Fisher Scientific*), in an incubator with humidified atmosphere at 37 °C, 5 % CO_2. The cells were cultured at a density of approximately 1.3 x 10^4 cells/cm^2 (passage number below 15) in vented cell culture flasks and sub-cultured twice per week by trypsinization (0.25 % trypsin for 2 – 4 min at 37 °C). The A549 cells were used in the impedance-based assay.

Previously isolated CaLH3 and NOF from human oral tissues were cultured as described elsewhere.[4] Cells were cultured at 37 °C in a 5 % CO_2 humidified environment. For cellular toxicity assay, cells were cultured at a density of 1x10^5 cells/well in 12-well plates (*Sarstedt*, DE) in their respective medium and allowed to attach for 24 h. After 24 h, the cells were washed with serum-free medium and treated with different concentrations of the NDs in serum-free medium. After 48 h of treatment, the cells were washed with PBS, trypsinized and live cells were manually counted based on the trypan blue exclusion principle.[5]

The use of the primary cells has been approved by the Regional Ethical Committee for Western Norway under project number REKVest 2013/1492.

Impedance-based cell viability assay xCELLigence®

To validate the putative cytotoxic effects of different NDs, cell viability was also assessed using a label-free real-time impedance-based method using the xCELLigence® system

(xCELLigence RTCA, *Agilent*).[6-7] Assays based on electrical impedance have the advantage of being less prone to possible ND-induced interferences with assay readings and reagents. They also provide an evaluation of dynamic cell responses, such as cell viability, cell number, cell-substrate and cell-cell contact in real-time. The change in measured electrical impedance is delivered by the instrument as a dimensionless cell index (CI) value, which reflects cells' attachment and coverage of the electrodes onto which cells are seeded and the integrity of their membranes. The optimal seeding density of A549 cells for the chosen exposure period to NDs was determined based on an initial impedance-based screening of the cells at different densities. Following this, the A549 cells/well were seeded at a density of 2.000 cells/cm^2 in 16-well E-plates (*Agilent*) containing gold-plated electrodes at the bottom of the wells. Then, E-plates were inserted in the xCELLigence real-time impedance analyser and incubated at 37 °C and 5 % CO_2. After 24 h, the cells were treated for 48 h with concentrations of NDs corresponding to those used in the other cytotoxicity assays in this study. The treated cells were monitored for 5 days, and untreated cells served as controls. Electrical impedance was monitored in real-time every 15 min at a 10 kHz AC frequency. Detailed working of the instrument is mentioned elsewhere.[7]

3D organotypic (3D OT) assay

3D OTs were prepared using a protocol well established in our laboratory.[5] In brief, collagen type I (*Corning*, Bedford, USA) biomatrix populated with NOF was prepared and allowed to polymerize at 37 °C for 1 h. Afterwards, the biomatrix was cultured in serum-free DMEM with 50 µg/mL L-ascorbic acid, 0.4 µg/mL hydrocortisone, 5 µg/mL insulin, 20 µg/mL transferrin and 7.5 % bovine serum albumin (all from *Sigma*, St. Louis, USA) for 24 h. The next day, CaLH3 cancer cells were seeded on top of the biomatrix (now called OT) and allowed to attach and grow for 24 h. On the 3rd day, the OTs were lifted to air–liquid interface and cultured further for 9 days in the same medium. On day 13, they were harvested, fixed in formalin and embedded in paraffin for histological and ultrahigh-resolution imaging.

Ultrahigh-resolution imaging (URI)

Ultrahigh-resolution imaging (URI) (CytoViva™, *Auburn*, USA) was employed to assess penetration of NDs into 3D OT. CytoViva™ is a high contrast optical dark-field system which gives improved contrast and signal-to-noise ratio.[8] Nanodiamonds and other light scattering objects present as bright features on a dark background. Experiments were in duplicates and image analysis was performed using *FIJI* ImageJ.[9] Data presented is the average of five images obtained from each 3D OT and represented as mean ± standard error over mean (SEM).

Transmission electron microscopy (TEM)

The cells were cultured in a 12-well plate for 24 h and allowed to attach. The next day, cells were treated with different NDs at a concentration of 20 µg/ml for 48 h in 12-well plates, trypsinized and pelleted (300 g, 5 min). After washing with PBS twice, the cell pellet was fixed in ice-cold 2 % glutaraldehyde in 0.1 M Na-cacodylate buffer as previously described.[10] Following embedding in araldite, the cells were post-fixated in 1 % osmium tetroxide. Finally, ultrathin sections (70 nm) were cut, placed on formvar carbon-coated copper grid, stained with 1 % uranyl acetate for 2 min, and analyzed by Hitachi HT7800 TEM (*Hitachi* Japan). The image analysis for area covered by internalized NDs was performed using *FIJI* ImageJ and normalized to cell surface area. A minimum of 20 cells were quantified.

Statistical analysis

Statistical analyses were performed using the Prism 9.0 software (*GraphPad Software*, San Diego, CA, USA). Data were presented as means (±SEM), unless specified otherwise. All the quantitative data were analyzed using a two-way ANOVA with Tukey's multiple comparison tests. $p < 0.05$ was considered statistically significant.

2. Results and Discussion

2.1 Analysis of DRIFT and FTIR spectra of dipeptide functionalized NDs

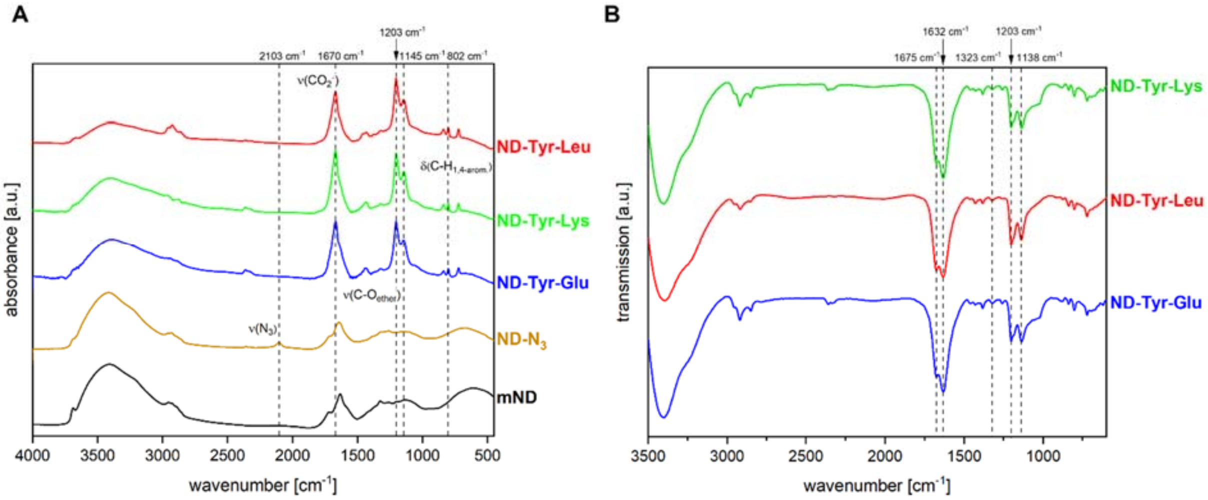

Figure S34: DRIFT (A) and FTIR (KBr pellet) spectra (B) of **ND-Tyr-Glu**, **ND-Tyr-Leu** and **ND-Tyr-Lys**, respectively. The DRIFT spectra of **mND** and **ND-N$_3$** are shown additionally in (A). Dashed lines mark significant vibrational bands.

Recorded DRIFT spectra of **ND-Tyr-Glu**, **ND-Tyr-Leu** and **ND-Tyr-Lys** present (Figure S34A) a distinctive vibrational band at 1670 cm^{-1} appearing as superposition of bands at 1675 cm^{-1} and 1632 cm^{-1} in the corresponding Fourier-transform infrared (FTIR) spectra (Figure S34B). These signals can be assigned to stretching modes of −C=O (amide I) of the peptide bond merged with the asymmetric stretching vibration of the carboxylate groups. Furthermore, the absence of the characteristic fluorenylmethyloxycarbonyl vibrational bands at 1510 cm^{-1} and 746 cm^{-1} indicates the existence of free amino groups. While these two vibrational bands correspond to the out of plane bending vibration of aromatic C−H bonds in 1,2-disubstituted aromatic rings as it can be found for fmoc, after its removal the signal of the 1,4-disubstituted tyrosine unit became more clearly visible at 802 cm^{-1} in the DRIFT spectra of **ND-Tyr-Glu**, **ND-Tyr-Leu** and **ND-Tyr-Lys**. The FTIR spectra additionally show a weak signal of the symmetric stretching vibration of $-CO_2-$ groups at 1323 cm^{-1} and two intensive bands at 1203 cm^{-1} and 1138 cm^{-1} referring to −C−O−C stretching vibrations of the tyrosyl ether. Since structure and functional groups of the surface functionalization of all three ND systems are quite similar, the respective DRIFT as well as FTIR spectra (KBr pellet) generally look very much alike.

2.2 Particle size distribution of *ND-N₃*, *ND-Tyr-Glu*, *ND-Tyr-Leu* and *ND-Tyr-Lys* as aqueous dispersion measured by dynamic light scattering

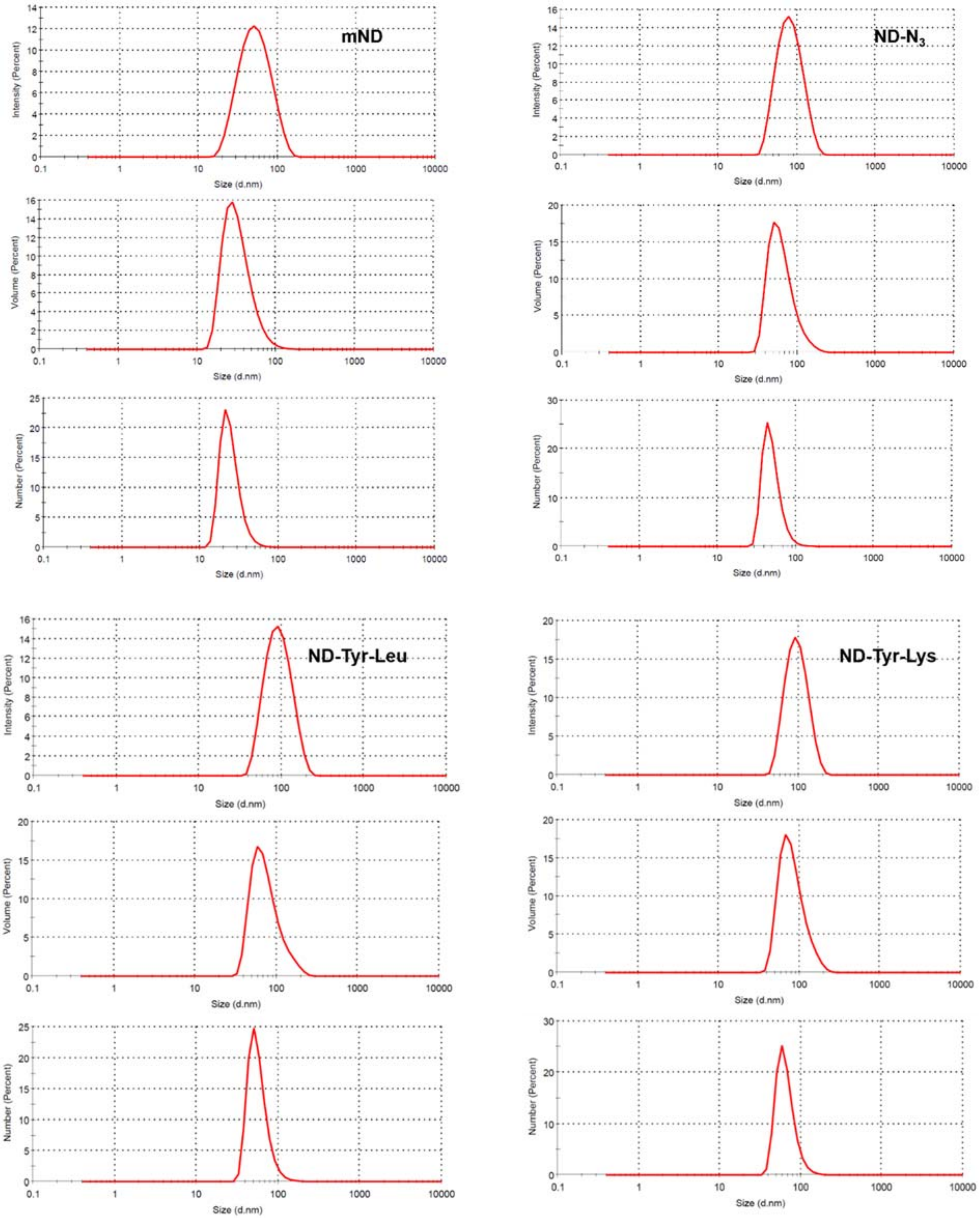

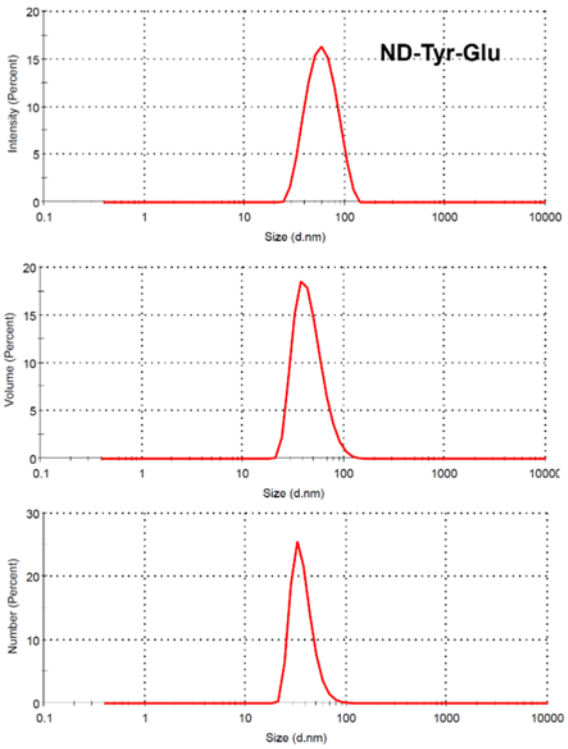

Figure S35: Dynamic light scattering spectra of **mND**, **ND-N$_3$**, **ND-Tyr-Leu**, **ND-Tyr-Lys** and **ND-Tyr-Glu** in dd-H_2O as a distribution of intensity (top), volume (middle) and number (bottom).

2.3 Particle size of *ND-Tyr-Glu*, *ND-Tyr-Leu* and *ND-Tyr-Lys* measured by AFM

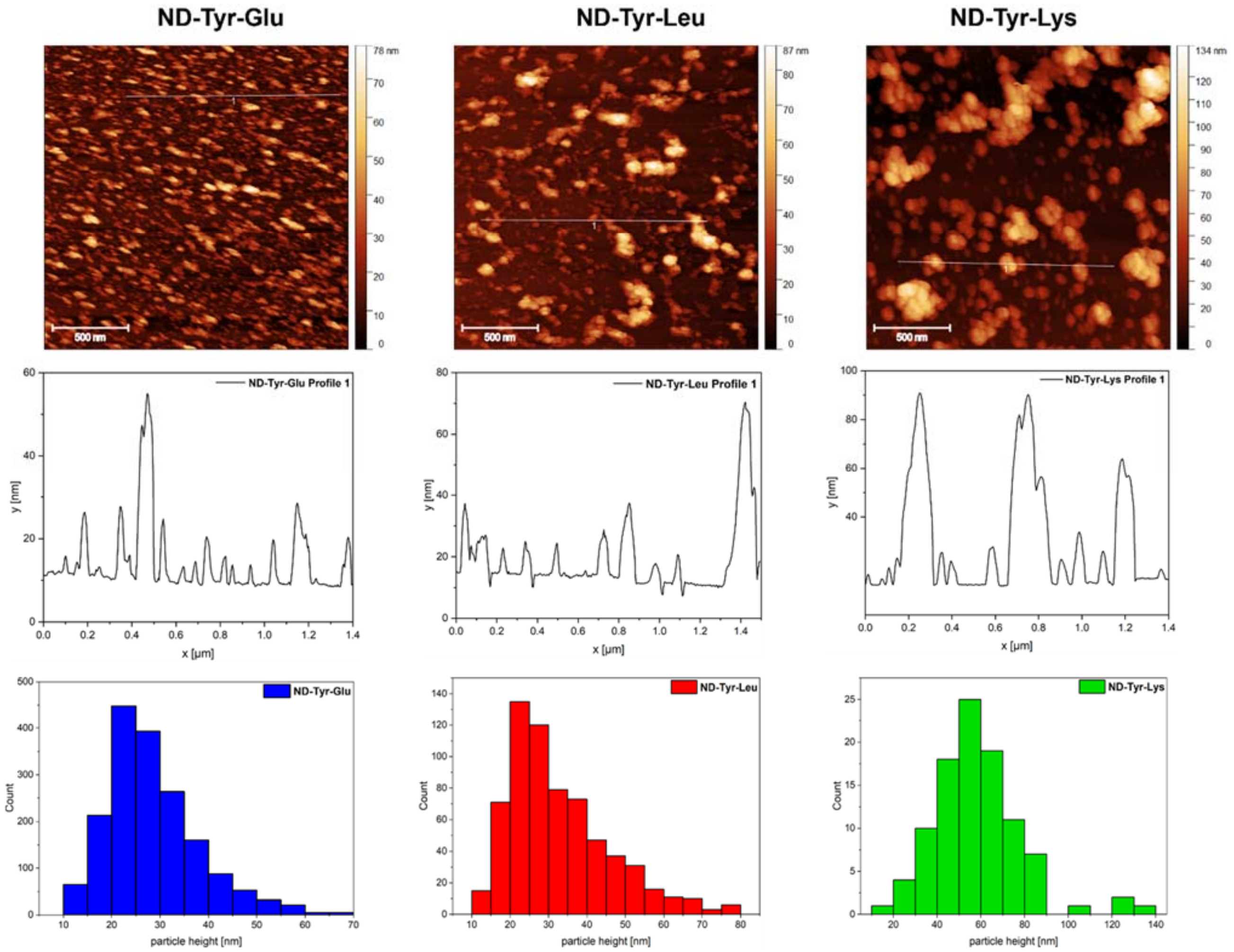

Figure S36: AFM images of **ND-Tyr-Glu** (left), **ND-Tyr-Leu** (center) and **ND-Tyr-Lys** (right) deposited on a silicon wafer by dropcasting (top row), the corresponding height profiles (middle row) and particle size distributions (bottom row).

2.4 Particle size vs. pH titration of *mND*, *ND-N$_3$*, *ND-Tyr-Glu*, *ND-Tyr-Leu* and *ND-Tyr-Lys*

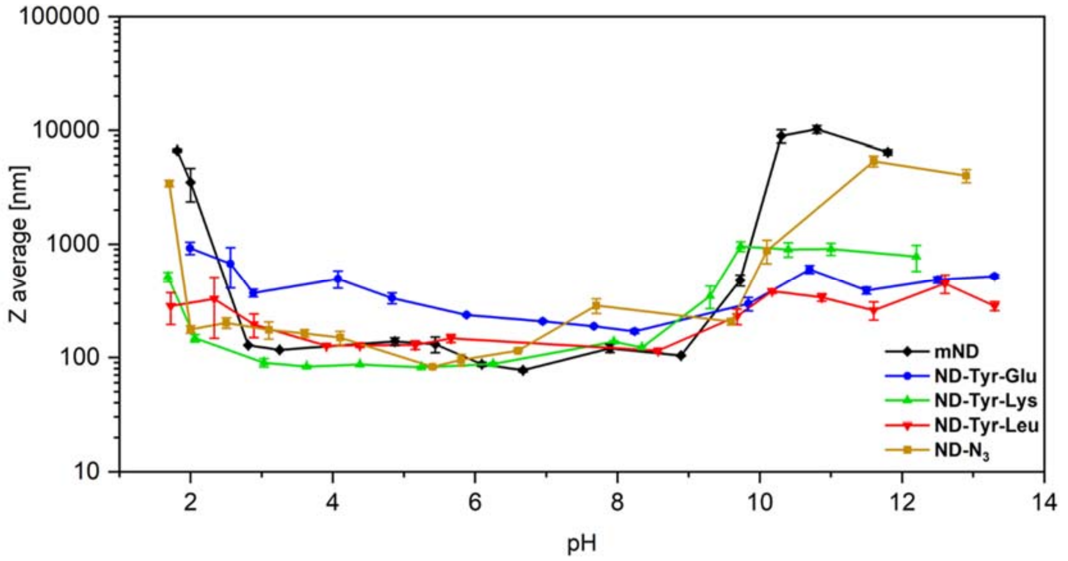

Figure S37: Titration study: evolution of the particle size (Z average) of **mND**, **ND-N$_3$**, **ND-Tyr-Glu**, **ND-Tyr-Leu** and **ND-Tyr-Lys** as function of pH. Error bars show the deviation of triplicate measurements.

2.5 Colorimetric assay (Kaiser test)

The Kaiser test was performed to prove of the existence of primary amino groups after all deprotection steps.

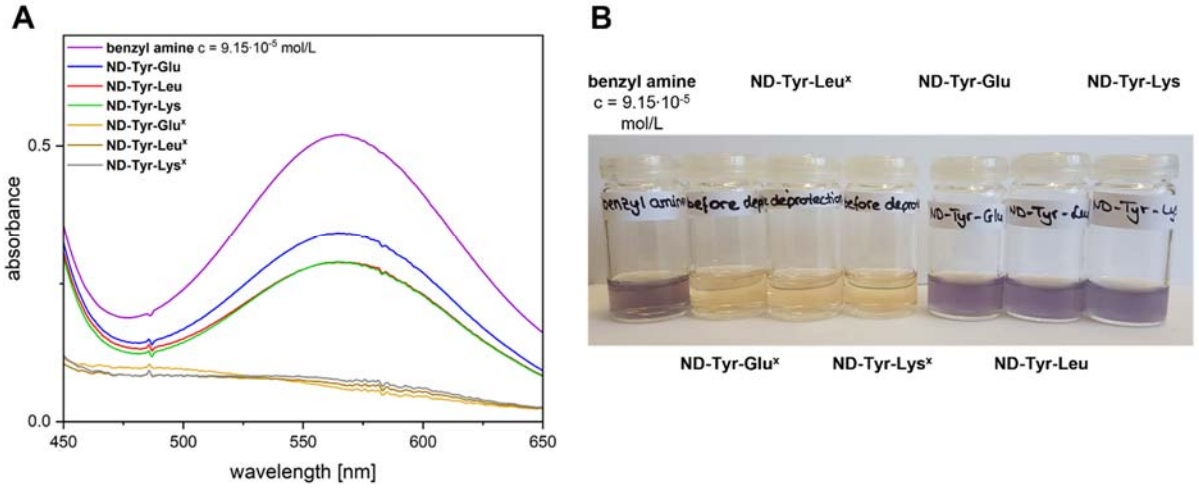

Figure S38: UV/Vis absorption spectra of dipeptide functionalized NDs before (**ND-Tyr-Glu^x**, **ND-Tyr-Leu^x** or **ND-Tyr-Lys^x**) and after cleavage of fmoc and *tert*-butyl ester protecting groups (**ND-Tyr-Glu**, **ND-Tyr-Leu** and **ND-Tyr-Lys**) as well as benzyl amine (c = 9.15·10^{-5} mol/L) as positive control in ethanol/water (3:2, v/v) (A). The characteristic absorption band with λ_{max} = 566 nm indicates the existence of primary amine groups after reaction with ninhydrin. The colour change (from yellow to purple) in case of a positive result can be seen in panel B.

2.6 Thermogravimetric analysis

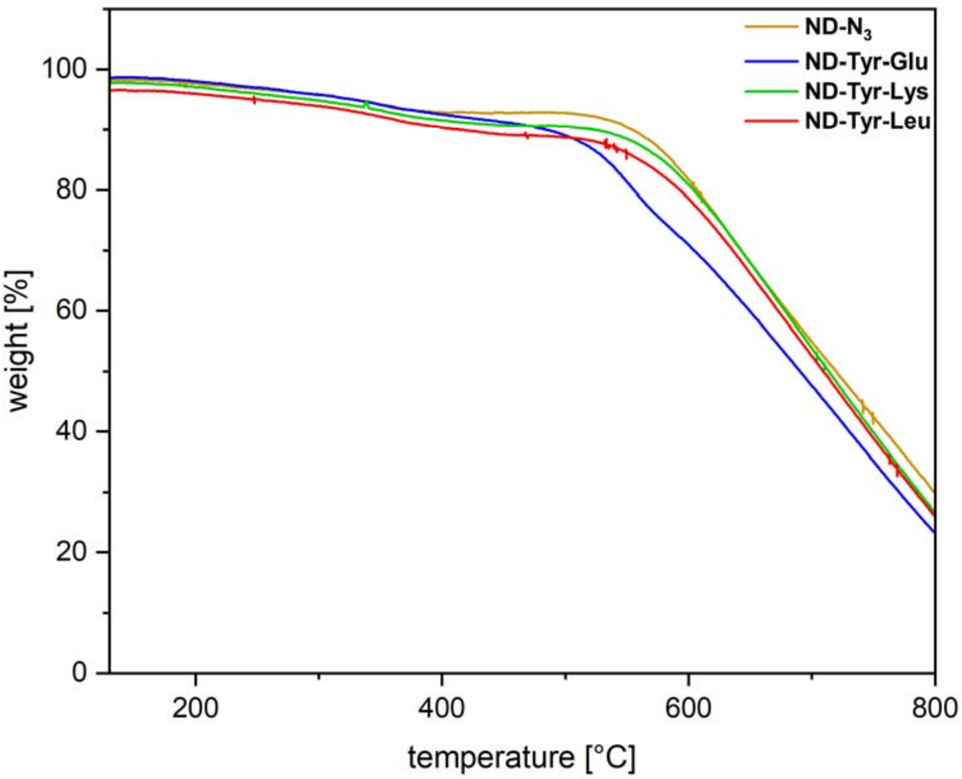

Figure S39: Thermogravimetric analysis of **ND-N$_3$**, **ND-Tyr-Glu**, **ND-Tyr-Leu** and **ND-Tyr-Lys**.

2.7 Colloidal stability of *mND*, *ND-N₃* and dipeptide functionalized NDs in different media

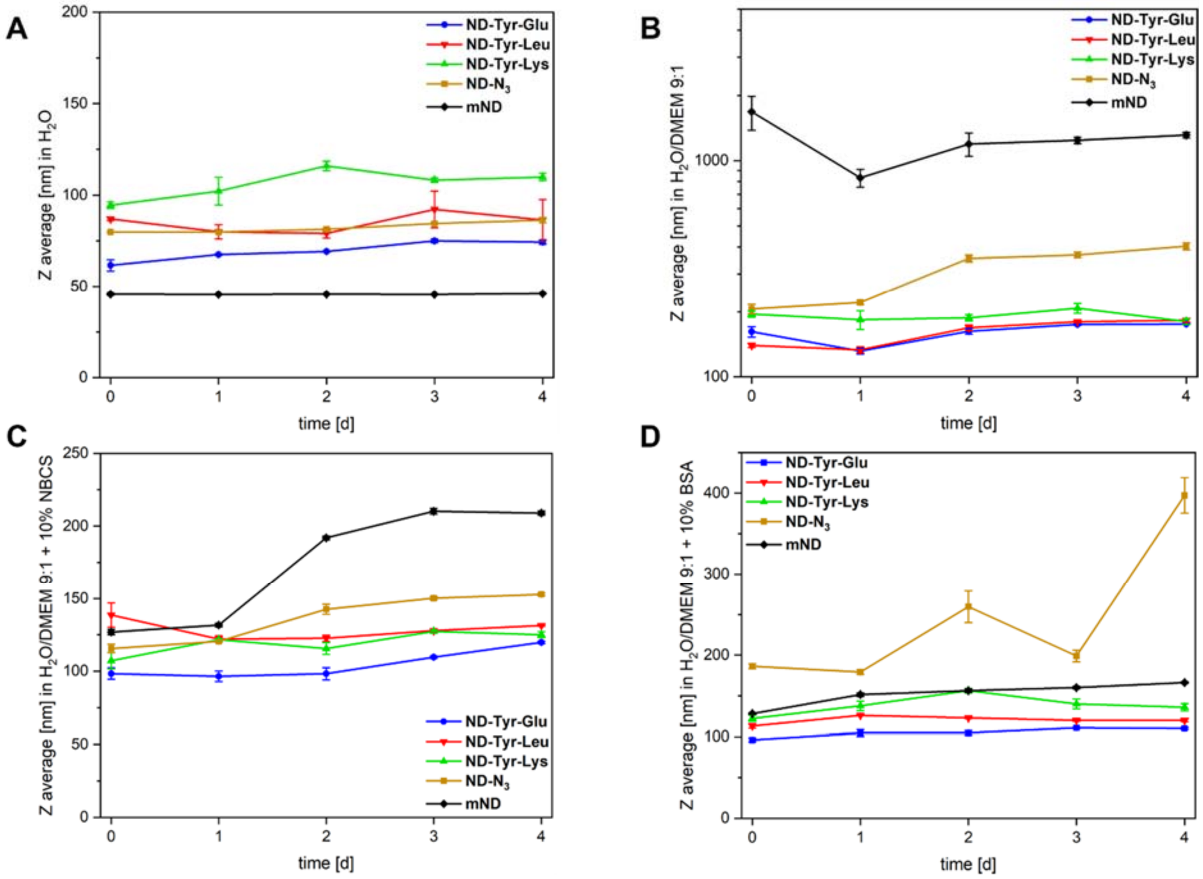

Figure S40: Panel A – D: particle size of dipeptide functionalized NDs, **mND** and **ND-N$_3$** monitored by DLS over four days in dd-H_2O (A), dd-H_2O/DMEM 9:1 (v/v) (B), dd-H_2O/DMEM 9:1 + 10 % NBCS (C) and dd-H_2O/DMEM 9:1 + 10 % BSA (D). Error bars show the deviation of triplicate measurements.

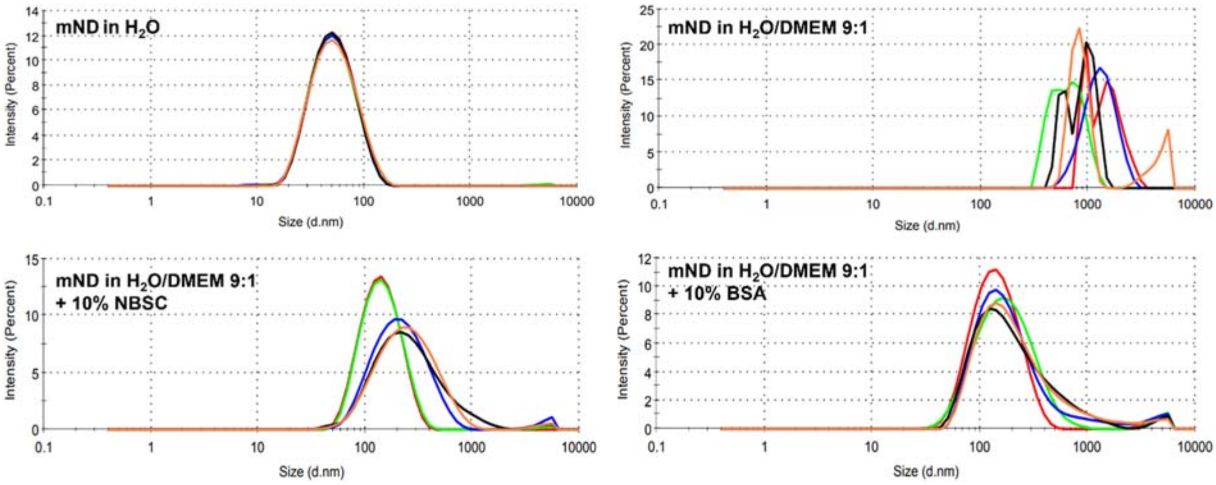

Figure S41: Dynamic light scattering spectra of **mND** as a distribution of intensity in dd-H_2O (top, left), in dd-H_2O/DMEM 9:1 (top, right), in dd-H_2O/DMEM 9:1 + 10 % newborn calf serum (NBCS) (bottom, left) and in dd-H_2O/DMEM 9:1 + 10 % bovine serum albumin (BSA) (bottom, right). Colour code: red – day 0, green – day 1, blue – day 2, black – day 3 and orange – day 4.

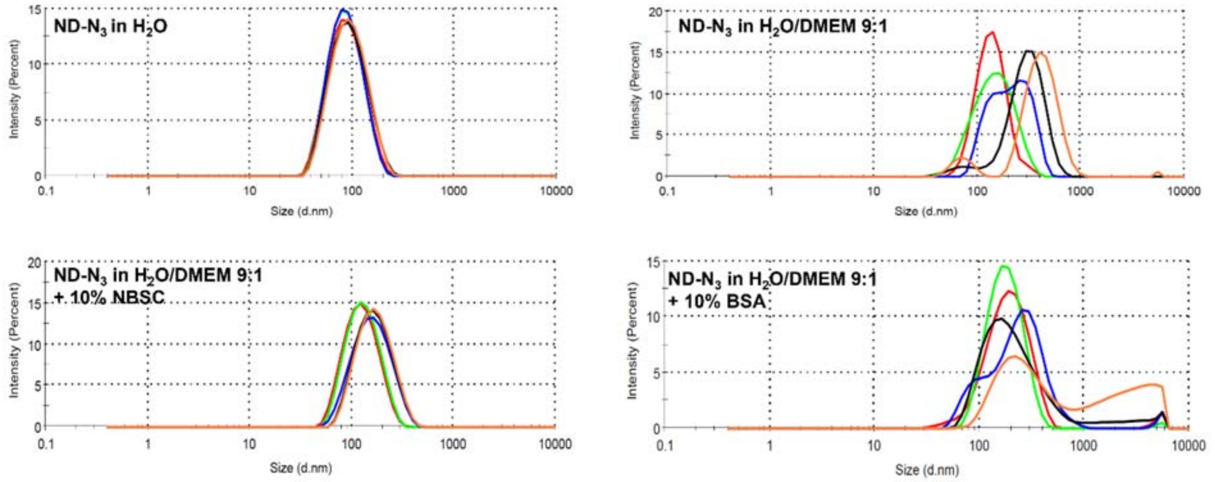

Figure S42: Dynamic light scattering spectra of **ND-N_3** as a distribution of intensity in dd-H_2O (top, left), in dd-H_2O/DMEM 9:1 (top, right), in dd-H_2O/DMEM 9:1 + 10 % newborn calf serum (NBCS) (bottom, left) and in dd-H_2O/DMEM 9:1 + 10 % bovine serum albumin (BSA) (bottom, right). Colour code: red – day 0, green – day 1, blue – day 2, black – day 3 and orange – day 4.

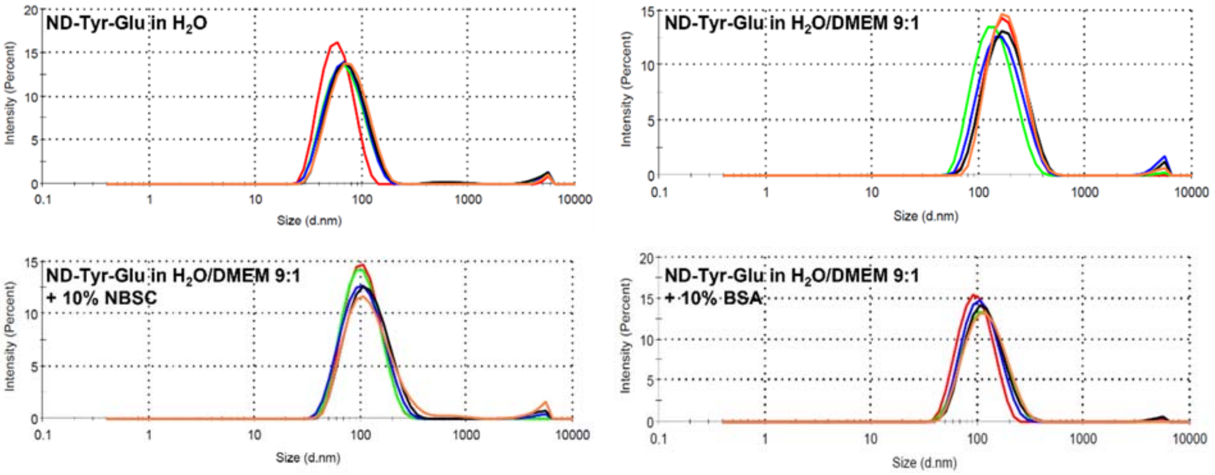

Figure S43: Dynamic light scattering spectra of **ND-Tyr-Glu** as a distribution of intensity in dd-H_2O (top, left), in dd-H_2O/DMEM 9:1 (top, right), in dd-H_2O/DMEM 9:1 + 10 % newborn calf serum (NBCS) (bottom, left) and in dd-H_2O/DMEM 9:1 + 10 % bovine serum albumin (BSA) (bottom, right). Colour code: red – day 0, green – day 1, blue – day 2, black – day 3 and orange – day 4.

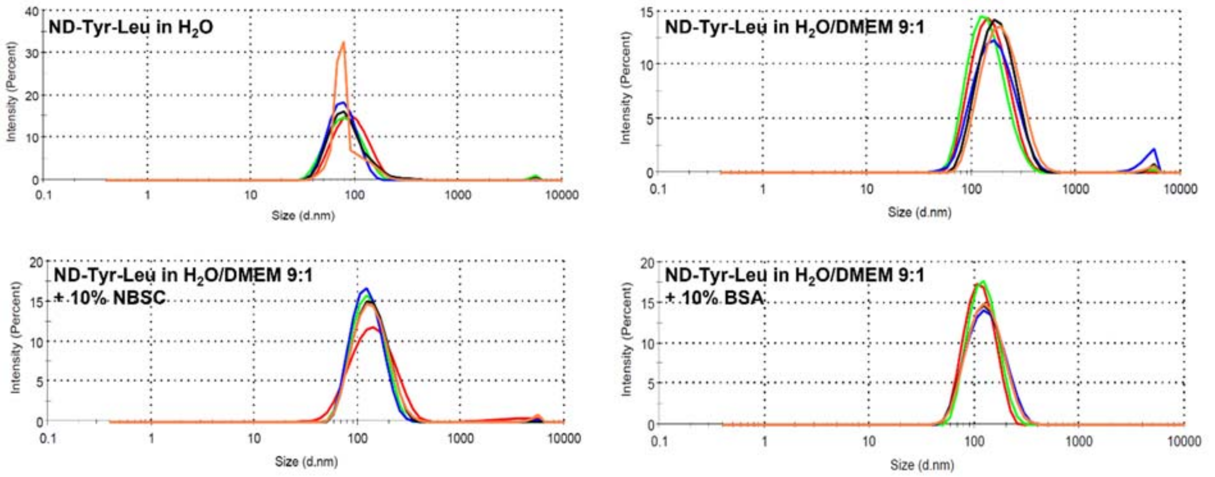

Figure S44: Dynamic light scattering spectra of **ND-Tyr-Leu** as a distribution of intensity in dd-H_2O (top, left), in dd-H_2O/DMEM 9:1 (top, right), in dd-H_2O/DMEM 9:1 + 10 % newborn calf serum (NBCS) (bottom, left) and in dd-H_2O/DMEM 9:1 + 10 % bovine serum albumin (BSA) (bottom, right). Colour code: red – day 0, green – day 1, blue – day 2, black – day 3 and orange – day 4.

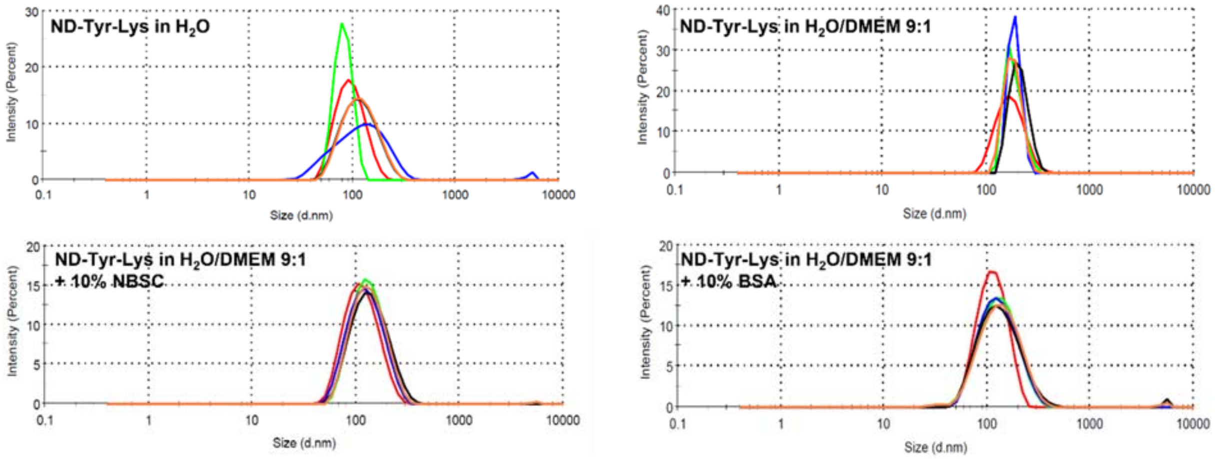

Figure S45: Dynamic light scattering spectra of **ND-Tyr-Lys** as a distribution of intensity in dd-H_2O (top, left), in dd-H_2O/DMEM 9:1 (top, right), in dd-H_2O/DMEM 9:1 + 10 % newborn calf serum (NBCS) (bottom, left) and in dd-H_2O/DMEM 9:1 + 10 % bovine serum albumin (BSA) (bottom, right). Colour code: red – day 0, green – day 1, blue – day 2, black – day 3 and orange – day 4.

2.8 Cell morphology and proliferation of CaLH3 and NOF cells after ND treatment

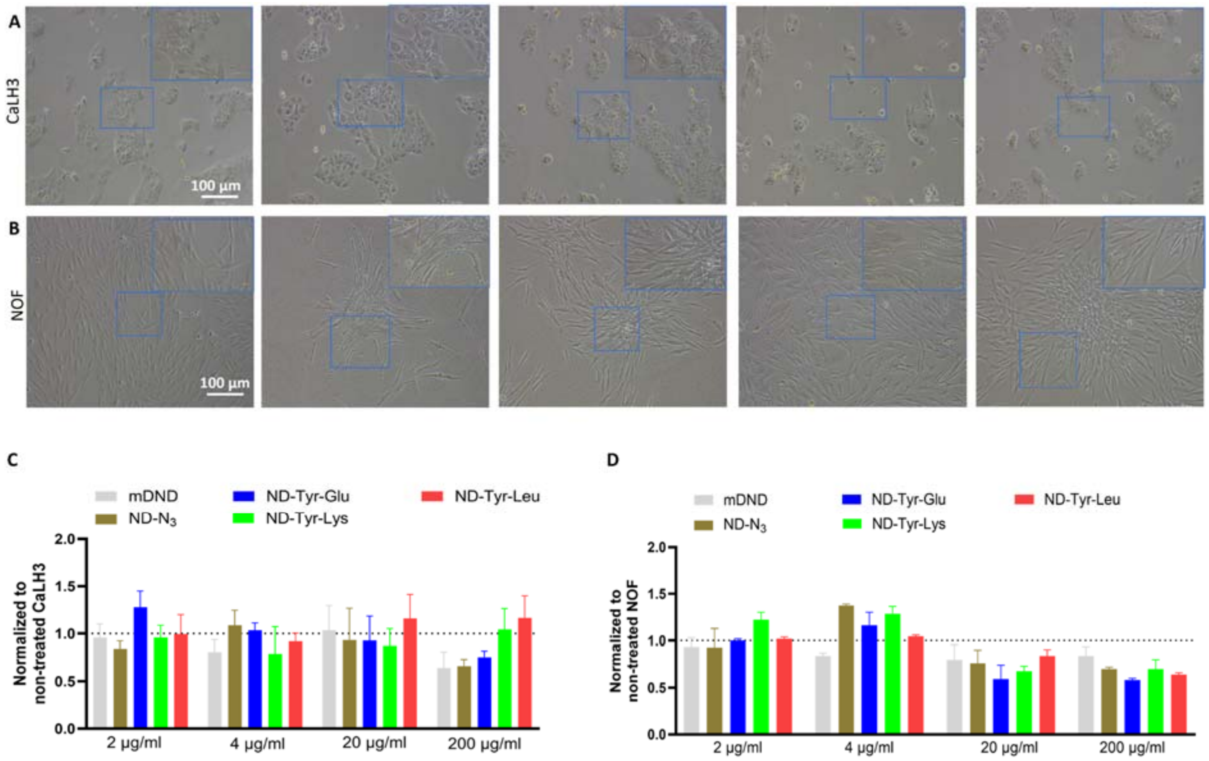

Figure S46: Cell morphology of (A) CaLH3 and (B) NOF cells as observed by phase contrast microscopy after treatment with different ND conjugates. Scale bar = 100 µm. Graphs showing proliferation of CaLH3 (C) and NOF (D) when exposed to different concentrations of different ND conjugates. The data presented here is normalized to non-treated controls at each concentration tested.

2.9 Hematoxylin-eosin stained 3D OT after exposure to different ND conjugates

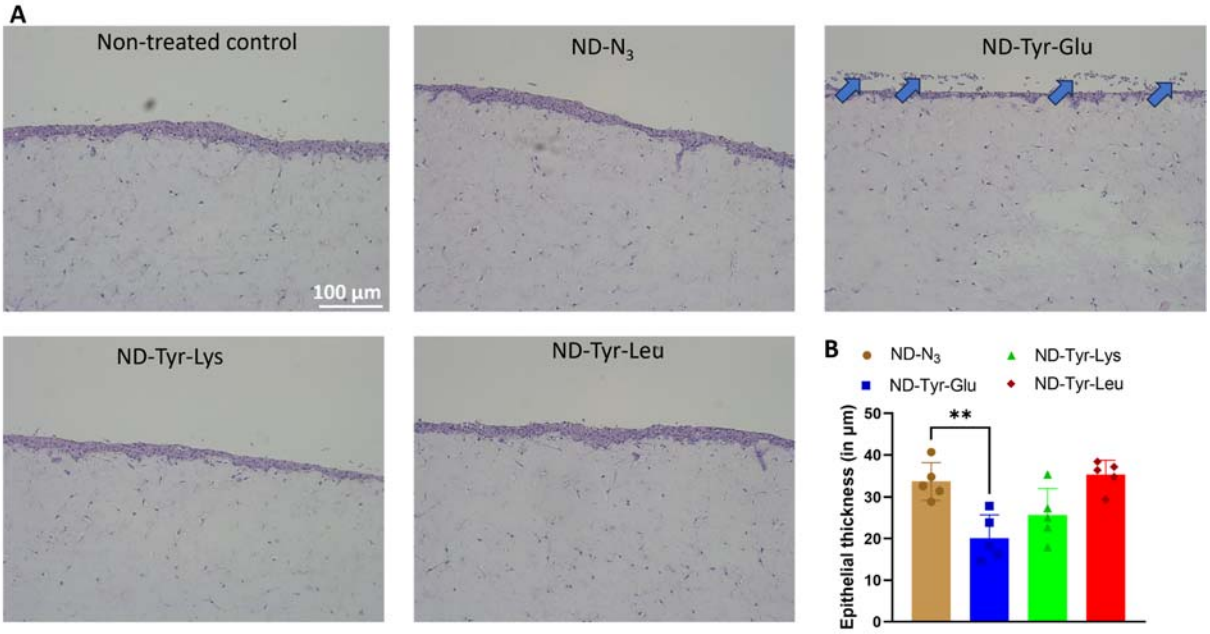

Figure S47: A: Representative hematoxylin and eosin (HE) stained images of 3D OT when exposed to different ND conjugates. Scale bar = 100 µm, blue arrows indicate detached epithelial cells due to desqumation. B: Graphical representation of epithelial thickness of 3D OTs in µm treated with different ND conjugates. **p=0.007

2.10 Ultrastructural localization of *ND-Tyr-Glu*

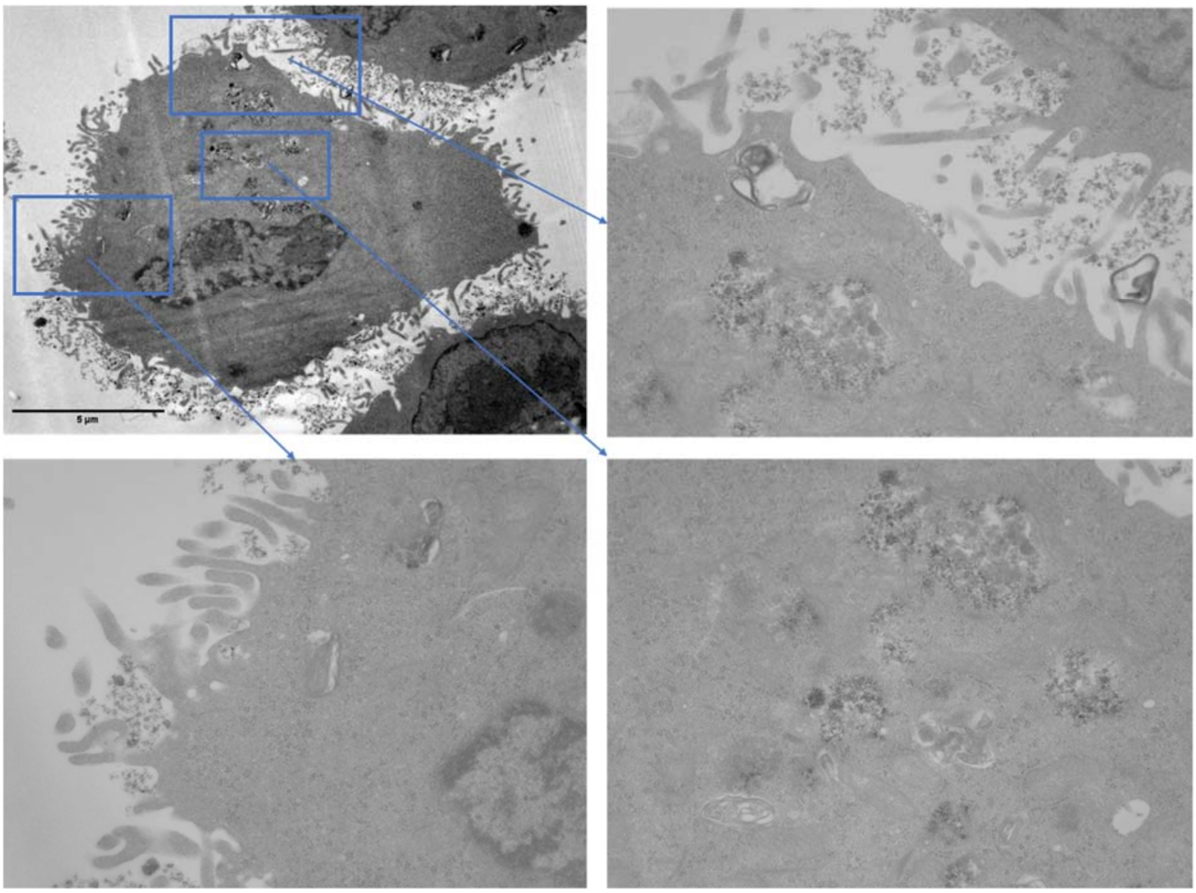

Figure S48: Ultrastructural localization of **ND-Tyr-Glu** revealed by TEM. TEM micrographs revealed localization of NDs at the cell surface and in the cytoplasm presumably internalized via macropinocytosis. NDs were not localized in the nucleus. Scale bar = 5 µm.

3. References

[1] T. Meinhardt, D. Lang, H. Dill, A. Krueger, *Adv. Funct. Mater.* **2011**, *21*, 494-500.

[2] A. Krüger, F. Kataoka, M. Ozawa, T. Fujino, Y. Suzuki, A. E. Aleksenskii, A. Y. Vul', E. Ōsawa, *Carbon* **2005**, *43*, 1722-1730.

[3] G. Jarre, S. Heyer, E. Memmel, T. Meinhardt, A. Krueger, *Beilstein J. Org. Chem.* **2014**, *10*, 2729-2737.

[4] L. J. Harper, D. E. Costea, L. Gammon, B. Fazil, A. Biddle, I. C. Mackenzie, *BMC Cancer* **2010**, *10*, 166.

[5] H. Dongre, N. Rana, S. Fromreide, S. Rajthala, I. Bøe Engelsen, J. Paradis, J. S. Gutkind, O. K. Vintermyr, A. C. Johannessen, L. Bjørge, D. E. Costea, *Exp. Cell Res.* **2020**, *386*, 111684.

[6] M. Ibrahim, Y. Xue, M. Ostermann, A. Sauter, D. Steinmueller-Nethl, S. Schweeberg, A. Krueger, M. R. Cimpan, K. Mustafa, *J. Biomed. Mater. Res. A* **2018**, *106*, 1697-1707.

[7] M. R. Cimpan, T. Mordal, J. Schölermann, Z. E. Allouni, U. Pliquett, E. Cimpan, *J. Phys. Conf. Ser.* **2013**, *429*, 012026.

[8] V. Konstantinova, M. Ibrahim, S. A. Lie, E. S. Birkeland, E. Neppelberg, M. C. Marthinussen, D. E. Costea, M. R. Cimpan, *J. Oral Pathol. Med.* **2017**, *46*, 214-222.

[9] J. Schindelin, I. Arganda-Carreras, E. Frise, V. Kaynig, M. Longair, T. Pietzsch, S. Preibisch, C. Rueden, S. Saalfeld, B. Schmid, J.-Y. Tinevez, D. J. White, V. Hartenstein, K. Eliceiri, P. Tomancak, A. Cardona, *Nat. Methods* **2012**, *9*, 676-682.

[10] S. Sawant, H. Dongre, D. Kanojia, S. Jamghare, A. Borges, M. Vaidya, *Microsc. Microanal.* **2019**, *25*, 1367-1375.

4. Author contributions

E.M. Synthesis and characterization of all organic compounds and particle conjugates, stability experiments, biological investigations, analyzing data, writing original draft, reviewing and editing manuscript

H.P. Biological investigations, analyzing data

M.-R.C. and D.-E.C. Funding acquisition, resources, supervision biological investigations, analyzing data, reviewing and editing manuscript

H.N.D. Supervision and conceptualization biological investigations, analyzing data, writing, reviewing and editing manuscript

A.K. Funding acquisition, resources, conceptualization, supervision, analyzing data, reviewing and editing manuscript
